# Tail-less precursors in synthetic cannabinoid production: investigating a clandestine laboratory, seized samples, and CB_1_ activity

**DOI:** 10.1007/s00204-025-04086-0

**Published:** 2025-05-23

**Authors:** Manuela Carla Monti, Tobias Rautio, Marie H. Deventer, Markus Schläpfer, Johannes Tveit, Alex J. Krotulski, Victoria Marland, Robert Reid, Niamh Nic Daeid, Craig McKenzie, Christophe P. Stove, Henrik Green, Caitlyn Norman

**Affiliations:** 1 Division of Clinical Chemistry and Pharmacology, Department of Biomedical and Clinical Sciences, Linköping University, Linköping, Sweden; 2 Department of Physics, Chemistry and Biology, Linköping University, Linköping, Sweden; 3 Laboratory of Toxicology, Department of Bioanalysis, Faculty of Pharmaceutical Sciences, Ghent University, Ghent, Belgium; 4 Zurich Forensic Science Institute, Zurich, Switzerland; 5 Chiron AS, Trondheim, Norway; 6 Center for Forensic Science Research and Education, Fredric Rieders Family Foundation, Horsham, PA USA; 7 Leverhulme Research Centre for Forensic Science, School of Science and Engineering, University of Dundee, Dundee, UK; 8 Department of Forensic Genetics and Forensic Toxicology, National Board of Forensic Medicine, Linköping, Sweden

**Keywords:** Synthetic cannabinoid receptor agonists, New psychoactive substances, Precursors, Clandestine drug laboratory investigation, CB_1_ cannabinoid receptor

## Abstract

An emerging new trend reveals that potent synthetic cannabinoid receptor agonists (SCRAs) that were prevalent prior to the Chinese class-wide ban in July 2021 have remained on the market or re-emerged. This is likely because of a new production route where unscheduled tail-less precursors are converted into the desired controlled SCRA via a one-step synthesis. This study reports the discovery of a clandestine production site in Switzerland for this synthesis. Controlled recreations of the synthesis of ADB-BUTINACA (also known as ADB-BINACA or ADMB-BUTINACA) and MDMB-4en-PINACA based on a recipe recovered from the Swiss production site were undertaken. The recreations yielded oils or waxy products with chromatographic purities ranging from 39.3 to 93.4% with the precursors (ADB-INACA or MDMB-INACA) remaining in all products. Mixtures of a precursor (e.g., MDMB-INACA, ADB-INACA, AB-INACA, MDMB-ICA, ADB-IATA, MDMB-5’Me-INACA) with corresponding final SCRA(s) (e.g., MDMB-4en-PINACA, 4F-MDMB-BUTINACA, MDMB-BUTINACA, MDMB-FUBINACA, ADB-BUTINACA, ADB-4en-PINACA, AB-CHMINACA, 4F-MDMB-BUTICA, ADB-FUBIATA) were found in 28 forensic casework samples in the US received between February 2023 and February 2024 and 87 samples seized from the Scottish prisons between February 2023 and July 2024. In vitro CB_1_ receptor activity of the precursors, synthesized products, and mixtures of precursors and final SCRAs were examined using β-arrestin 2 recruitment and intracellular calcium release assays. The precursors were much less potent than the final SCRAs and the concentration of the final SCRA dictated the activity of mixtures. This study raises awareness of the emergence of a new production method for SCRAs as observed in multiple countries.

**Supplementary Information:**

The online version contains supplementary material available at 10.1007/s00204-025-04086-0.

## Introduction

Synthetic cannabinoid receptor agonists (SCRAs) describe a diverse drug class of new psychoactive substances (NPS) with a total of 254 compounds monitored by the European Union Drugs Agency (EUDA), formerly known as the European Monitoring Centre for Drugs and Drug Addiction (EMCDDA), as of 2024 (EMCDDA 2024). SCRAs show activity at the cannabinoid 1 (CB_1_) receptor, which is also the main target of Δ^9^-tetrahydrocannabinol (Δ^9^-THC), the major psychoactive component of cannabis (Cannaert et al. 2016; Grafinger et al. 2021; Pike et al. 2021). Importantly, many SCRAs act as full agonists at CB_1_ and have increased potency and efficacy compared to Δ^9^-THC, which is only a partial agonist (Pulver et al. 2023; Sparkes et al. 2022). The high potencies of many SCRAs at CB_1_ have been associated with more frequent and severe adverse effects (Labay et al. 2016; Luethi and Liechti 2020). They have been reported in the context of numerous poisonings, including fatalities (Angerer et al. 2017; Boland et al. 2020; Groth et al. 2023; Labay et al. 2016), and are therefore classified as a public health threat (EMCDDA 2017, 2024). Health and social concerns are aggravated by SCRAs being more popularly used by marginalized populations, including rough sleepers and prisoners (Norman et al. 2021; Shafi et al. 2020). People who use drugs are put at a higher risk of intoxication due to the adulteration of cannabis products with SCRAs and the mis-selling of these products as regular cannabis (EMCDDA 2024; Monti et al. 2022; Oomen et al. 2022; Pulver et al. 2022a). Consumer products like adulterated cannabis are typically prepared in the countries of intended use from bulk powders bought online from China as evidenced by the discovery of processing and packaging sites around the EU (EUDA and Europol 2024).

As one of the main production countries for SCRAs, Chinese legislation has been shown to play a major role regarding the types of SCRAs circulating in other parts of the world (Norman et al. 2021; Pulver et al. 2022b). New compounds emerge every year often to circumvent new legislative controls, making the recreational market for SCRAs highly dynamic (Norman et al. 2020; Pulver et al. 2022a). In 2021, China adopted a generic analog ban covering most important SCRA classes, including the indazole- and indole-3-carboxamide SCRAs. In response, new so-called exploratory structural variations emerged (Liu et al. 2022; Pulver et al. 2022b), including SCRAs belonging to the FUPPYCA and OXIZID classes and SCRAs having an acetamide linker (ATA) or brominated core (5’Br) (Andrews et al. 2023; Deventer et al. 2022a, 2022b, 2023, 2024; Norman et al. 2024). However, as demonstrated in various studies (Deventer et al. 2022a, 2022b, 2023, 2024; Patel et al. 2024; Sparkes et al. 2024), many of these new-generation SCRAs have a considerably lower potency and efficacy at CB_1_ than earlier generations. Interestingly, despite being covered by the Chinese analog ban, some earlier-generation SCRAs, including MDMB-4en-PINACA and ADB-BUTINACA, have continued to be detected on the illicit market (Norman et al. 2024). Simultaneously, SCRAs lacking the tail moiety (“tail-less SCRAs”), such as MDMB-5’Br-INACA (Norman et al. 2024) and MDMB-INACA (Timmerman et al. 2024), were identified in seized samples, sometimes in combination with their tailed analog (e.g., MDMB-INACA with MDMB-4en-PINACA) (Timmerman et al. 2024).

SCRAs are often sold via the internet and starting around March 2022, websites were found advertising tail-less SCRAs as so-called “semi-finished” or “precursor” SCRAs. Some websites included detailed step-by-step instructions on a one-step synthesis method that is advertised as easy and not requiring sophisticated laboratory equipment for the conversion of these tail-less SCRAs or precursors (referred to as precursors going forward) into the potent earlier-generation SCRAs now internationally controlled. Some websites also offer the option to buy kits with the precursors and additional reagents required for the synthesis. Since these websites have a high turnover, they are generally only accessible for very short periods; therefore, screenshots of several examples of these websites are included in the Supplementary Information (Figs. S1.1–S1.5).

Typical instructions available on the websites describe a one-step synthesis where the precursor (typically 1 kg) is placed into a reaction vessel and heated and stirred for several hours together with dimethylformamide (DMF), potassium carbonate, and the respective brominated tail-function (e.g., 5-bromo-1-pentene for MDMB-4en-PINACA). The synthesis is stopped by precipitating the reaction mixture in a water and ice bath and filtering the solid product. This synthesis route has recently been described in a patent application in China (狄斌 et al. 2024) and has the alkylation of the core (addition of the tail) as the final step, meaning the tail-less SCRAs are precursors. This differs from previously described synthesis routes for indole and indazole SCRAs as the coupling of the alkyl side chain (“tail”) is generally conducted before the coupling of the head group, which means the tail-less SCRAs are not precursors and are not expected to be present as impurities. Thus, recent detections of earlier pre-ban SCRAs with varying amounts of precursors (e.g., MDMB-4en-PINACA together with MDMB-INACA) in seized samples (Timmerman et al. 2024) as are also reported within this study, indicate that the use of this precursor synthesis route and “semi-finished kits” has gained popularity. Furthermore, the presence of the precursor in the seized samples indicates that the synthesis might result in varying yields depending on the applied reaction conditions.

**Fig. 1:**
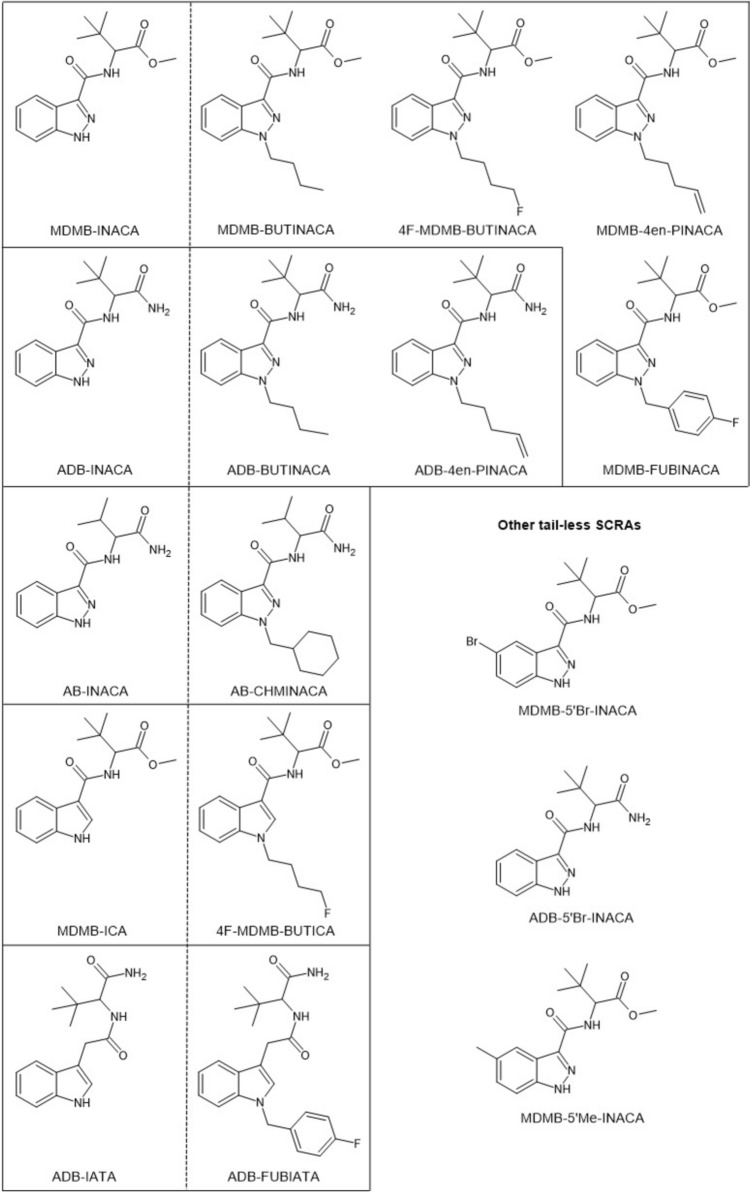
Chemical structures of the tail-less precursors and corresponding final SCRAs detected in this study

This study highlights these current developments surrounding semi-finished SCRA production kits. It reports on a clandestine production site located in Switzerland where such one-step synthesis was in use to produce SCRAs, including ADB-BUTINACA and MDMB-4en-PINACA, providing insights into the actual processes and equipment used. To gain further information on the synthesis method and expected yields, the synthesis was replicated at two independent laboratories using the precursors seized at the site. Additionally, this study reports findings on SCRA materials with varying degrees of precursors in the final products seized from Scottish prisons and prisons and forensic casework from the United States (US). The structures of all the precursors and final SCRAs detected in this study are provided in Fig. 1. Lastly, biological activity of the precursors and final products and mixtures of precursors and product reference standards that emulate those found in seized samples were assessed using two CB_1_ receptor activity bioassays, providing an evaluation of the potential pharmacological and safety profiles of the drug products.

## Materials and methods

### Clandestine laboratory investigation (Switzerland)

Upon the discovery of a clandestine drug production site in Switzerland in 2023, various chemicals and drug products were seized from the site. Analysis of the seized drug products (including synthesis products found in the reaction vessel, materials on numerous drying racks, and in plastic bags) were conducted following ISO 17025 accredited screening and confirmation procedures using gas chromatography coupled to mass spectrometry (GC–MS) and attenuated total reflection Fourier-transform infrared spectroscopy (ATR-FTIR). For the GC–MS analysis, approximately 1 mg of powder samples was dissolved in 1 mL ethyl acetate (Carl Roth Chemikalien, Arlesheim, Switzerland). For herbal material treated with SCRAs, approximately 200 mg of the plant material was placed directly into a 5 mL syringe and washed with 1000 µL of ethyl acetate to dissolve potential SCRAs on the surface. The sample was then filtered using a 0.2 µM syringe filter obtained from B.Braun (Sempach, Switzerland).

Analysis was conducted using a 7890B GC coupled to a 5977B MS (Agilent Technologies, Basel, Switzerland). Injection mode: 1 µL sample injection was used with a 25:1 split into a 4 mm internal diameter deactivated glass liner pre-packed with quartz wool, injection port temperature: 280 °C, carrier gas: He, flow: 1.1–1.5 mL/min (retention time locked to C24). Column: DB-5MS, 30 m × 0.25 mm × 0.25 µm (Agilent Technologies). GC oven: 80 °C held for 0 min; 15 °C /min to 320 °C held for 8 min; total run time: 24 min; transfer line: 280 °C. The MS was operated in electron impact (EI) mode with the following ionization conditions: 70 eV in full scan mode (30–650 amu), ion source: 230 °C, quadrupole: 150 °C. The ATR-FTIR measurements were conducted using an ALPHA FTIR spectrometer equipped with the platinum ATR module (Bruker, Fällanden, Switzerland). Between 5 and 10 mg of the powdered sample was placed on the diamond of the crystal plate. The spectra were generated from 24 scans measured from 400 to 4000 cm^−1^ wavelengths at a resolution of 0.9 cm^−1^. Background spectra were generated for every measurement series and subtracted. The resulting ATR-FTIR and GC–MS spectra were compared to either in-house reference spectra obtained by measuring certified reference standards from Cayman Chemical (obtained through LGC Standards GmbH, Wesel, Germany) or by comparison to spectra shared through the ADEBAR project (Pulver et al. 2022a).

ADB-INACA and MDMB-INACA powders seized from the clandestine laboratory were provided by the Zürich Forensic Institute (Zürich, Switzerland) to the laboratories in Sweden and Norway to replicate the synthesis (see Sections "Precursor synthesis replication (Sweden)" and "Precursor synthesis replication (Norway)").

### Synthesis of reference standards (Sweden)

ADB-INACA, MDMB-INACA, ADB-BUTINACA, and MDMB-4en-PINACA were synthesized at purities > 95%, as described previously (Rautio et al. 2024, 2025). Reference standards were analytically confirmed using nuclear magnetic resonance (NMR) spectroscopy and the results can be found in the Supplementary Information. Calibrated methanolic solutions were prepared from the powder references standards and provided to the laboratories in Sweden and Belgium conducting in vitro activity profiling (Sections "AequoScreen® CB1 intracellular Ca2+ release assay (Sweden)", and "NanoBiT® CB1 β-arrestin 2 recruitment assays (Belgium)").

### Precursor synthesis replication (Sweden)

For the synthesis at Linköping University, dimethylformamide (DMF) was obtained from VWR international (Stockholm, Sweden), potassium carbonate from Alfa Aesar (Karlsruhe, Germany), 4-bromobutane from TCI Europe N.V (Zwijndrecht, Belgium), and 5-bromo-1-pentene from Fluorochem (Hadfield, United Kingdom). For both ADB-INACA and MDMB-INACA, each precursor (5.00 g, 18.2 mmol) was dissolved in DMF (15 mL), followed by the addition of potassium carbonate (3.60 g, 26.0 mmol) and 4-bromobutane (2.37 g, 17.3 mmol) to ADB-INACA or 5-bromo-1-pentene (2.46 g, 16.5 mmol) to MDMB-INACA. Three reaction conditions were employed: i) stirring at room temperature (RT) for 5 h, (ii) stirring at 70 °C for 5 h, and (iii) stirring at 70 °C for 10 h. The reaction mixture was poured into ice water and left to solidify. The ADB-INACA series resulted in waxy solids that were filtered and washed three times with ice-cold water, then the solid was collected and left to air-dry for four days. The MDMB-INACA series resulted in oils that were extracted further by dichloromethane (DCM; 2 × 100 mL), then the organic layers were combined and washed with water (50 mL). The combined organic layers were dried with magnesium sulfate (MgSO_4_), filtered, and the solvent removed in vacuo. The resulting crude oil was dried over vacuum for four days.

### Precursor synthesis replication (Norway)

For the synthesis at Chiron AS, DMF was obtained from Merck (Schnelldorf, Germany), potassium carbonate was obtained from Alfa Aesar (Karlsruhe, Germany), 5-bromo-1-pentene was purchased from BLDpharm (Kaiserlautern, Germany), and 4-bromobutane was obtained from Fluorochem (Hadfield, United Kingdom). The precursors ADB-INACA (5.00 g, 18.2 mmol) or MDMB-INACA (5.00 g, 17.3 mmol) were mixed with potassium carbonate (4.25 g, 30.8 mmol) and suspended in 15 mL DMF. The mixture was added to either 5-bromo-1-pentene (for MDMB-INACA, 2.70 mL, 25.0 mmol)) or 4-bromobutane (for ADB-INACA, 2.70 mL, 22.8 mmol). The same three reaction conditions as in Sweden were employed: (i) stirring at RT for 5 h, (ii) stirring at 70 °C for 5 h, and (iii) stirring at 70 °C for 10 h. The reaction mixture was poured into ice water. For the ADB-INACA series, waxy solids were obtained that were filtered and left to air-dry at RT for three weeks. For the MDMB-INACA series, the resulting products were oils, which required further extraction. The water was decanted off and the residue oil was diluted in DCM (40 mL) and washed with brine (40 mL). The phases were separated, and the organic phase was dried over MgSO_4_. The solids were filtered off and the DCM removed under reduced pressure. The resulting oils were left to air-dry at RT for three weeks.

### Analytical characterization of precursor synthesis products (Sweden and Norway)

The precursors (ADB-INACA and MDMB-INACA) seized from the clandestine laboratory and synthesis products from Sweden and Norway were all analyzed in Norway using ultra-high-performance liquid chromatography (UHPLC)-diode array detector (DAD)-MS and GC-flame ionization detector (FID) with MS. UHPLC-DAD-MS analysis was performed using a 1290 series UHPLC coupled to a 1260 series DAD and 6130 series MS (Agilent Technologies). The autosampler was held at 15 °C and column oven held at 40 °C. The mobile phases used were (A) ammonium formate (2 mM) in water with 0.1% (*v/v*) formic acid and (B) ammonium formate (2 mM) in MeOH. The gradient used was 95:5 A:B from 0 to 9 min; 0:100 A:B from 9 to 10.1 min; and 95:5 A:B from 10.1 to 12 min. The flow rate was 0.55 mL/min and 0.2–2 μL of sample was injected onto a Raptor biphenyl column 100 mm × 2.1 mm, 2.7 μm particle size column (Restek, Bellefonte, PA, USA). The DAD was operated with a wavelength of 210 nM, scan range of 190–640 nm, and a sample rate of 20 Hz. MS was operated in positive and negative electrospray ionization (ESI + /ESI−) with a gas flow of 12 L/min, nebulizer pressure of 35 psi, gas temperature of 350 °C, capillary voltage of 3000 V, fragmentor voltage of 150 V, and mass range of 100–1000 mass-to-charge ratio (*m/z*).

GC–FID–MS was performed using an 8890 series GC with FID coupled to a 5977B MS (Agilent Technologies). Injection mode: 1 µL sample injection was used with a 20:1 split into a 4 mm internal diameter ultra-inert glass liner pre-packed with glass-frit, injection port temperature: 250 °C, carrier gas: He, flow: 1.2 mL/min. Column: HP-5MS, 30 m × 0.25 mm × 0.25 µm (Agilent Technologies). GC oven: 50 °C held for 4 min; 20 °C /min to 315 °C held for 10 min; total run time: 27.75 min; transfer line: 300 °C. The FID was operated at 350 °C with a hydrogen fuel flow of 1.5 mL/min, air flow of 250 mL/min, and makeup flow of 20 mL/min. MS was operated in EI mode with the following ionization conditions: 70 eV in full scan mode (50–800 m*/z*), ion source: 230 °C, quadrupole: 150 °C.

The chromatographic purity of the synthesis products was calculated based on the average of triplicate analyses using HPLC and GC-FID. The ADB-BUTINACA products are only based on HPLC analysis as ADB-INACA and ADB-BUTINACA co-eluted on the GC-FID system.

The synthesis products from Sweden were also analyzed by NMR performed using a Bruker 500/126 MHz instrument (25 °C) with CDCl_3_ as the solvent. The synthesis products from Norway were analyzed by NMR performed using a Bruker 400 MHz Avance Neo (Bruker, Billerica, MA, USA) equipped with a 5 mm SmartProbe (^1^H) and DMSO-d6 as the solvent. Results of the HPLC–DAD-MS, GC-FID-MS, and NMR analyses can be found in the Supplementary Information (Sect. 5).

### Seized sample analysis (USA)

Acetonitrile (ACN) and methanol (MeOH) were obtained from Honeywell Chemicals (Charlotte, NC, USA) and were of LC–MS grade purity. Ammonium formate (99%) was obtained from Alfa Aesar (Ward Hill, MA, USA). Formic acid ampoules (1 mL) were obtained from Thermo Fisher Scientific (Waltham, MA, USA). 10,11-dihydrodibenz[*b,f*][1,4]oxazepin-11-one (BBOP) was obtained from Sigma-Aldrich (Burlington, MA, USA). Standard reference materials for SCRAs and their precursors were purchased from Cayman Chemical (Ann Arbor, MI, USA) and prepared at 1 mg/mL in MeOH or ACN.

Drug samples analyzed for SCRAs originated from prisons, drug checking, or law enforcement and consisted of infused paper (n = 17), herbal materials (n = 12), liquid in tianeptine bottles (n = 2), and unspecified status (n = 3). Samples were prepared via MeOH dilution (approximately 1 mg to 2 mL) or via acid/base extraction (approximately 1 mg was added to water, HCl and NaOH were added for pH adjustment, and DCM was used as the extraction solvent). For analysis by GC–MS, 0.1 mL of an internal standard solution (0.5 mg/mL BBOP and *N*-propyl amphetamine) was added followed by additional dilution in mobile phase (1:100 *v:v*) for ultra-high-performance liquid chromatography (UHPLC)-quadrupole time-of-flight (QToF)-MS analysis.

GC–MS analysis was performed using a 5975 series GC–MS system (Agilent Technologies). Injection mode: 1 µL sample injection was used with a splitless glass liner, injection port temperature: 265 °C, carrier gas: He, flow: 1 mL/min. Column: Zebron™ Inferno™ ZB-35HT, 15 m × 250 μm × 0.25 μm. GC oven: 60 °C held for 0.5 min; 35 °C/min to 340 °C held for 6.5 min; total run time: 15 min; transfer line: 300 °C. The MS operated in EI mode. Ionization conditions: 40–550 m*/z*, 250 threshold, ion source: 230 °C, quadrupole: 150 °C.

UHPLC–QToF–MS analysis was performed using a Nexera XR UHPLC (Shimadzu, Columbia, MD, USA) with the autosampler held at 15 °C and column oven held at 30 °C coupled to a TripleTOF® 5600 + QToF (Sciex, Framingham, MA, USA). The mobile phases used were (A) ammonium formate (10 mM, pH 3.0) and (B) 50:50 ACN:MeOH (*v:v*) with 0.1% formic acid. The gradient used was 95:5 A:B to start, inverted to 5:95 A:B over 13 min and held for 1 min; returned to 95:5 A:B. The flow rate was 0.4 mL/min and 10 μL of sample was injected into a Kinetex C_18_ 50 mm × 3.0 mm, 2.6 μm particle size column (Phenomenex, Torrance, CA, USA). The QToF was operated in ESI + mode with an IonSpray Voltage Floating of 2,500 eV and a source temperature of 600 °C. Precursor ion masses were acquired by TOF MS scan from 100 to 510 m*/z*. Precursor ions were filtered using SWATH® acquisition (27 windows) and fragmented using a collision energy (CE) spread (35 ± 15 eV). SWATH® acquisition is a data independent acquisition mode that allows for comprehensive detection of product ions regardless of known precursor ion mass at the time of acquisition, as well as increased MSMS specificity due to the windowed isolation of precursor ions in Q1. The method employed 27 overlapping variable isolation windows across the TOF MS range (e.g., 110–130 Da, 129–160 Da, 159–170 Da … 416–450 Da, 449–479 Da, and 478–510 Da). Product ion masses were acquired by MSMS scan from 50 to 510 m*/z*. The total run time was 15.5 min.

For samples where a mixture of compounds was present, the percentage total peak area for each compound was determined by comparing the peak areas of each compound to the total peak area of all active components in the sample. The percentage peak area was then corrected to account for the different EI-MS detector response of each compound by analyzing the samples alongside a mixture prepared from reference materials at the same concentrations (1 ng/µL). Based on a comparison of the peak areas of the reference materials, correction factors were calculated and applied to the peak areas of each compound in a sample. The correction factors applied for each compound can be found in Table S3.1 in the Supplementary Information.

### Seized sample analysis (UK)

MeOH (LC–MS grade) was purchased from Fisher Scientific, UK; bupivacaine and formic acid were obtained from Sigma Aldrich (Poole, UK). MDMB-BUTINACA, MDMB-INACA, and AB-INACA (> 98% purity) were obtained from Cayman Chemicals (Ann Arbor, MI, USA). AB-CHMINACA, ADB-4en-PINACA, ADB-BUTINACA, MDMB-4en-PINACA, and MDMB-FUBINACA (> 98% purity) were synthesized and supplied by the Sutcliffe Group at Manchester Metropolitan University, Manchester, UK as described previously (Antonides et al. 2019, 2021; Kronstrand et al. 2022).

Samples were extracted and analyzed by GC–MS as described previously (Antonides et al. 2021; Kronstrand et al. 2022; Marland et al. 2024; Norman et al. 2020). In brief, 2 × 1 cm^2^ samples taken from opposite corners of the paper or card were extracted in 0.5 mL of 0.25 mg/mL bupivacaine in MeOH by ultrasonication (5 min), where bupivacaine was used as an internal standard. For powder samples, approximately 10 mg of the material was vortexed (1 min) in 1 mL 0.25 mg/mL bupivacaine in MeOH. For solid powder rock-like samples, part of the sample was cut off using a scalpel and then crushed using a mortar and pestle, and approximately 10 mg of the resulting powder was vortexed (1 min) in 1 mL 0.25 mg/mL bupivacaine in MeOH. The e-liquid samples were in sealed vape pods, so the e-liquid was not easily accessible for pipetting. Instead, 1 mL 0.25 mg/mL bupivacaine in MeOH was pipetted through the mouthpiece of the vape pod into a beaker and then the vape pod was sonicated (5 min) in the solution. The extracts or supernatants (for powders and tablets) were analyzed using GC–MS.

GC–MS analysis was performed using a 7820A GC coupled to a 5977E MS (Agilent Technologies, Santa Clara, CA, USA). Injection mode: 1 µL sample injection was used with a 20:1 split into a 4 mm internal diameter deactivated glass liner pre-packed with quartz wool, injection port temperature: 200 °C, carrier gas: He, flow: 1 mL/min. Column: HP-5MS, 25 m × 0.2 mm × 0.33 µm (Agilent Technologies). GC oven: 80 °C held for 3 min; 40 °C/min to 300 °C held for 11 min; total run time: 22.5 min; transfer line: 295 °C. MS was operated in EI mode with the following ionization conditions: 70 eV in full scan mode (50–550 m*/z*), ion source: 230 °C, quadrupole: 150 °C.

The criteria for compound identification by GC–MS have been described previously (Kronstrand et al. 2022). In brief, compounds were identified by comparison of the GC–MS retention time and mass spectra for the seized samples to that of the reference material analyzed under the same instrumental conditions within 24 h of the sample. To be considered a match, the sample GC retention time must fall within 0.05 min of the retention time of the appropriate reference material, or 0.1 min for samples with a high concentration of the compound (as assessed by peak area) causing peak shape distortion and shifting of the peak apex.

For samples where a mixture of compounds was present, the percentage total peak area for each compound was determined in the same way as the US samples. The percentage peak area was then corrected to account for the different EI-MS detector response of each compound by analyzing the samples alongside a mixture prepared from reference materials at the same concentrations (100 μg/mL). Based on a comparison of the peak areas of the reference materials, correction factors were calculated and applied to the peak areas of each compound in a sample. The correction factors applied for each compound can be found in Table S3.1 in the Supplementary Information. A heat map of the seizure data was created in R (version 4.3.2) as described previously (Norman et al. 2021). The complete datasets used for the creation of the heat map can be found in the Supplementary Information (Tables S7.1 and S7.2).

### NanoBiT® CB_1_ β-arrestin 2 recruitment assays (Belgium)

Dulbecco’s modified Eagle’s medium (DMEM) (GlutaMAX™), Opti-MEM I Reduced Serum, trypsin–EDTA (0.05%), penicillin, streptomycin, and amphotericin B were purchased from Thermo Fisher Scientific. Fetal bovine serum (FBS) and poly-D-lysine were obtained from Sigma-Aldrich (Darmstadt, Germany). The Nano-Glo® Live Cell reagent and the Nano-Glo® LCS Dilution buffer were procured from Promega (Madison, WI, USA). MeOH and ACN, used for preparation of the stock solutions, were from Chem-Lab NV (Zedelgem, Belgium) and Biosolve (Valkenswaard, the Netherlands), respectively. The reference standard for (-)-CP55,940 (purity ≥ 98%) was from Cayman Chemical and JWH-018 was procured from LGC. The reference standards for ADB-BUTINACA, MDMB-4en-PINACA, ADB-INACA, and MDMB-INACA were synthesized and provided by Linköping University as described in Section "Synthesis of reference standards (Sweden)" (Rautio et al. 2024, 2025).

The development of the stable cell line, used for the activity-based βarr2 recruitment assays, has been reported previously (Cannaert et al. 2016, 2017, 2018). In short, human embryonic kidney (HEK) 293 T cells stably expressing the CB_1_-βarr2 system were routinely maintained under humidified atmosphere at 37 °C and at 5% CO_2_ in DMEM (GlutaMAX™) supplemented with 10% heat-inactivated FBS, 100 IU/mL penicillin, 100 µg/mL streptomycin, and 0.25 µg/mL amphotericin B. On the day prior to the assay, cells were trypsinized and seeded in white opaque-walled poly-d-lysine coated 96-well plates at 5 × 10^4^ cells/well and incubated overnight. Test solutions were prepared by serial dilution in Opti-MEM I Reduced Serum containing 50% MeOH and used within the next 24 h. For assessment of different mixtures of precursor (ADB-INACA or MDMB-INACA) and final SCRA (ADB-BUTINACA or MDMB-4en-PINACA), the ratios 95:5, 75:25, 50:50, and 25:75 precursor:final SCRA were prepared with the combined molarities consistent with those typically run on the bioassay. The pure precursors (100:0) and final SCRAs (0:100) were run for comparison. For seized products (ADB-INACA and MDMB-INACA) and synthesis products from Sweden (Section "Precursor synthesis replication (Sweden)"), stock solutions were prepared in MeOH and diluted accordingly for generation of full concentration–response curves.

On the day of the assay, cells were rinsed twice with 150 µL Opti-MEM, after which 100 µL was added to each well to serve as assay medium. The Nano-Glo® Live Cell Reagent (containing the furimazine substrate) was diluted 20-fold in Nano-Glo® LCS Dilution Buffer according to the manufacturer’s protocol, and 25 µL of this mix was added to each well. The plate was immediately placed into the TriStar^2^ LB 942 Multimode Reader (Berthold Technologies GmbH and Co., Germany) to measure luminescence for approximately 10 min (initial equilibration phase). After stabilization of the signal, 10 µL of 13.5 × concentrated test solutions was added and luminescence was monitored for 2 h. Appropriate solvent controls, as well as a concentration range of CP55, 940, used for further normalization of the obtained data, were taken along on each plate. Also, the prototypical SCRA JWH-018 was analyzed to allow more easy comparison with historic data obtained for other SCRAs (Cannaert et al. 2020; Wouters et al. 2019) and facilitate the inter-assay comparison with the AequoScreen® assays, where JWH-018 was used as the reference for normalization.

Absolute luminescence signals were corrected for inter-well variability in Microsoft Excel 2019 using data obtained during the initial equilibration period. Area under the curve (AUC) values were calculated for each concentration of the test compounds and values were then blank-corrected by subtracting AUC values of the appropriate solvent control. Data were normalized to the maximal receptor activation (E_max_) observed for the reference compound CP55,940, arbitrarily set at 100%. Results are represented as the AUC ± standard error of the mean (SEM) derived from a minimum of three independent experiments (*n* ≥ 3), run in duplicate. Data points for the highest concentrations were excluded in case of a reduction of at least 20% compared to the closest lower dilution as this may indicate solubility issues or cell toxicity. GraphPad Prism (Version 9.3.0; San Diego, CA, USA) was used to generate concentration–response curves and EC_50_ (potency) and E_max_ (efficacy) values were calculated by curve fitting via nonlinear regression (three-parameter logistic fit). Outliers were detected using the Grubbs test and were omitted from the dataset if applicable (p value < 0.05; applicable for 6 out of 1134 data points). Brown–Forsythe and Welch ANOVA tests (α = 0.05) were performed in GraphPad Prism to compare the EC_50_ and E_max_ values between mixtures of precursors and final products and the products of the replicated synthesis from Sweden.

### AequoScreen® CB_1_ intracellular Ca^2+^ release assay (Sweden)

The AequoZen recombinant Chinese hamster ovary (CHO) K1 cell line, stably expressing human CB_1_ (ES-110-A), was obtained from Revvity (Sollentuna, Sweden). DMEM/Ham F12 without phenol red, trypsin, and FBS was purchased from Thermo Fisher Scientific (Gothenburg, Sweden) and HEPES buffer, L-glutamine, protease-free bovine serum albumin (BSA), digitonin, and adenosine-5’-triphosphate disodium salt hydrate (ATP) were procured from Sigma-Aldrich (Stockholm, Sweden). The coelenterazine substrate was from Nanolight Technology (Pinetop, AZ, US). JWH-018 was purchased from Chiron AS (Trondheim, Norway). The ADB-BUTINACA, MDMB-4en-PINACA, ADB-INACA, and MDMB-INACA reference standards were synthesized in-house as described in Section "Synthesis of reference standards (Sweden)" (Rautio et al. 2024, 2025).

The herein used AequoScreen® technology and protocol for CB_1_ activity profiling has been described before (Åstrand et al. 2020; Truver et al. 2020). CHO-K1 cells stably expressing CB_1_, the apoaequorin enzyme and the Ga16 subunit, were maintained under humidified atmosphere at 37 °C and 5% CO_2_ in Ham’s F12 medium, supplemented with 10% heat-inactivated FBS. To perform the assays, cells were trypsinized (10 min, 37 °C), centrifuged (at 400 ×g, 5 min, RT), counted, and resuspended at 3 × 10^5^ cells/mL in DMEM/Ham’s F12 without phenol red, supplemented with 15 mM HEPES, l-glutamine, and protease-free BSA (0.1%) (further referred to as assay medium). The coelenterazine substrate was added to a final concentration of 2.5 µM and the suspension was incubated for 3 h (RT, rotating, protected from light).

Test solutions were prepared by serial dilution in assay medium and then added to white, opaque-welled 96-well plates. For assessment of different mixtures of precursor (ADB-INACA or MDMB-INACA) and final SCRA (ADB-BUTINACA or MDMB-4en-PINACA), the ratios 95:5, 75:25, 50:50, and 25:75 precursor:final SCRA were prepared with the combined molarities consistent with those typically run with the assay. The pure precursors (100:0) and final SCRAs (0:100) were run for comparison. For seized products (ADB-INACA and MDMB-INACA) and synthesis products from Sweden (Section "Precursor synthesis replication (Sweden)"), stock solutions were prepared in MeOH and diluted accordingly for generation of full concentration–response curves. JWH-018 was included as a reference on each plate. Digitonin (67 µM) and ATP (6.7 µM) were included on each plate and served as positive controls for coelenterazine loading, as both of these compounds are involved in the non-CB-dependent release of calcium ions. Blank assay medium was used as a negative control. Using a TECAN Spark 10 M plate reader (Männedorf, Switzerland), 50 µL of the incubated cell suspension was dispensed into each well (approximately 15 × 10^3^ cells/well) of the 96-well plate containing the test solutions at reading cycle #10. Luminescence was measured for 25 s (corresponding to 190 additional reading cycles).

Absolute luminescence signals were corrected for intra-plate variability in Microsoft Excel 365 using AUC values and were calculated for each concentration of the test compounds. Values were then blank-corrected by subtracting AUC values of the mean of the blank controls. Data were normalized to the maximum of JWH-018 and inter-plate variability was adjusted using the reference values. The normalized values were transferred to GraphPad Prism (Version 10.0.2) to generate concentration–response curves and calculate EC_50_ and E_max_ values by curve fitting via nonlinear regression (three-parameter logistic fit). Results are represented as receptor activity of JWH-018 [%] derived from a minimum of three independent experiments (n ≥ 3), run in triplicate. Brown–Forsythe and Welch ANOVA tests (α = 0.05) were performed in GraphPad Prism to compare the EC_50_ and E_max_ values between mixtures of precursors and final products and the products of the replicated synthesis from Sweden.

## Results and Discussion

### Clandestine laboratory investigation

**Fig. 2:**
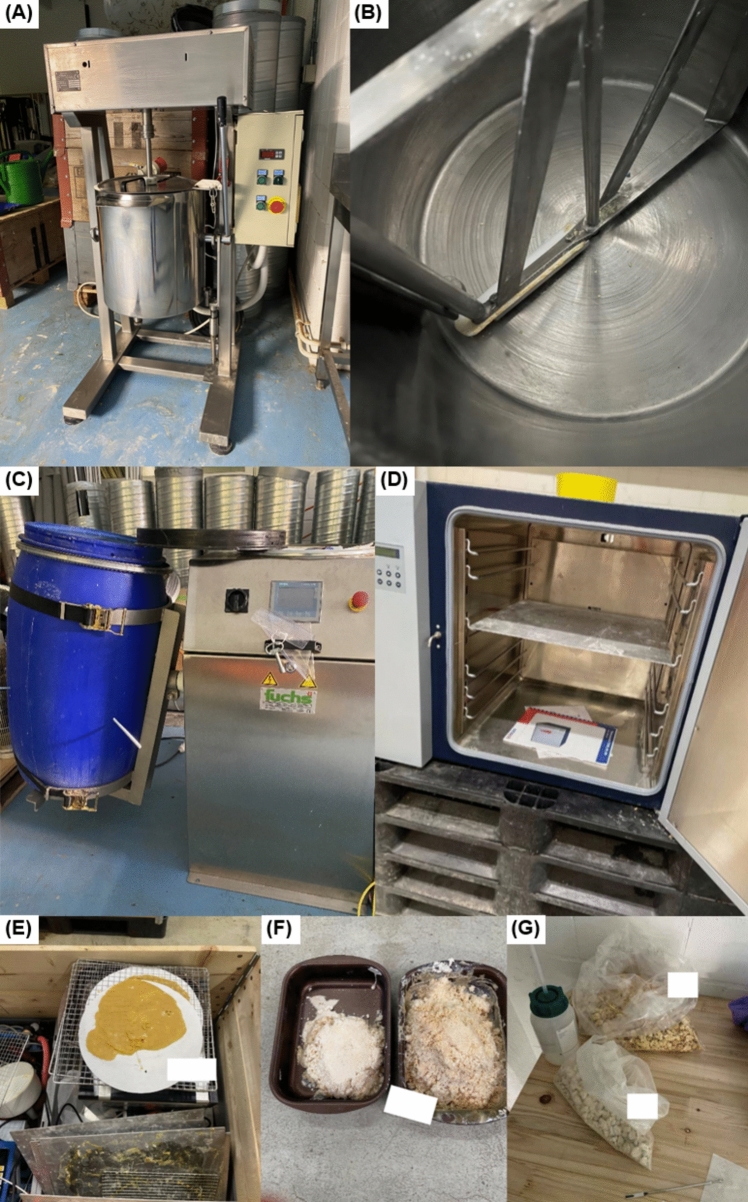
Pictures from the clandestine production site: **a** Industrial cooker with automatic stirring mechanism used for one-step synthesis. **b** Picture of the inside of the reaction vessel showing the stirrer and powder residue along the edges confirmed to be ADB-BUTINACA. **c** Tilting drum blender used for the precipitation step that was found positive for ADB-BUTINACA. **d** Laboratory oven used for drying the final product. **e** Final product on filter paper found positive for ADB-BUTINACA and drying racks. **f** Baking trays with final product found positive for ADB-BUTINACA. **g** Final product in plastic bags (Pictures by the Zurich Forensic Science Institute)

At the clandestine laboratory in Switzerland, the precursors ADB-INACA and MDMB-INACA were identified in bulk along with large quantities of chemicals and reagents, including potassium carbonate, DMF, and 4-bromobutane. These chemicals are consistent with those included in the semi-finished kits sold online (see Figs. S1.2–S1.4). Various industrial instruments were also found on site, including an industrial cooker with automatic stirring mechanism (Fig. 2A, B), a tilting drum blender (Fig. 2C), and a laboratory drying oven (Fig. 2D). The industrial cooker was used for the initial reaction, which requires heating and stirring the components for several hours. The powder residue found in the industrial cooker was found positive for ADB-BUTINACA without the precursor ADB-INACA. The barrel on the tilting drum blender was filled with ice water and used for the final precipitation step and was also found positive for ADB-BUTINACA. The products were then filtered and dried using the oven and drying racks (Fig. 2E) or baking trays (Fig. 2F). Various trays with final (converted) products were analyzed and the presence of ADB-BUTINACA was confirmed in the product on the filter papers and baking trays. The final dried product was then transferred to plastic bags (Fig. 2G), which were confirmed to be ADB-BUTINACA. Although these results demonstrate that the laboratory set-up was used for ADB-BUTINACA production at the time of discovery, the presence of other precursors, such as MDMB-INACA, and SCRAs, such as MDMB-4en-PINACA, indicates that the same set-up has likely been used for the synthesis of other SCRAs before.

**Fig. 3:**
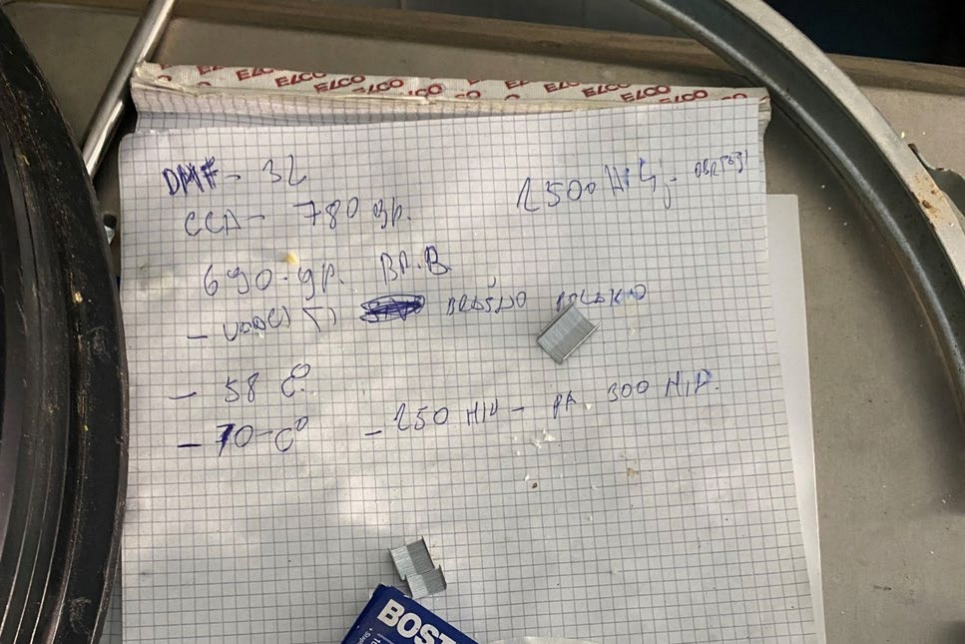
Handwritten notes on the synthesis procedure found at the clandestine laboratory (Picture by the Zurich Forensic Science Institute)

A handwritten note stating some instructions, including temperature settings, chemicals and quantities, was also found at the site (Fig. 3). The note included a list of chemicals and quantities: 3 L DMF, 780 g potassium carbonate (abbreviated as CCA), and 690 g 4-bromobutane (abbreviated as BrB). These chemicals and quantities are consistent with recipes found online for 1 kg of starting material (precursor) although there were different amounts of potassium carbonate and 4-bromobutane stated in different recipes (see Figs. S1.1–S1.5). Additionally, two different temperatures were noted: 58 °C and 70 °C, where 70 °C is consistent with the reaction temperature in online recipes. The purpose of 58 °C is unclear, but it may be the temperature when one or more of the reagents is added.

A second, related site at a different location, connected to the production laboratory and dedicated to the further processing of the resulting products was uncovered during the criminal investigation. There, the SCRAs were dissolved in organic solvent and sprayed onto low-THC cannabis material using an airbrushing apparatus, resulting in infused cannabis products prepared to mimic regular (high-THC) cannabis. Drying racks for the resulting cannabis products and vacuum sealers were found and infused cannabis products predominantly with ADB-BUTINACA or MDMB-4en-PINACA were seized at site. Infused cannabis products with ADB-BUTINACA or MDMB-4en-PINACA were also seized from the perpetrator’s residence, alongside bags of powders containing mixtures of four different SCRAs (See Table S4.2 for more information).

### Precursor synthesis replication

The precursors seized from the clandestine laboratory in Switzerland were found to be highly pure with chromatographic purities of 96.7% for ADB-INACA and 98.2% for MDMB-INACA. These precursors were used to replicate the one-step synthesis method at two independent laboratories (one in Sweden and one in Norway) employing three different reaction conditions: RT for 5 h, 70 °C for 5 h, and 70 °C for 10 h. All reaction conditions worked, but with different yields and chromatographic purities, as displayed in Table 1. Complete purity data, HPLC chromatograms, and example GC–FID chromatograms for the precursors and the replicated synthesis products can be found in the Supplementary Information.

**Table 1:** Yields and chromatographic purities of the products of the synthesis from the precursor replicated at two independent laboratories (one in Sweden and one in Norway) employing different synthesis durations (5 and 10 h) and temperatures (room temperature (RT) and 70 °C)

SCRA	Conditions	Sweden synthesis			Norway synthesis		
		Yield		Purity (%)	Yield		Purity (%)
		g	%		g	%	
ADB-BUTINACA	RT 5 h	5.21	90	86.3	4.55	76	56.9
	70 °C 5 h	7.18	125	75.5	4.61	77	39.3
	70 °C 10 h	5.30	92	92.6	4.60	76	65.2
MDMB-4en-PINACA	RT 5 h	6.05	102	89.8	3.26	53	51.6
	70 °C 5 h	5.82	98	93.9	3.95	64	58.0
	70 °C 10 h	5.54	93	91.8	3.30	53	60.4

Purity is based on the average of triplicate analyses using HPLC and GC-FID. Complete data can be found in the Supplementary Information (Table S5.1). The ADB-BUTINACA products are only based on HPLC analysis as ADB-INACA and ADB-BUTINACA co-eluted on the GC–FID

**Fig. 4:**
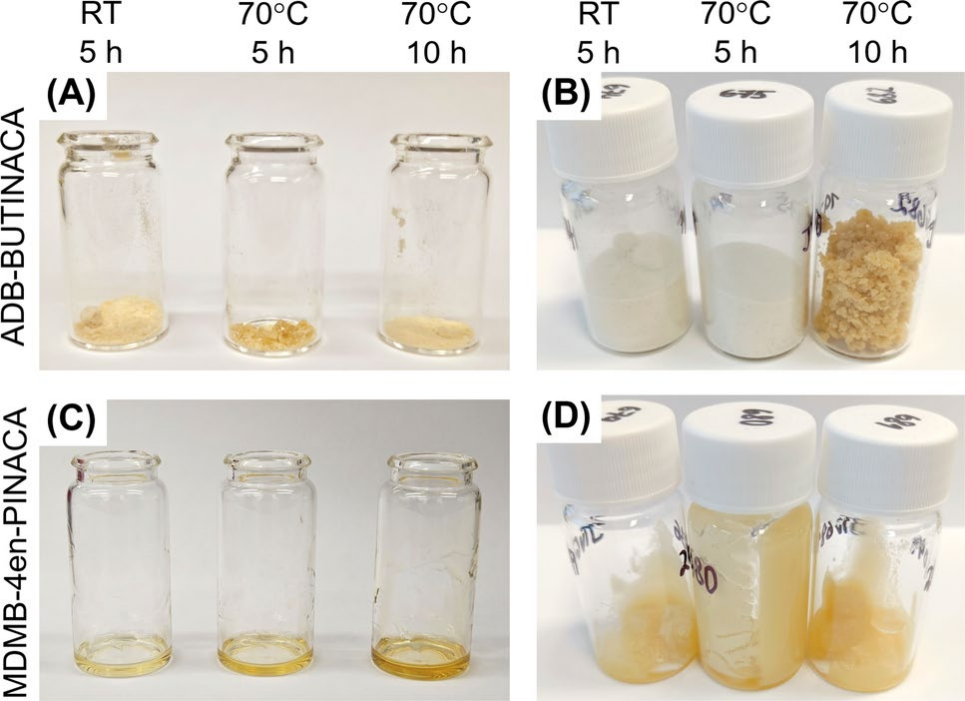
Pictures of the products from the replicated synthesis with three different reaction conditions (RT 5 h, 70 °C 5 h, and 70 °C 10 h): **a** ADB-BUTINACA products from Sweden, **b** ADB-BUTINACA products from Norway, **c** MDMB-4en-PINACA products from Sweden, and **d** ADB-BUTINACA products from Norway

The consistencies of the products also varied as shown in Fig. 4. For both laboratories, the ADB-BUTINACA synthesis products were of various waxy consistencies, where some were able to be pulverized into powders and others were not. The different consistencies are likely due to differing amounts of DMF remaining in the products, where the waxy products trapped more DMF during drying. For example, from the Sweden synthesis, the 70 °C 5 h product had the greatest amount of DMF remaining and had the waxiest consistency. This trapped DMF adds to the mass of the product and therefore is the likely reason for the high yield of this product (Table 1). It should be noted that DMF exposure via dermal absorption, oral ingestion, or inhalation is known to lead to toxicity, so its presence in these products could lead to additional adverse effects from their use (Kim and Kim 2011).

On the other hand, the MDMB-4en-PINACA synthesis using the MDMB-INACA precursor resulted in oils for all the reaction conditions investigated (Fig. 4C, D), meaning that the finished product could not be filtered off as described in the instructions found online (Figs. S1.3 and S1.4). The products from Norway partially solidified, as can be seen in Fig. 4D, so it is a mixture of oil and solid. It is suspected that the solidified portions were unreacted precursor (MDMB-INACA). One synthesis instruction found online mentioned the possibility of an “oily finished product” and stated that there is no influence on the quality of the product (Fig. S1.4).

### Seized sample analysis

#### United States

In the US, 77 seized samples received for testing between November 2021 and February 2024 were found to contain at least one SCRA and 44.2% (n = 34) contained a precursor. The samples were either paper (n = 17), herbal material (n = 12), or liquid in tianeptine bottles (n = 2) although the sample format is unspecified for three samples. The complete data can be found in the Supplementary Information (Table S6.1). Tail-less SCRAs were first detected on their own (without a corresponding final SCRA) with five detections between November 2021 and January 2023: two detections of ADB-5’Br-INACA, one of MDMB-5’Br-INACA, and two of ADB-INACA. All of these samples were from near the time of the precursors’ first detection on the illicit market, so were likely from the period of initial market testing of these new SCRAs prior to the shift to their use as precursors. Therefore, they will not be discussed further.

The first detection of a precursor in combination with a corresponding final SCRA was in February 2023. Since then, of the samples containing a precursor, 28 samples (96.6%) contained a mixture of a precursor with one or two corresponding final SCRA(s), where eight samples contained two different precursors and corresponding final SCRA mixtures. As shown in Table 2, MDMB-INACA was detected alongside MDMB-4en-PINACA in 17 samples, MDMB-4en-PINACA and 4F-MDMB-BUTINACA in 2 samples, and MDMB-BUTINACA in 1 sample. ADB-INACA was detected with ADB-BUTINACA and ADB-4en-PINACA in 5 samples, ADB-BUTINACA in 4 samples, and ADB-4en-PINACA in 3 samples. MDMB-ICA was detected with 4F-MDMB-BUTICA in 3 samples and ADB-IATA with ADB-FUBIATA in 1 sample. Of the mixture between the precursor and final SCRA(s), the corresponding final SCRA(s) was present at between 31.0 and > 99.9% of the sample, with an average of 88.2%.

**Table 2:** Tail-less precursors and final SCRA mixtures detected in 28 samples from the US and the percentage of the final SCRA in comparison to the corresponding precursor in the sample based on the percentage peak areas corrected for EI-MS detector response

Precursor	Final SCRA(s)	n	% Final SCRA vs precursor		
			Average	Min	Max
MDMB-INACA	MDMB-4en-PINACA	17	87.1	31.0	99.9
	MDMB-4en-PINACA + 4F-MDMB-BUTINACA	2	99.6	99.4	99.8
	MDMB-BUTINACA	1	88.1	–	–
ADB-INACA	ADB-BUTINACA + ADB-4en-PINACA	5	91.3	68.0	99.3
	ADB-BUTINACA	4	68.3	41.3	92.0
	ADB-4en-PINACA	3	92.4	88.4	99.1
MDMB-ICA	4F-MDMB-BUTICA	3	99.9*	99.9*	99.9*
ADB-IATA	ADB-FUBIATA	1	> 99.9*	–	–

The n values are greater than the total number of samples as eight samples contained two different precursors and corresponding final SCRA mixtures

^*^The % of peak area for these samples has not been corrected for the non-linear EI-MS detector response as the reference standard for the precursor was not available for analysis at the time of the study

Since February 2023, there was only one sample found to contain a precursor on its own: a sample containing MDMB-5’Me-INACA from August 2023. This was the first detection of MDMB-5’Me-INACA in the US, so similar to the first detections of other tail-less SCRAs, this could be from a period of initial market testing. In future, MDMB-5’Me-INACA may be used as a precursor to make other SCRAs, such as MDMB-5’Me-BUTINACA or MDMB-4en-5’Me-PINACA, although there is no evidence that these have been detected to date.

#### Scotland

In the Scottish prisons, ADB-5’Br-INACA and MDMB-5’Br-INACA were the first tail-less SCRAs detected, with 8 and 9 detections, respectively, across 12 samples seized between February and November 2021 (see Norman et al. (2024) for more information). However, these tail-less SCRAs were never detected alongside a corresponding final SCRA and appeared on the market apparently prior to the use of the “semi-finished kits” synthesis method; therefore, these samples will not be discussed further.

There were also 16 samples where MDMB-INACA was detected without a corresponding final SCRA (e.g., MDMB-4en-PINACA). One of those samples also contained MDMB-5’Br-INACA and two samples also contained the non-corresponding SCRA ADB-BUTINACA without the presence of ADB-INACA. All these samples were seized in 2023, with all but one seized between February and July. These samples were likely from the period of initial market testing of these new SCRAs as since then, the tail-less SCRAs have only been detected in combination with a corresponding final SCRA, indicating a shift to their use as precursors. However, this could be due to low sample numbers as there were only 24 SCRA samples from 2024 analyzed in comparison to 154 samples from 2023.

The first tail-less precursor SCRA detected in a mixture with a corresponding final SCRA was MDMB-INACA in a sample seized on 13th March 2023, although MDMB-INACA was first detected on its own in a sample seized on 7th February 2023. Between 7th February 2023 and 22nd July 2024, there were 558 samples from 429 seizures seized from 11 of the 17 Scottish prisons analyzed. At least one SCRA was detected in 174 samples (31.2%), of which 103 samples (59.2%) contained a precursor. Of those, 87 samples (84.5%) contained a mixture of a precursor with one or two corresponding final SCRA(s). Most samples were either powders (n = 35) or e-cigarette cartridges (n = 32), which typically have other sample types inside, including waxy- or putty-like materials (see Timmerman et al. 2024 for more information), powders, and papers as can be seen in the example examination photographs in Fig. S7.3. Other sample types detected were waxy- or putty-like materials (n = 13), paper (n = 5), and solid powder rock-like samples (n = 2). Example examination photographs of each sample type can be found in the Supplementary Information (Figs. S7.1–5) and the complete detection and analytical data can be found in the Supplementary Information (Tables S8.1 and S8.2).

The waxy- or putty-like materials are similar to the waxy consistencies of the products of the replicated synthesis as discussed in Section "Precursor synthesis replication" (Fig. 4). In addition, the waxy materials seized from the English prisons as reported in Timmerman et al. 2024 were found to contain DMF, which is also consistent with the findings from the replicated synthesis. Therefore, these waxy- or putty-like materials are likely produced using the one-step synthesis method for converting tail-less precursors to the final desired SCRAs, although it is unknown if the production is occurring in the UK or elsewhere.

As shown in Table 3, of the 87 samples containing a mixture of a precursor with a corresponding final SCRA, MDMB-INACA was detected alongside MDMB-4en-PINACA in 75 samples, MDMB-4en-PINACA and MDMB-BUTINACA in 4 samples, MDMB-4en-PINACA and MDMB-FUBINACA in 2 samples, MDMB-FUBINACA in 2 samples, and MDMB-BUTINACA in 1 sample. Of these samples, 19 also contained ADB-BUTINACA, 1 sample ADB-4en-PINACA and ADB-5’Br-BUTINACA, and 1 sample ADB-4en-PINACA; however, the precursor ADB-INACA was not detected in any of these samples. The new tail-less SCRA MDMB-5’Me-INACA was also detected in nine of the MDMB-INACA-containing samples. Finally, AB-INACA was detected with AB-CHMINACA in three samples. Of the mixture between the precursor and final SCRA(s), the corresponding final SCRA(s) was present at between 1.9 and 99.9% of the sample with an average of 41.9%. On average, the purities of the Scottish samples (Table 3) were lower than those from the US samples (Table 2) although the reason for this is unknown. As seen with the precursor synthesis replication (Table 1), the synthesis method can produce a range of purities depending on a variety of factors, which may include the scale of the synthesis, recipe and limiting reagent, reaction conditions, purity of the starting materials, and technical expertise of the chemist.

**Table 3:** Tail-less precursors and final SCRA mixtures detected in 87 samples seized from the Scottish prisons and the percentage of the final SCRA in comparison to the corresponding precursor in the sample based on the percentage peak areas corrected for EI-MS detector response

Precursor	Final SCRA(s)	n	% Final SCRA vs precursor		
			Average	Min	Max
MDMB-INACA	MDMB-4en-PINACA	75	39.2	1.9	99.9
	MDMB-4en-PINACA + MDMB-BUTINACA	4	53.2	12.1	80.1
	MDMB-4en-PINACA + MDMB-FUBINACA	2	69.4	66.5	72.2
	MDMB-FUBINACA	2	68.8	67.8	69.7
	MDMB-BUTINACA	1	17.2	–	–
AB-INACA	AB-CHMINACA	3	66.0	50.0	81.8

#### Market evolution

As part of this study, four new tail-less SCRAs were detected for the first time: MDMB-ICA in the US in February 2022, MDMB-5’Me-INACA in the US in August 2023 and the Scottish prisons in September 2023, AB-INACA in the Scottish prisons in September 2023, and ADB-IATA in the US in December 2023. These have yet to be reported by the EU Early Warning System (EWS). In addition, MDMB-BUTINACA was detected for the first time in the US on 1st February 2023 and the Scottish prisons on 16th April 2023. MDMB-BUTINACA was first reported in the EU in May 2023 following detections in Turkey in October 2022, Bulgaria in December 2022, and Sweden in February 2023 (EMCDDA 2023).

**Fig. 5:**
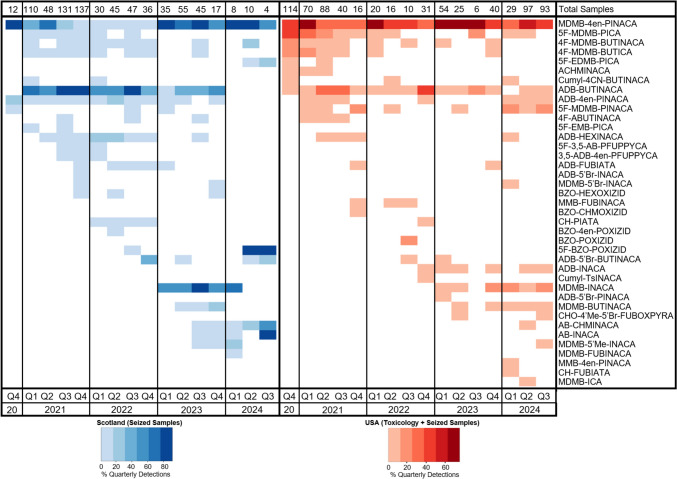
Comparison of the SCRA markets in the Scottish prisons from seized sample analysis and the US from toxicological and seized sample analysis based on data from the CSFRE’s NPS Discovery trend reports (CFSRE, n.d.)

While new SCRAs have continued to emerge, the re-emergence of SCRAs previously seen on the illicit market that disappeared following the enactment of legislative controls, including MDMB-4en-PINACA, 4F-MDMB-BUTINACA, MDMB-FUBINACA, and AB-CHMINACA, has also been observed. As can be seen in Fig. 5, MDMB-4en-PINACA was the most prevalent SCRA in the Scottish prisons between 2019 and 2021, but it mostly disappeared from the market following its legislative control in China in April 2021 (United Nations Office on Drugs and Crime (UNODC), 2021); however, from the beginning of 2023, it once again became the most prevalent SCRA on the market, but almost always in combination with its precursor MDMB-INACA. 

AB-CHMINACA was first detected in the EU in 2014 and by 2017 it had been detected by 24 Member States of the EU with 31 confirmed deaths (EMCDDA, 2018). MDMB-FUBINACA was first identified in October 2014 in Russia following an outbreak in multiple cities of severe intoxications, including some deaths (Kavanagh et al., 2017). Following the control of AB-CHMINACA and MDMB-FUBINACA by China in 2015 (UNODC, 2015), the US in 2017, and most of the EU by 2017 (EMCDDA, 2018), these compounds seemed to disappear from the recreational drugs market with the last reported detections in 2016 or 2017 based on data from the National Forensic Laboratory Information System (NFLIS) in the US, Siberian Federal District in Russia, and the Welsh Emerging Drugs and Identification of Novel Substances (WEDINOS) (NFLIS, n.d.; Oberenko et al., 2019; WEDINOS, 2019). Now, following their first detections in the Scottish prisons since the beginning of the project in 2018, AB-CHMINACA and MDMB-FUBINACA have re-emerged in September 2023 and February 2024, respectively. They were always detected in combination with their precursors AB-INACA and MDMB-INACA, respectively, indicating that these SCRAs are likely being synthesized from their tail-less precursors. So, while the SCRA analog ban in China at first led to a reduction in the potent SCRAs on the market, the new synthesis approach circumvents the ban as the precursors can be legally produced in China, supplied internationally, and then the synthesis can be completed close to areas where the drugs are distributed. This has resulted in the return of potent SCRAs to the illicit market that were previously indicated as contributing to many severe intoxications and deaths worldwide (EMCDDA, 2018, 2020; Kavanagh et al., 2017; Maeda et al., 2018).

### In vitro biological activity at CB_1_ receptor

**Fig. 6:**
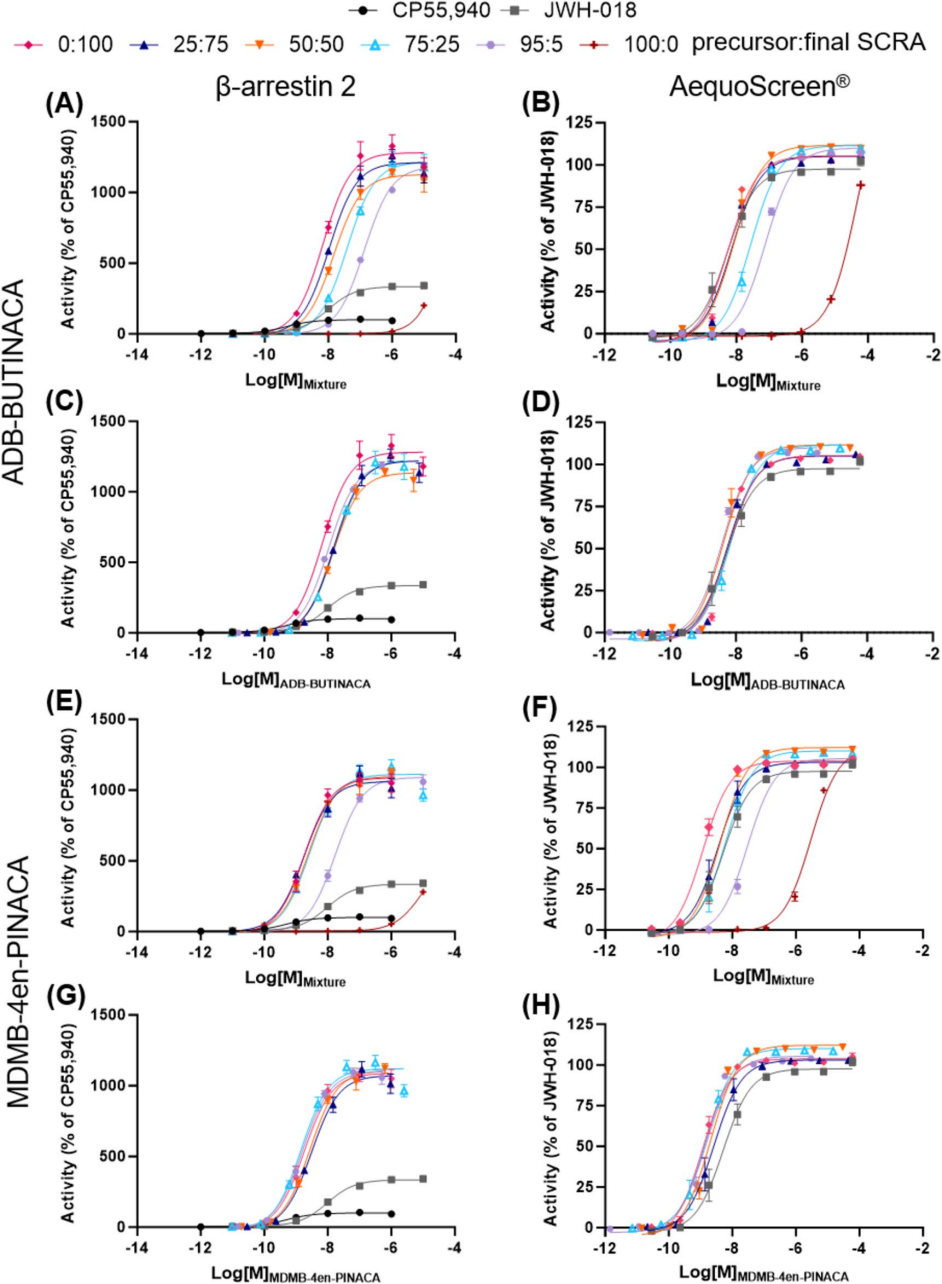
Concentration–response curves for the activation of the CB_1_ receptor by mixtures of precursors and final SCRAS (0:100, 25:75, 50:50, 75:25, 95:5, and 100:0 of ADB-INACA:ADB-BUTINACA and MDMB-INACA:MDMB-4en-PINACA). Data are presented in two ways: in (**a**), (**b**), (**e**), and (**f**), the total concentration of the SCRAs in the mixture (combined concentrations of the precursor and the final SCRA) is used and in (**c**), (**d**), (**g**), and (**h**), only the concentration of the final SCRA in the mixture is used. CP55,940 was used as a reference for the βarr2 assay and JWH-018 was used as a reference for the AequoScreen.® assay, but JWH-018 is included in the βarr2 assay for easier comparison between assays. Error bars indicate standard error to the mean (SEM)

In this study, two different in vitro CB_1_ receptor assays (the NanoBiT® β-arrestin 2 (βarr2) recruitment and AequoScreen® intracellular Ca^2+^ release assays) were used to examine the receptor activity of reference standards of the precursors and final products, including in mixtures, as often found in seized samples (Section "Seized sample analysis"), and the seized precursors and products of the replicated synthesis from Sweden. The products of the replicated synthesis from Norway were not tested due to their restrictions in shipping between countries. Figure 6 and Table 4 summarize the results for the assessment of mixtures or precursors with final SCRAs as well as comparison of the seized precursors with reference standards. Although different reference compounds were used for the assays, CP55,940 for the βarr2 assay and JWH-018 for the AequoScreen®, JWH-018 was also analyzed on the βarr2 assay and provided in the figures and tables for easier comparison. ADB-BUTINACA and MDMB-4en-PINACA are high potency SCRAs at the CB_1_ receptor with EC_50_ values of 6.7 and 1.8 nM, respectively, in the βarr2 assay and 5.6 and 1.2 nM, respectively, in the AequoScreen® assay. In comparison, the precursors showed very little activity with potencies that could not be calculated for ADB-INACA since the curves had not reached a plateau, and EC_50_ values of > 2000 nM for MDMB-INACA in both assays. These data are consistent between the two assays, as well as with previous reports of the CB_1_ receptor potency of these SCRAs (Antonides et al. 2019; Deventer et al. 2024; Kronstrand et al. 2022; Sparkes et al. 2022; Timmerman et al. 2024).

**Table 4:** Relative efficacy (E_max_) and potency (EC_50_) at the CB_1_ receptor calculated for the mixtures of precursors and final SCRAS (0:100, 25:75, 50:50, 75:25, 95:5, and 100:0 of ADB-INACA:ADB-BUTINACA and MDMB-INACA:MDMB-4en-PINACA). CP55,940 was used as a reference for the βarr2 assay and JWH-018 was used as a reference for the AequoScreen® assay, but JWH-018 is included in the βarr2 assay for easier comparison between assays

Compound/Mixture	β-arrestin 2				AequoScreen®			
	Potency (EC_50_, nM)		Efficacy (% of CP55,940)		Potency (EC_50_, nM)		Efficacy (% of JWH-018)	
	EC_50_	95% CI	E_max_	95% CI	EC_50_	95% CI	E_max_	95% CI
CP55,940	0.48	0.41–0.57	99.9	97.8–102	–	–	–	–
JWH-018	9.55	8.13–11.2	335	328–342	5.14	3.40–7.79	97.6	93–102
ADB-INACA: ADB-BUTINACA								
0:100 (ADB-BUTINACA)	6.73	4.35–10.3	1280	1210–1360	5.64	4.77–6.68	105	103–108
25:75	10.4	7.41–14.5	1210	1150–1270	7.14	6.13–8.32	105	103–107
50:50	14.6	10.9–19.8	1130	1080–1180	8.28	6.46–10.6	112	108–116
75:25	38.4	26.5–55.2	1220	1150–1290	27.6	23.3–32.8	116	109–114
95:5	132	116–151	1180	1160–1210	75.3	64.9–87.4	110	107–113
100:0 (ADB-INACA)	ND	ND	201^a^	–	ND	ND	159^b^	–
MDMB-INACA: MDMB-4en-PINACA								
0:100 (MDMB-4en-PINACA)	1.80	1.19–2.76	1090	1022–1155	1.17	0.957–1.44	104	102–106
25:75	1.75	1.11–2.85	1060	996–1130	3.50	2.42–5.14	103	98.6–108
50:50	2.39	1.76–3.25	1100	1050–1150	4.15	3.45–4.99	112	110–115
75:25	2.63	1.71–4.07	1110	1050–1170	6.09	4.54–8.15	110	106–114
95:5	17.1	13.6–21.7	1090	1050–1130	29.7	25.1–35.1	105	103–108
100:0 (MDMB-INACA)	ND	ND	280^a^	–	2960	2600–3370	112^b^	–

ND, not determined (values could not be calculated as saturation was not reached).

^a^Maximal activation observed at a concentration of 100 μM. Accompanying EC_50_ values could not be calculated accurately.

^b^Maximal activation observed at a concentration of 60 μM. Accompanying EC_50_ values could not be calculated accurately.

ADB-BUTINACA and MDMB-4en-PINACA also showed a high efficacy with E_max_ of 1282 and 1088% of CP55,940, respectively, for the βarr2 assay and 105 and 104% of JWH-018, respectively, for the AequoScreen® assay. ADB-INACA showed reduced efficacy of 672% of CP55,940 in the βarr2 assay and 56% of JWH-018, although since ADB-INACA did not reach a plateau in either assay, the efficacies are those obtained at the highest tested concentration (100 and 60 µM, respectively). MDMB-INACA also showed reduced efficacy in the βarr2 assay of 294% of CP55,940, but it showed similar efficacy to MDMB-4en-PINACA of 109% of JWH-018 in the AequoScreen® assay (based on the efficacy at the highest tested concentration of 60 µM). These differences in the efficacies between the two assays, where the βarr2 assay results in a wide range of efficacies and the AequoScreen® assay tends to result in a narrow range of efficacies clustering around the same maximum, have been discussed previously (Charlton and Vauquelin 2010; Deventer et al. 2024).

For the CB_1_ receptor activity of the 95:5, 75:25, 50:50, and 25:75 mixtures of ADB-INACA:ADB-BUTINACA and MDMB-INACA:MDMB-4en-PINACA, data were analyzed in two ways: using the total concentration of the SCRAs in the mixture (combined concentrations of the precursor and the final SCRA) and using only the concentration of the final SCRA in the mixture. The EC_50_ and E_max_ values reported in Table 4 are based on the combined concentrations, but concentration–response curves are provided from both analysis methods (Fig. 6). When analyzing the data using the total concentration of the SCRAs in the mixture (Fig. 6A, B, E, F), it is possible to see the activity expected from the use of these mixtures. For ADB-BUTINACA, only the 75:25 and 95:5 ADB-INACA:ADB-BUTINACA mixtures were found to be significantly different from the 100% ADB-BUTINACA (p < 0.05) in both assays. For MDMB-4en-PINACA, only the 95:5 MDMB-INACA:MDMB-4en-PINACA was found to be significantly different from the 100% MDMB-4en-PINACA (p < 0.05) in the β-arrestin 2 assay, whereas 95:5, 25:75, and 50:50 MDMB-INACA:MDMB-4en-PINACA were significantly different from the 100% MDMB-4en-PINACA in the AequoScreen® assay. The complete statistical results can be found in the Supplementary Information (Tables S10.1 and S10.3).

By compiling the concentration–response curves based on the concentration of the final SCRA (ADB-BUTINACA or MDMB-4en-PINACA), as shown in Fig. 6C, D, G, H, respectively, it is observed that the curves closely align and the EC_50_ and E_max_ values of the 5, 25, 50, and 75% final SCRA (ADB-BUTINACA or MDMB-4en-PINACA) were not found to be significantly different from each other or the 100% final SCRA (p > 0.05). On the other hand, the EC_50_ values from both assays and the E_max_ values for the βarr2 assay were significantly different from the 100% precursor (MDMB-INACA) (p < 0.05), but ADB-INACA could not be statistically compared since it did not reach a plateau. The complete statistical results can be found in the Supplementary Information (Tables S10.2 and S10.4). Even the most extreme mixture of 95:5 precursor:final SCRA, which means that the molarity of the precursor in the system is 19 times higher than that of the precursor, still did not show any significant change in the activity at CB_1_ of the final SCRA. These results indicate that the presence of precursors has no significant influence on the activity of the final SCRA at CB_1_. Therefore, the activity of mixtures of the precursors and final SCRAs, as often found in seized samples (Section "Seized sample analysis"), is solely dictated by the concentration of the final SCRA. Although the tail-less precursors do not have a significant influence on the CB_1_ activity, their presence in a mixture may influence metabolism or interfere with other pharmacological targets. For example, one study found both ADB-INACA and MDMB-INACA showed analgesic effects on paclitaxel-induced peripheral neuropathy in rats, indicating the tail-less precursors may still exert pharmacologically relevant effects on a person using them.

**Fig. 7:**
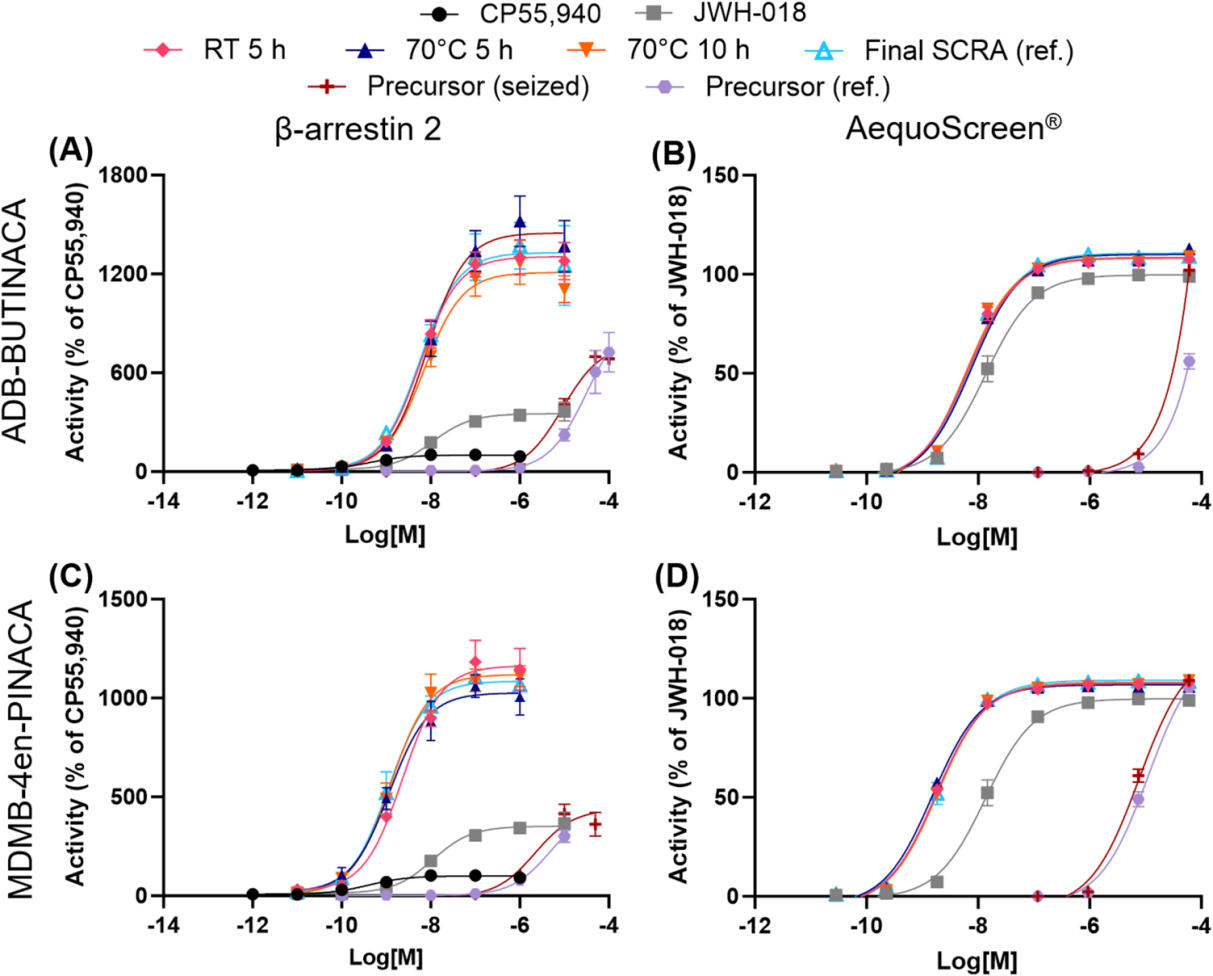
Concentration–response curves for the activation of the CB_1_ receptor by precursors seized from Switzerland and the products of the replicated synthesis from the precursors from Sweden: **a** and **b** for ADB-INACA and ADB-BUTINACA and **c** and **d** for MDMB-INACA and MDMB-4en-PINACA. The products of the replicated synthesis from Norway were not tested due to restrictions in their shipment between countries. CP55,940 was used as a reference for the βarr2 assay and JWH-018 was used as a reference for the AequoScreen® assay, but JWH-018 is included in the βarr2 assay for easier comparison between assays. Error bars indicate standard error to the mean (SEM)

The CB_1_ receptor activity of the seized precursors and products of the replicated synthesis from Sweden was also examined using both in vitro assays and the results can be found in Table 5 and Fig. 7. The products of the replicated synthesis from Norway were not tested due to restrictions in their shipment between countries. The seizures of the precursors ADB-INACA and MDMB-INACA from the clandestine laboratory in Switzerland (Section "Clandestine laboratory investigation") had a similar potency and efficacy as the reference standards in both assays (p > 0.05 for MDMB-INACA; statistical analysis not possible for ADB-INACA since it did not reach a plateau). This indicates the seized samples had high purities and suggests the absence of interfering constituents, which is consistent with the GC–FID–MS and HPLC analyses conducted on them (Section "Clandestine laboratory investigation").

**Table 5:** Relative efficacy (E_max_) and potency (EC_50_) at the CB_1_ receptor calculated for precursors seized from Switzerland and the products of the replicated synthesis from the precursor from Sweden

Compound	Conditions	β-arrestin 2				AequoScreen®			
		Potency (EC_50_, nM)		Efficacy (% of CP55,940)		Potency (EC_50_, nM)		Efficacy (% of JWH-018)	
		EC_50_	95% CI	E_max_	95% CI	EC_50_	95% CI	E_max_	95% CI
CP55,940	Ref	0.404	0.175–0.885	100	91.6–110	–	–	–	–
JWH-018	Ref	11.0	5.31–23.4	352	318–387	13.5	10.9–16.6	99.8	97.0–103
ADB-INACA	Ref	ND	ND	672^a^	–	ND	ND	56.1^b^	–
	Seizure	ND	ND	684^a^	–	ND	ND	102^b^	–
MDMB-INACA	Ref	2400	1500–3850	294	264–325	11,200	8990–14100	109^b^	-
	Seizure	2190	1300–3710	438	385–495	7610	6190–9380	105^b^	-
ADB-BUTINACA	Ref	5.65	2.35–12.7	1330	1190–1470	7.27	6.29–8.40	111	109–113
	RT 5 h	5.71	3.45–9.25	1310	1220–1390	6.69	5.68–7.88	108	106–111
	70 °C 5 h	8.13	4.25–15.3	1450	1320–1580	7.68	6.48–9.10	110	108–113
	70 °C 10 h	6.87	3.65–12.5	1210	1110–1310	6.14	5.13–7.37	109	106–111
MDMB-4en-PINACA	Ref	1.80	1.19–2.76	1090	1020–1160	1.89	1.59–2.25	109	107–111
	RT 5 h	1.75	1.11–2.85	1060	996–1130	1.74	1.56–1.96	108	106–109
	70 °C 5 h	2.39	1.76–3.25	1100	1050–1150	1.53	1.32–1.78	107	105–109
	70 °C 10 h	2.63	1.71–4.07	1110	1050–1170	1.84	1.58–2.15	108	106–110

The products of the replicated synthesis from Norway were not tested due to restrictions in their shipment between countries. CP55,940 was used as a reference for the βarr2assay and JWH-018 was used as a reference for the AequoScreen® assay, but JWH-018 is included in the βarr2 assay for easier comparison between assays

ND, not determined (values could not be calculated as saturation was not reached)

^a^Maximal activation observed at a concentration of 100 μM. Accompanying EC_50_ values could not be calculated accurately

^b^Maximal activation observed at a concentration of 60 μM. Accompanying EC_50_ values could not be calculated accurately

In addition, no significant differences were observed between the EC_50_ and E_max_ values of the products from the replicated synthesis under different reaction conditions from Sweden and the reference standard of the final product (ADB-BUTINACA or MDMB-4en-PINACA) for both assays (p > 0.05). The complete statistical results can be found in the Supplementary Information (Tables S8.3 and S8.4). This indicates that the products’ differences in purity, with one being as low as 75.5%, did not have a significant effect on the activity. This is consistent with the results of the in vitro receptor activity of the reference standard mixtures, where the 25:75 precursor:final SCRA mixtures were not found to be significantly different from 100% final SCRA.

## Conclusion

This study highlights the use of a new production method for SCRAs from tail-less precursors using a one-step synthesis that has emerged since the 2021 Chinese SCRA ban. The synthesis procedures described online were found to be comparable to those used at a clandestine laboratory discovered in Switzerland. The synthesis reproduced by two independent laboratories using different reaction conditions resulted in varying product characteristics of the final product. Notably, the MDMB-4en-PINACA synthesis resulted in crude oils, while the ADB-BUTINACA synthesis resulted in powdery and waxy materials.

This production route is being used in multiple countries around the world as demonstrated by samples seized between February 2023 and July 2024 from the US and Scottish prisons that contain mixtures of tail-less precursors and corresponding final SCRAs. It is likely as a result of this new production method that SCRAs controlled by the 2021 Chinese SCRA class-wide ban continue to be detected and some formerly prevalent SCRAs have re-emerged, such as AB-CHMINACA and MDMB-FUBINACA. The biological activity at the CB_1_ receptor of mixtures of the precursors and final SCRAs indicates that potency and efficacy are solely determined by the concentration of the final SCRA present. Further monitoring of this trend is advised as the continuing use of this synthesis route is anticipated and will likely result in increased domestic SCRA production in future.

## Supplementary Information

Below is the link to the electronic supplementary material.

Supplementary file1 (DOCX 32218 KB)

## Data Availability

The authors confirm that the data supporting the findings of this study are available within the article [and/or] its supplementary materials.

## References

[CR1] Andrews R, Jorge R, Christie R, Gallegos A (2023) From JWH-018 to OXIZIDS: structural evolution of synthetic cannabinoids in the European Union from 2008 to present day. Drug Test Anal 15(4):378–387. 10.1002/dta.3422 36507715 10.1002/dta.3422

[CR2] Angerer V, Jacobi S, Franz F, Auwärter V, Pietsch J (2017) Three fatalities associated with the synthetic cannabinoids 5F-ADB, 5F-PB-22, and AB-CHMINACA. Forensic Sci Int 281:e9–e15. 10.1016/j.forsciint.2017.10.042 29133010 10.1016/j.forsciint.2017.10.042

[CR3] Antonides LH, Cannaert A, Norman C, Vives L, Harrison A, Costello A, Nic Daeid N, Stove CP, Sutcliffe OB, McKenzie C (2019) Enantiospecific synthesis, chiral separation, and biological activity of four indazole-3-carboxamide-type synthetic cannabinoid receptor agonists and their detection in seized drug samples. Front Chem. 10.3389/fchem.2019.00321 31157203 10.3389/fchem.2019.00321 PMC6532652

[CR4] Antonides LH, Cannaert A, Norman C, Nic Daéid N, Sutcliffe OB, Stove CP, McKenzie C (2021) Shape matters: the application of activity-based in vitro bioassays and chiral profiling to the pharmacological evaluation of synthetic cannabinoid receptor agonists in drug-infused papers seized in prisons. Drug Test Anal 13(3):628–643. 10.1002/dta.2965 33161649 10.1002/dta.2965

[CR5] Åstrand A, Guerrieri D, Vikingsson S, Kronstrand R, Green H (2020) In vitro characterization of new psychoactive substances at the μ-opioid, CB1, 5HT1A, and 5-HT2A receptors—On-target receptor potency and efficacy, and off-target effects. Forensic Sci Int 317(110553):1–12. 10.1016/j.forsciint.2020.110553 10.1016/j.forsciint.2020.110553 33160102

[CR6] Boland DM, Reidy LJ, Seither JM, Radtke JM, Lew EO (2020) Forty-three fatalities involving the synthetic cannabinoid, 5-fluoro-adb: forensic pathology and toxicology implications. J Forensic Sci 65(1):170–182. 10.1111/1556-4029.14098 31211877 10.1111/1556-4029.14098

[CR7] Cannaert A, Storme J, Franz F, Auwärter V, Stove CP (2016) Detection and activity profiling of synthetic cannabinoids and their metabolites with a newly developed bioassay. Anal Chem 88:11476–11485. 10.1021/acs.analchem.6b02600 27779402 10.1021/acs.analchem.6b02600

[CR8] Cannaert A, Franz F, Auwärter V, Stove CP (2017) Activity-based detection of consumption of synthetic cannabinoids in authentic urine samples using a stable cannabinoid reporter system. Anal Chem 89:9527–9536. 10.1021/acs.analchem.7b02552 28771321 10.1021/acs.analchem.7b02552

[CR9] Cannaert A, Storme J, Hess C, Auwärter V, Wille SMR, Stove CP (2018) Activity-based detection of cannabinoids in serum and plasma samples. Clin Chem 64(6):918–926. 10.1373/clinchem.2017.285361 29559524 10.1373/clinchem.2017.285361

[CR10] Cannaert A, Sparkes E, Pike E, Luo JL, Fang A, Kevin RC, Ellison R, Gerona R, Banister SD, Stove CP (2020) Synthesis and in vitro cannabinoid receptor 1 activity of recently detected synthetic cannabinoids 4F-MDMB-BICA, 5F-MPP-PICA, MMB-4en-PICA, CUMYL-CBMICA, ADB-BINACA, APP-BINACA, 4F-MDMB-BINACA, MDMB-4en-PINACA, A-CHMINACA, 5F-AB-P7AICA, 5F-MDMB-P7AICA, an. ACS Chem Neurosci 11(24):4434–4446. 10.1021/acschemneuro.0c00644 33253529 10.1021/acschemneuro.0c00644

[CR11] The Center for Forensic Science Research and Education (CFSRE) (n.d.) NPS Discovery Trend Reports. https://www.cfsre.org/nps-discovery/trend-reports/synthetic-cannabinoids/report/49?trend_type_id=4. Accessed 08 Sep 2024.

[CR12] Charlton SJ, Vauquelin G (2010) Elusive equilibrium: the challenge of interpreting receptor pharmacology using calcium assays. Br J Pharmacol 161(6):1250–1265. 10.1111/j.1476-5381.2010.00863.x 20977466 10.1111/j.1476-5381.2010.00863.x PMC3000651

[CR13] Deventer MH, Van Uytfanghe K, Vinckier IMJ, Reniero F, Guillou C, Stove CP (2022a) A new cannabinoid receptor 1 selective agonist evading the 2021 “China ban”: ADB-FUBIATA. Drug Test Anal 14(9):1639–1644. 10.1002/dta.3285 35570246 10.1002/dta.3285

[CR14] Deventer MH, Van Uytfanghe K, Vinckier IMJ, Reniero F, Guillou C, Stove CP (2022b) Cannabinoid receptor activation potential of the next generation, generic ban evading OXIZID synthetic cannabinoid receptor agonists. Drug Test Anal 14(9):1565–1575. 10.1002/dta.3283 35560866 10.1002/dta.3283

[CR15] Deventer MH, Norman C, Reid R, McKenzie C, Nic Daéid N, Stove CP (2023) In vitro characterization of the pyrazole-carrying synthetic cannabinoid receptor agonist 5F–3,5-AB-PFUPPYCA and its structural analogs. Forensic Sci Int 343(111565):1–7. 10.1016/j.forsciint.2023.111565 10.1016/j.forsciint.2023.111565 36640535

[CR16] Deventer MH, Persson M, Norman C, Liu H, Connolly M, Nic Daéid N, McKenzie C, Gréen H, Stove CP (2024) In vitro cannabinoid activity profiling of generic ban-evading brominated synthetic cannabinoid receptor agonists and their analogs. Drug Test Anal 16(6):1–13. 10.1002/dta.3592 10.1002/dta.3592 37903509

[CR17] European Monitoring Centre for Drugs and Drug Addiction (EMCDDA) (2017) Synthetic cannabinoids in Europe. http://www.emcdda.europa.eu/topics/pods/synthetic-cannabinoids_en#panel3

[CR18] European Monitoring Centre for Drugs and Drug Addiction (EMCDDA) (2018) Report on the risk assessment of N-(1-amino-3-methyl-1-oxobutan-2-yl)-1-(cyclohexylmethyl)-1H-indazole-3-carboxamide (AB-CHMINACA) in the framework of the Council Decision on new psychoactive substances. 10.2810/565855

[CR19] European Monitoring Centre for Drugs and Drug Addiction (EMCDDA) (2020) EMCDDA initial report on the new psychoactive substance methyl 3,3-dimethyl-2-(1-(pent-4-en-1-yl)-1H-indazole-3-carboxamido)butanoate (MDMB-4en-PINACA). https://www.emcdda.europa.eu/system/files/publications/13363/emcdda-initial-report-MDMB-4en-PINACA.pdf

[CR20] European Monitoring Centre for Drugs and Drug Addiction (EMCDDA) (2023) EU Early Warning System Formal Notification. [Notification of methyl 2-(1-butyl-1H-indazole-3-carboxamido)-3,3-dimethylbutanoate (MDMB-BINACA) in Europe.] EU-EWS-RCS-FN-2023-0013.

[CR21] European Monitoring Centre for Drugs and Drug Addiction (EMCDDA) (2024) European Drug Report 2024: Trends and Developments. 10.2810/91693

[CR22] European Union Drugs Agency & Europol (2024) EU Drug Market: New psychoactive substances—In-depth analysis. 10.2810/338463

[CR23] Grafinger KE, Cannaert A, Ametovski A, Sparkes E, Cairns E, Banister SD, Auwärter V, Stove CP (2021) Systematic evaluation of a panel of 30 synthetic cannabinoid receptor agonists structurally related to MMB-4en-PICA, MDMB-4en-PINACA, ADB-4en-PINACA, and MMB-4CN-BUTINACA using a combination of binding and different CB1 receptor activation assays—Part II. Drug Test Anal 13(7):1402–1411. 10.1002/dta.3035 33769699 10.1002/dta.3035

[CR24] Groth O, Roider G, Angerer V, Schäper J, Graw M, Musshoff F, Auwärter V (2023) “Spice”-related deaths in and around Munich, Germany: a retrospective look at the role of synthetic cannabinoid receptor agonists in our post-mortem cases over a seven-year period (2014–2020). Int J Legal Med 137(4):1059–1069. 10.1007/s00414-023-02995-2 37072496 10.1007/s00414-023-02995-2 PMC10247575

[CR25] Kavanagh P, Grigoryev A, Krupina N (2017) Detection of metabolites of two synthetic cannabimimetics, MDMB-FUBINACA and ADB-FUBINACA, in authentic human urine specimens by accurate mass LC–MS: a comparison of intersecting metabolic patterns. Forensic Toxicol 35(2):284–300. 10.1007/s11419-017-0356-y

[CR26] Kim TH, Kim SG (2011) Clinical outcomes of occupational exposure to N, N-dimethylformamide: perspectives from experimental toxicology. Saf Health Work 2(2):97–104. 10.5491/SHAW.2011.2.2.97 22953193 10.5491/SHAW.2011.2.2.97 PMC3431905

[CR27] Kronstrand R, Norman C, Vikingsson S, Biemans A, Crespo BV, Edwards D, Fletcher D, Gilbert N, Persson M, Reid R, Semenova O, Al Teneiji F, Wu X, Dahlén J, Nic Daéid N, Tarbah F, Sutcliffe OB, McKenzie C, Gréen H (2022) The metabolism of the synthetic cannabinoids ADB-BUTINACA and ADB-4en-PINACA and their detection in forensic toxicology casework and infused papers seized in prisons. Drug Test Anal 14(4):634–652. 10.1002/dta.3203 34811926 10.1002/dta.3203

[CR28] Labay LM, Caruso JL, Gilson TP, Phipps RJ, Knight LD, Lemos NP, McIntyre IM, Stoppacher R, Tormos LM, Wiens AL (2016) Synthetic cannabinoid drug use as a cause or contributory cause of death. Forensic Sci Int 260:31–39. 10.1016/j.forsciint.2015.12.046 26795398 10.1016/j.forsciint.2015.12.046

[CR29] Liu C-M, Hua Z-D, Jia W, Li T (2022) Identification of AD-18, 5F-MDA-19, and pentyl MDA-19 in seized materials after the class-wide ban of synthetic cannabinoids in China. Drug Test Anal 14(2):307–316. 10.1002/dta.3185 34694738 10.1002/dta.3185

[CR30] Luethi D, Liechti ME (2020) Designer drugs: mechanism of action and adverse effects. In: Archives of toxicology (vol. 94, issue 4). Springer, Berlin. 10.1007/s00204-020-02693-7 10.1007/s00204-020-02693-7 PMC7225206 32249347

[CR31] Maeda H, Kikura-Hanajiri R, Kawamura M, Nagashima E, Yoshida KI (2018) AB-CHMINACA-induced sudden death from non-cardiogenic pulmonary edema. Clin Toxicol 56(2):143–145. 10.1080/15563650.2017.1340648 10.1080/15563650.2017.1340648 28707493

[CR32] Marland V, Reid R, Brandon AM, Hill K, Cruickshanks F, McKenzie C, Norman C, Nic Daéid N, Ménard H (2024) Changing trends in novel benzodiazepine use within Scottish prisons: detection, quantitation, prevalence, and modes of use. Drug Test Anal 16(5):457–472. 10.1002/dta.3560 37587559 10.1002/dta.3560

[CR33] Monti MC, Zeugin J, Koch K, Milenkovic N, Scheurer E, Mercer-Chalmers-Bender K (2022) Adulteration of low-delta-9-tetrahydrocannabinol products with synthetic cannabinoids: results from drug checking services. Drug Test Anal 14(6):1026–1039. 10.1002/dta.3220 34997693 10.1002/dta.3220 PMC9305195

[CR34] National Forensic Laboratory Information System (NFLIS). (n.d.). NFLIS Published Reports. Retrieved August 26, 2020, from https://www.nflis.deadiversion.usdoj.gov/Reports.aspx

[CR35] Norman C, Walker G, McKirdy B, McDonald C, Fletcher D, Antonides LH, Sutcliffe OB, Nic Daéid N, McKenzie C (2020) Detection and quantitation of synthetic cannabinoid receptor agonists in infused papers from prisons in a constantly evolving illicit market. Drug Test Anal 12(4):538–554. 10.1002/dta.2767 31944624 10.1002/dta.2767

[CR36] Norman C, Halter S, Haschimi B, Acreman D, Smith J, Krotulski AJ, Mohr ALA, Logan BK, NicDaéid N, Auwärter V, McKenzie C (2021) A transnational perspective on the evolution of the synthetic cannabinoid receptor agonists market: comparing prison and general populations. Drug Test Anal 13(4):841–852. 10.1002/dta.3002 33463894 10.1002/dta.3002

[CR37] Norman C, Webling K, Kyslychenko O, Reid R, Krotulski AJ, Farrell R, Deventer MH, Liu H, Connolly MJ, Guillou C, Vinckier IM, Logan BK, Nic Daéid N, McKenzie C, Stove CP, Gréen H (2024) Detection in seized samples, analytical characterization, and in vitro metabolism of the newly emerged 5-bromo-indazole-3-carboxamide synthetic cannabinoid receptor agonists. Drug Test Anal 16(9):915–935. 10.1002/dta.3609 38037247 10.1002/dta.3609

[CR38] Oberenko AV, Kachin SV, Sagalakov S (2019) Types of synthetic cannabinoids seized from illicit trafficking in the territory of the Siberian Federal District (Russia) between 2009–2018. Forensic Sci Int 302(109902):1–5. 10.1016/j.forsciint.2019.109902 10.1016/j.forsciint.2019.109902 31382224

[CR39] Oomen PE, Schori D, Tögel-Lins K, Acreman D, Chenorhokian S, Luf A, Karden A, Paulos C, Fornero E, Gerace E, Koning RPJ, Galindo L, Smit-Rigter LA, Measham F, Ventura M (2022) Cannabis adulterated with the synthetic cannabinoid receptor agonist MDMB-4en-PINACA and the role of European drug checking services. Int J Drug Policy 100(103493):1–5. 10.1016/j.drugpo.2021.103493 10.1016/j.drugpo.2021.103493 34687992

[CR40] Patel M, Zheng X, Akinfiresoye LR, Prioleau C, Walker TD, Glass M, Marusich JA (2024) Pharmacological evaluation of new generation OXIZID synthetic cannabinoid receptor agonists. Eur J Pharmacol 971:176549. 10.1016/j.ejphar.2024.176549 38561104 10.1016/j.ejphar.2024.176549 PMC11132922

[CR41] Pike E, Grafinger KE, Cannaert A, Ametovski A, Luo JL, Sparkes E, Cairns EA, Ellison R, Gerona R, Stove CP, Auwärter V, Banister SD (2021) Systematic evaluation of a panel of 30 synthetic cannabinoid receptor agonists structurally related to MMB-4en-PICA, MDMB-4en-PINACA, ADB-4en-PINACA, and MMB-4CN-BUTINACA using a combination of binding and different CB1 receptor activation assays: Part I—Synthesis, analytical characterization, and binding affinity for human CB1 receptors. Drug Test Anal 13(7):1383–1401. 10.1002/dta.3037 33787091 10.1002/dta.3037

[CR42] Pulver B, Fischmann S, Westphal F, Schönberger T, Schäper J, Budach D, Jacobsen-Bauer A, Dreiseitel W, Zagermann J, Damm A, Knecht S, Opatz T, Auwärter V, Pütz M (2022a) The ADEBAR project: European and international provision of analytical data from structure elucidation and analytical characterization of NPS. Drug Test Anal 14(8):1491–1502. 10.1002/dta.3280 35524160 10.1002/dta.3280

[CR43] Pulver B, Schönberger T, Weigel D, Köck M, Eschenlohr Y, Lucas T, Podlesnik N, Opatz T, Dreiseitel W, Pütz M, Schäper J, Jacobsen-Bauer A, Auwärter V, Westphal F (2022b) Structure elucidation of the novel synthetic cannabinoid Cumyl-Tosyl-Indazole-3-Carboxamide (Cumyl-TsINACA) found in illicit products in Germany. Drug Test Anal 14(8):1387–1406. 10.1002/dta.3261 35338591 10.1002/dta.3261

[CR44] Pulver B, Riedel J, Schönberger T, Halter S, Lucas T, Opatz T, Grafinger KE, Scheu M, Zschiesche A, Pütz M, Pützer K, Westphal F, Auwärter V (2023) Pharmacology, prevalence in Germany, and analytical data of cyclobutylmethyl- and norbornylmethyl-type synthetic cannabinoid receptor agonists. Drug Test Anal 15(4):408–425. 10.1002/dta.3427 36541839 10.1002/dta.3427

[CR45] Rautio T, Connolly M, Liu H, Konradsson P, Gréen H, Dahlén J, Wu X (2024) Direct and selective alkylation of indazole-3-carboxylic acid for the preparation of synthetic cannabinoids and their metabolites. Forensic Chem 40(100603):1–9. 10.1016/j.forc.2024.100603

[CR46] Rautio T, Obrist R, Krebs L, Klingstedt T, Dahlén J, Wu X, Gréen H (2025) In vitro metabolism study of ADB-P-5Br-INACA and ADB-4en-P-5Br-INACA using human hepatocytes, liver microsomes, and in-house synthesized references. Drug Test Anal 17(5):701-712. 10.1002/dta.3773 39039949 10.1002/dta.3773 PMC12012409

[CR47] Shafi A, Berry AJ, Sumnall H, Wood DM, Tracy DK (2020) New psychoactive substances: a review and updates. Therapeutic Adv Psychopharmacol 10:1–21. 10.1177/2045125320967197 10.1177/2045125320967197 PMC7750892 33414905

[CR48] Sparkes E, Cairns EA, Kevin RC, Lai F, Grafinger KE, Chen S, Deventer MH, Ellison R, Boyd R, Matin LJ, McGregor IS, Gerona RR, Hibbs DE, Auwärter V, Glass M, Stove C, Banister SD (2022) Structure–activity relationships of valine, tert-leucine, and phenylalanine amino acid-derived synthetic cannabinoid receptor agonists related to ADB-BUTINACA, APP-BUTINACA, and ADB-P7AICA. RSC Med Chem 13:156–174. 10.1039/D1MD00242B 35308023 10.1039/d1md00242b PMC8864554

[CR49] Sparkes E, Timmerman A, Markham JW, Boyd R, Gordon R, Walker KA, Kevin RC, Hibbs DE, Banister SD, Cairns EA, Stove C, Ametovski A (2024) Synthesis and functional evaluation of synthetic cannabinoid receptor agonists related to ADB-HEXINACA. ACS Chem Neurosci 15(9):1787–1812. 10.1021/acschemneuro.3c00818 38597712 10.1021/acschemneuro.3c00818

[CR50] Timmerman A, Deventer MH, Andrews R, Reid R, Marland V, Edwards D, Pudney CR, Nic Daéid N, Stove CP, Norman C (2024) Waxy- or putty-like materials as a novel drug preparation for synthetic cannabinoid receptor agonists: detection in prisons and in vitro cannabinoid receptor activity. Drug Test Anal. 10.1002/dta.3817 39410765 10.1002/dta.3817 PMC12209699

[CR51] Truver MT, Watanabe S, Åstrand A, Vikingsson S, Green H, Swortwood MJ, Kronstrand R (2020) 5F-MDMB-PICA metabolite identification and cannabinoid receptor activity. Drug Test Anal 12(1):127–135. 10.1002/dta.2688 31461219 10.1002/dta.2688

[CR52] UNODC (2015) October 2015—China: China announces controls over 116 New Psychoactive Substances. https://www.unodc.org/LSS/Announcement/Details/83b02e73-4896-4ed5-944c-51a7646647aa

[CR53] United Nations Office on Drugs and Crime (UNODC). (2021). April 2021—UNODC: Eight substances “scheduled” at the 64th Session of the Commission on Narcotic Drugs. https://www.unodc.org/unodc/en/scientists/ewa/news/2021/april/03.html

[CR54] Welsh Emerging Drugs and Identification of Novel Substances. (2019). Sample Results. https://www.wedinos.org/db/samples/

[CR55] Wouters E, Mogler L, Cannaert A, Auwärter V, Stove C (2019) Functional evaluation of carboxy metabolites of synthetic cannabinoid receptor agonists featuring scaffolds based on L-valine or L-tert-leucine. Drug Test Anal 11(8):1183–1191. 10.1002/dta.2607 31021521 10.1002/dta.2607

[CR56] 狄斌, 陈小意, & 马潇 (2024) Hapten for synthesizing cannabinoid ADB-BUTINACA, monoclonal antibody and application thereof (CN118063385A).

