# Supplementary material for: Tail-less precursors in synthetic cannabinoid production: investigating a clandestine laboratory, seized samples, and CB1 activity
Source: Arch Toxicol. 2025 May 23;99(9):3641–61. doi: 10.1007/s00204-025-04086-0 (PMC12408681; doi:10.1007/s00204-025-04086-0)
Supplement: Supplementary file 1 — Supplementary file1 (DOCX 32218 KB) [file 204_2025_4086_MOESM1_ESM.docx]

**Tail-Less Precursors in Synthetic Cannabinoid Production: Investigating a Clandestine Laboratory, Seized Samples, and CB_1_ Activity**

Manuela Carla Monti^1^, Tobias Rautio^2^, Marie H. Deventer^3^, Markus Schläpfer^4^, Johannes Tveit^5^, Alex J. Krotulski^6^, Victoria Marland^7^, Robert Reid^7^, Niamh Nic Daeid^7^, Craig McKenzie^5,7^, Christophe P. Stove^3^, Henrik Green^1,8^, Caitlyn Norman^1,*^

^1^Division of Clinical Chemistry and Pharmacology, Department of Biomedical and Clinical Sciences, Linköping University, Linköping, Sweden

^2^Department of Physics, Chemistry and Biology, Linköping University, Linköping, Sweden

^3^Laboratory of Toxicology, Department of Bioanalysis, Faculty of Pharmaceutical Sciences, Ghent University, Ghent, Belgium

^4^Zurich Forensic Science Institute, Zurich, Switzerland

^5^Chiron AS, Trondheim, Norway

^6^Center for Forensic Science Research and Education, Fredric Rieders Family Foundation, Willow Grove, Pennsylvania, USA

^7^Leverhulme Research Centre for Forensic Science, School of Science and Engineering, University of Dundee, Dundee, UK

^8^Department of Forensic Genetics and Forensic Toxicology, National Board of Forensic Medicine, Linköping, Sweden

^*^Corresponding author: caitlyn.norman@liu.se

**Supplementary Information**

**SECTION 1:** Screenshots from internet vendors selling “semi-finished” kits.

**SECTION 2:** Analytical characterization of synthesized SCRA reference standards

**SECTION 3:** Corrections for EI-MS detector response for the percentage peak area of SCRAs in seized samples.

**SECTION 4:** Analysis results of items seized from the clandestine laboratory and related sites.

**SECTION 5:** Analytical data for the products of the synthesis from the precursors replicated at two independent laboratories (one in Sweden and one in Norway) employing different synthesis durations (5 and 10 h) and temperatures (room temperature (RT) and 70˚C).

**SECTION 6:** Complete data for the US seized samples found positive for a tail-less SCRA/precursor

**SECTION 7:** Example examination photographs of samples seized from the Scottish prisons.

**SECTION 8:** Complete data for the samples seized from the Scottish prisons found positive for a tail-less SCRA/precursor with one or two corresponding SCRA(s).

**SECTION 9:** Complete datasets used for the creation of the heat map.

**SECTION 10:** Complete statistical analysis results for the *in vitro* CB_1_ receptor activity.

**SECTION 1**

Screenshots from webpages of precursor SCRAs vendors.


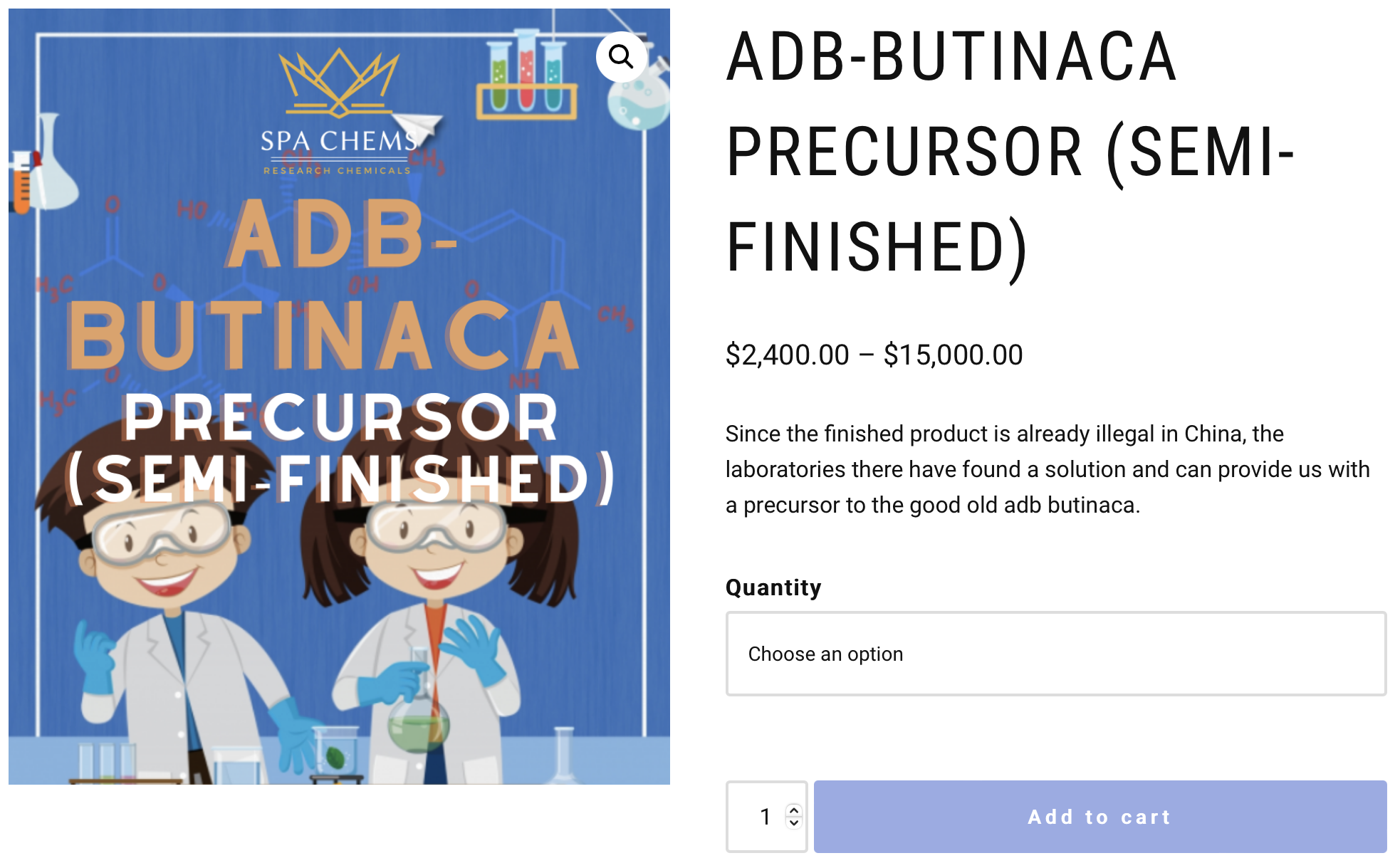


**Figure S1.1.** Screenshot from a vendor website selling ADB-BUTINACA precursor (semi-finished) and stating the legality of the precursors in China compared to the finished products.

**
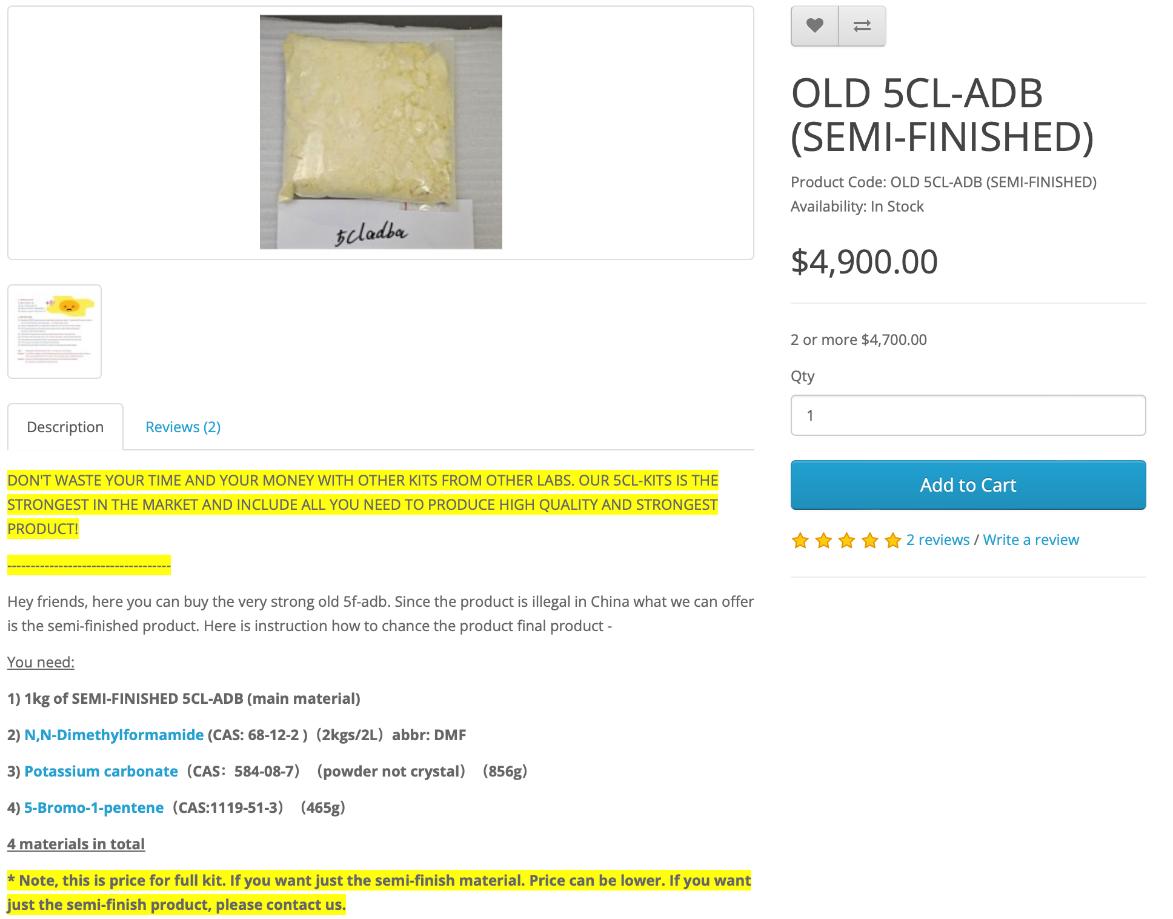
**

**Figure S1.2.** Screenshot of an online advertisement of “semi-finished” kits, which include the precursor and further chemicals and reagents required for the synthesis. 5CL-ADB is a name used on vendor sites for MDMB-4en-PINACA, which is also supported by the inclusion of 5-bromo-1-pentene in the kit.


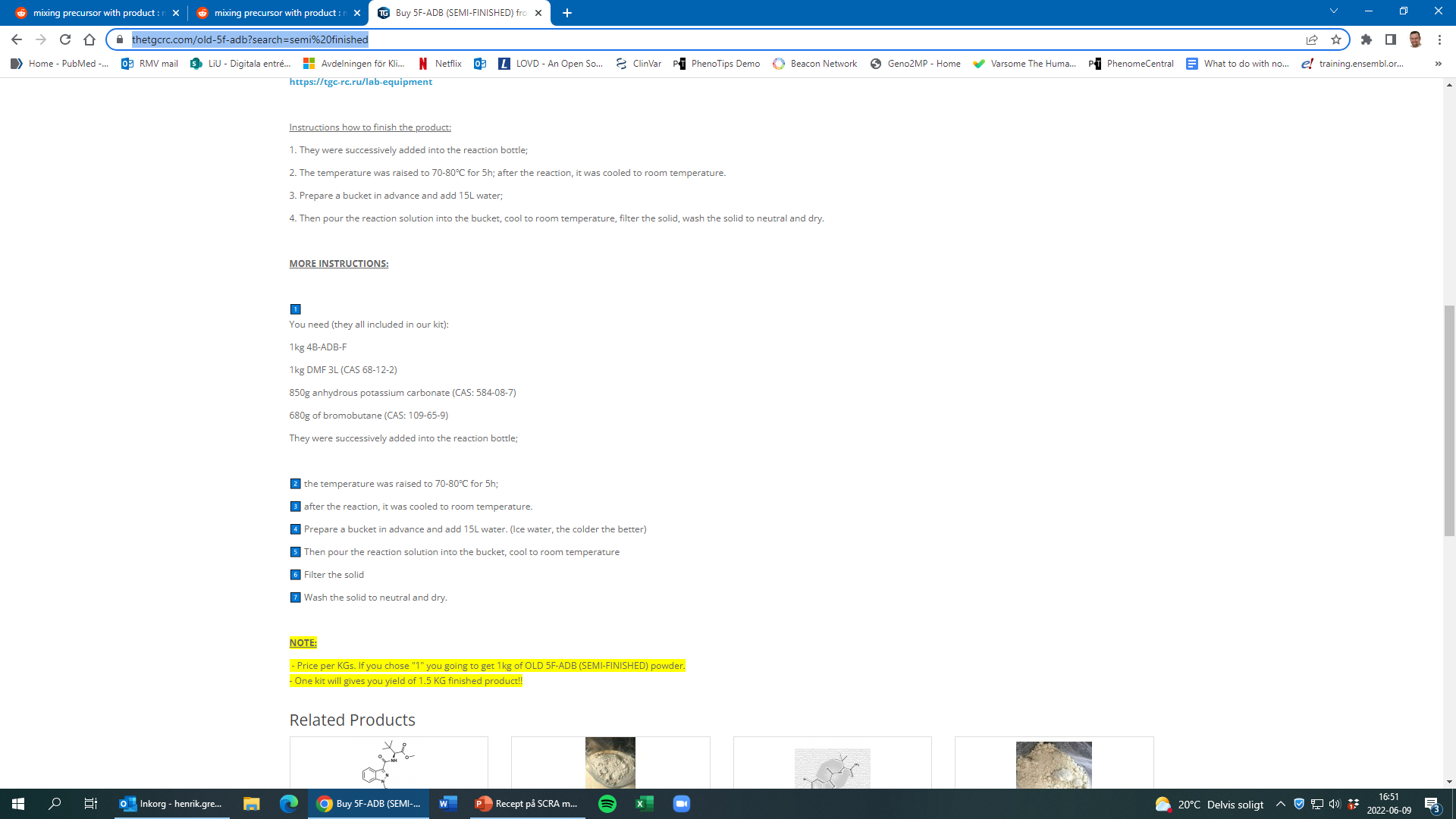


**Figure S1.3.** Screenshot of step-by-step instructions for the synthesis of a SCRA from a precursor published on a vendor site.

**
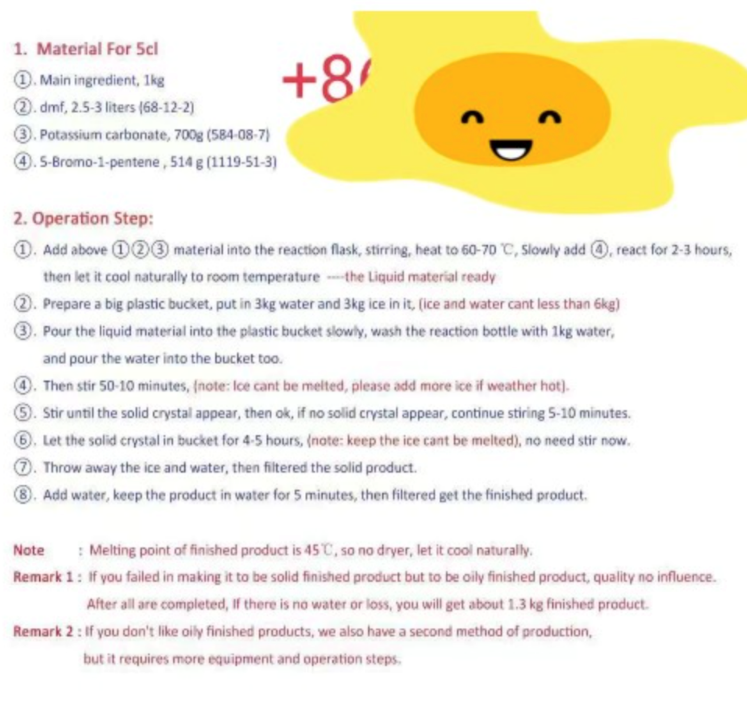
**

**Figure S1.4.** Screenshot of step-by-step instructions for the synthesis of a SCRA from a precursor published on a vendor site.


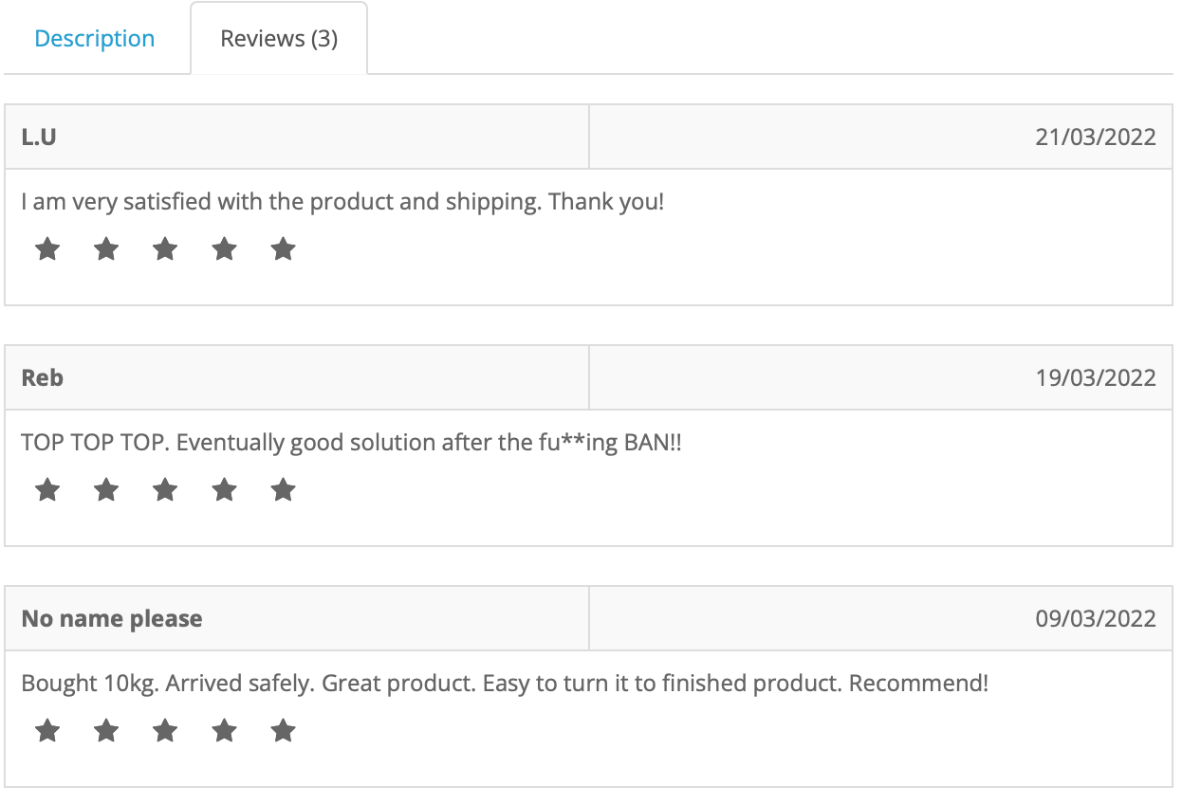

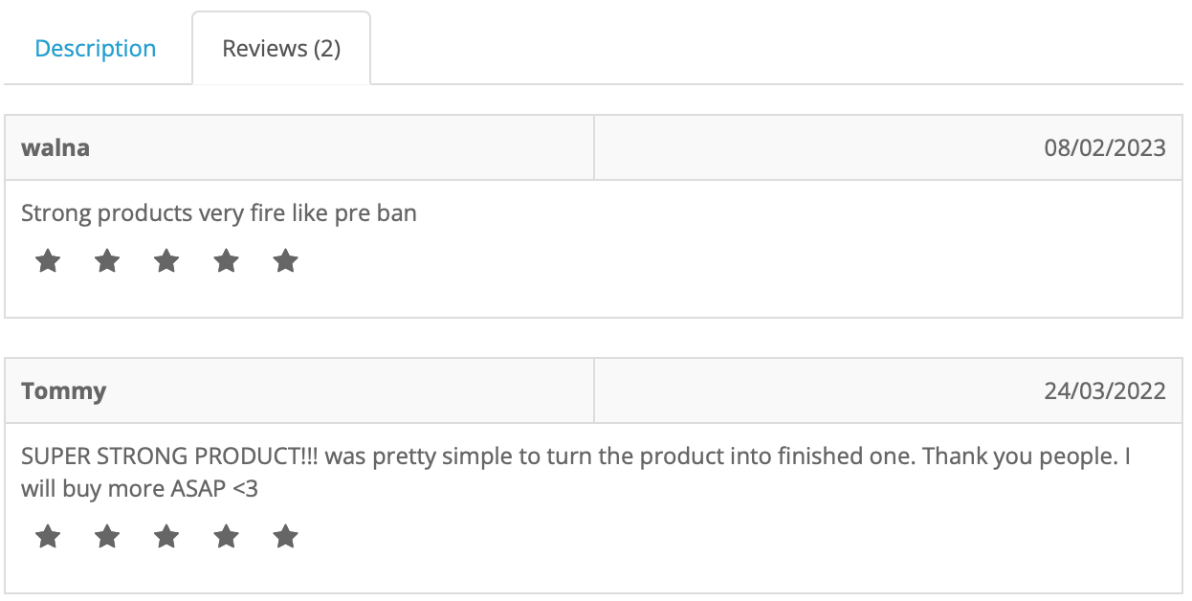


**Figure S1.5.** Reviews on the strength and quality of the synthesized products using precursors as found on vendor websites.

**SECTION 2**

Analytical characterization of synthesized SCRA reference standards

**Table S2.1.** NMR data of the four synthesized SCRAs.

| **SCRA** | **^1^H-NMR chemical shifts** | **^13^C-NMR chemical shifts** |
| --- | --- | --- |
| MDMB-4en-PINACA | ^1^H NMR (500 MHz, CDCl_3_) δ 8.34 (dd, *J* = 8.2, 1.0 Hz, 1H), 7.55 (d, *J* = 9.7 Hz, 1H), 7.42-7.36 (m, 2H), 7.26-7.23 (m, 1H), 5.81 (ddt, *J* = 16.9, 10.3, 6.3 Hz, 1H), 5.09-4.99 (m, 2H), 4.73 (dd, *J* = 9.7, 0.7 Hz, 1H), 4.40 (t, *J* = 6.8 Hz, 2H), 3.75 (s, 3H), 2.12-2.02 (m, 4H), 1.09 (s, 9H). | ^13^C NMR (126 MHz, CDCl_3_) δ 172.25, 162.42, 140.97, 137.13, 136.83, 126.74, 122.99, 122.87, 122.69, 116.00, 109.32, 59.59, 51.91, 51.89, 51.86, 48.69, 35.13, 30.81, 28.81, 26.78. |
| ADB-BUTINACA | ^1^H NMR (500 MHz, CDCl_3_) δ 8.27 (d, *J* = 8.2 Hz, 1H), 7.72 (d, *J* = 9.6 Hz, 1H), 7.45-7.36 (m, 2H), 7.24 (d, *J* = 7.3 Hz, 1H), 6.75 (s, 1H), 5.82 (s, 1H), 4.71 (d, *J* = 9.6 Hz, 1H), 4.39 (t, *J* = 7.2 Hz, 2H), 1.95-1.87 (m, 2H), 1.36 (h, *J* = 7.4 Hz, 2H), 1.16 (s, 9H), 0.95 (t, *J* = 7.4 Hz, 3H). | ^13^C NMR (126 MHz, CDCl_3_) δ 173.14, 162.85, 140.96, 136.59, 126.68, 122.96, 122.69, 122.53, 109.51, 59.69, 49.39, 34.80, 31.90, 26.89, 20.17, 13.75. |
| MDMB-INACA | ^1^H NMR (500 MHz, CDCl_3_) δ 8.33 (dt, *J* = 8.2, 1.0 Hz, 1H), 8.30 (br d, *J* = 9.3 Hz, 1H), 7.50 (dt, J = 8.5, 0.9 Hz, 1H), 7.36 (ddd, *J* = 8.3, 6.8, 1.1 Hz, 1H), 7.23 (ddd, *J* = 8.0, 6.9, 0.9 Hz, 1H), 4.82 (d, *J* = 9.5 Hz, 1H), 3.82 (s, 3H), 1.14 (s, 9H). | ^13^C NMR (126 MHz, CDCl_3_) δ 173.74, 163.31, 141.39, 138.31, 127.16, 122.85, 122.32, 122.19, 110.37, 60.36, 52.30, 34.79, 27.03. |
| ADB-INACA | ^1^H NMR (500 MHz, CD_3_OD) δ 8.20 (dt, *J* = 8.2, 1.1 Hz, 1H), 7.57 (dt, *J* = 8.4, 0.9 Hz, 1H), 7.41 (ddd, *J* = 8.4, 6.9, 1.1 Hz, 1H), 7.25 (ddd, *J* = 8.1, 6.9, 0.9 Hz, 1H), 4.53 (s, 1H), 1.10 (s, 9H). | ^13^C NMR (126 MHz, CD_3_OD) δ 175.13, 164.46, 143.07, 138.99, 127.97, 123.61, 122.93, 122.59, 111.57, 61.06, 35.58, 27.20. |

**Figure S2.1.** Chemical structure, ^1^H-NMR (500 MHz) spectrum, and ^13^C-NMR (126 MHz) spectrum for MDMB-INACA.

**Figure S2.2.** Chemical structure, ^1^H-NMR (500 MHz) spectrum, and ^13^C-NMR (126 MHz) spectrum for MDMB-4en-PINACA.

**Figure S2.3.** Chemical structure, ^1^H-NMR (500 MHz) spectrum, and ^13^C-NMR (126 MHz) spectrum for ADB-INACA.

**Figure S2.4.** Chemical structure, ^1^H-NMR (500 MHz) spectrum, and ^13^C-NMR (126 MHz) spectrum for ADB-BUTINACA.

**SECTION 3**

Corrections for EI-MS detector response for the percentage peak area of SCRAs in seized samples

For samples with a mixture of compounds, the percentage total peak area for each compound was determined by comparing the peak areas of each compound to the total peak area of all active components in the sample. The percentage peak area was then corrected to account for the different EI-MS detector response of each compound by running the samples on the GC-MS alongside a mixture prepared from reference materials at the same concentrations (1 ng/μL for US samples and 100 μg/mL for Scottish samples). Based on comparison of the peak areas of the reference materials, correction factors were calculated and applied to the peak areas of each compound in a sample. The correction factors applied for each compound can be found in Table SX.1 for US samples and Table SX.2 for Scottish samples.

**Table S3.1.** Correction factors applied for the EI-MS detector response to samples from the US for computing the corrected percentage peak area of SCRAs. For all samples containing MDMB-INACA, MDMB-INACA was set to the value of 1 and corrections were applied to the other compounds. For samples not containing MDMB-INACA, but containing MDMB-4en-PINACA, MDMB-4en-PINACA was set to the value of 1 and corrections were applied to the other compounds. Finally, for samples not containing either MDMB-INACA or MDMB-4en-PINACA, ADB-INACA was set to the value of 1 and corrections were applied to the other compounds.

| **Compound** | **Peak Area** | **% Peak Area** | **MDMB-INACA Corrections** | **MDMB-4en-PINACA Corrections** | **ADB-INACA Corrections** |
| --- | --- | --- | --- | --- | --- |
| MDMB-INACA | 272338 | 14.55 | 1.00 | - | 0.21 |
| MDMB-4en-PINACA | 488788 | 26.11 | 0.56 | 1.00 | 0.12 |
| ADB-BUTINACA | 90418 | 4.83 | 3.01 | 5.41 | 0.64 |
| ADB-INACA | 57688 | 3.08 | 4.72 | 8.48 | 1.00 |
| ADB-4en-PINACA | 28655 | 1.53 | 9.51 | 17.07 | 2.01 |
| 4F-MDMB-BUTINACA | 501227 | 26.78 | 0.54 | 0.97 | 0.12 |
| MDMB-BUTINACA | 432594 | 23.11 | 0.63 | 1.13 | 0.13 |

**Table S3.2.** Correction factors applied for the EI-MS detector response to samples from the Scottish prisons for computing the corrected percentage peak area of SCRAs. For all samples containing MDMB-INACA, MDMB-INACA was set to the value of 1 and corrections were applied to the other compounds. For samples not containing MDMB-INACA, but containing MDMB-4en-PINACA, MDMB-4en-PINACA was set to the value of 1 and corrections were applied to the other compounds. For samples containing AB-INACA, AB-INACA was set to the value of 1 and corrections were applied to AB-CHMINACA.

| **Compound** | **MDMB-INACA Corrections** | **MDMB-4en-PINACA Corrections** | **AB-INACA Corrections** |
| --- | --- | --- | --- |
| MDMB-INACA | 1.00 | - | - |
| MDMB-4en-PINACA | 0.47 | 1.00 | - |
| ADB-BUTINACA | 0.68 | 1.46 | - |
| Bromazolam | 1.22 | 2.60 | - |
| MDMB-BUTINACA | 0.61 | - | - |
| MDMB-FUBINACA | 1.11 | - | - |
| AB-INACA | - | - | 1.00 |
| AB-CHMINACA | - | - | 0.07 |

**SECTION 4**

Analysis results of items seized from the clandestine laboratory and related sites.

**Table S4.1.** Analysis results of items seized on 21^st^ December 2022 at the clandestine laboratory site.

| **#** | **Description** | **Weight (g)** | **Analysis results** |
| --- | --- | --- | --- |
| A1 | Yellow powder residue from the industrial cooker | - | ADB-BUTINACA |
| A2 | Yellow powder in two baking molds | 2,367  (net weight) | ADB-BUTINACA |
| A3 | Yellow powder on filter paper | - | ADB-BUTINACA |
| A4 | Sampling of suspension in plastic bucket | ~ 3 L | ADB-BUTINACA |
| A5 | Powder residue from glass suction bottle | - | ADB-BUTINACA |
| A6 | Yellowish powder residues from blue plastic barrels | - | ADB-BUTINACA |
| A7 | Yellowish powder residue from stirrer | - | ADB-BUTINACA |
| A8 | Yellow powder in plastic bag, powder residue in three other bags | 226  (net weight) | ADB-INACA |
| A9 | Powder residues in three aluminum bags | - | No narcotics detected |

**Table S4.2.** Analysis results of items seized on 19^th^ December 2022 from perpetrator’s residence.

| **#** | **Description** | **Weight (g)** | **Analysis results** |
| --- | --- | --- | --- |
| B1 | Inflorescences, dried, in police zip seal bag. Samples from two containers. | 1,590 | MDMB-4en-PINACA on Cannabis (type: industrial hemp) |
| B2 | Inflorescences, dried, in police zip seal bag. Samples from two containers. | 1,180 | ADB-BUTINACA on Cannabis (type: industrial hemp) |
| B3 | Pink powder in vacuum bag  Label: "Shipi, 929" | 927  (net weight) | MDMB-4en-PINACA, 4F-MDMB-BUTICA, 5F-MDMB-PICA and 5F-EMB-PICA in caffeine |
| B4 | Beige powder in zip seal bag | 400  (net weight) | AFUBIATA, MDMB-4en-PINACA, MDMB-5’Br-INACA and ADB-5’Br-INACA |
| B5 | Inflorescences, dried, in police zip seal bag. Samples from two containers. | 1,686 | MDMB-4en-PINACA on Cannabis (type: industrial hemp) |

**Table S4.3.** Analysis results of items seized on 19-20^th^ December 2022 from spraying site.

| **#** | **Description** | **Weight (g)** | **Analysis results** |
| --- | --- | --- | --- |
| C1 | Inflorescences, dried, in police zip seal bag. Sample taken from a bag in a cardboard box. | 9,272 | MDMB-4en-PINACA on Cannabis (type: industrial hemp) |
| C2 |  | 6,446 | MDMB-4en-PINACA on Cannabis (type: industrial hemp) |
| C3 |  | 8,726 | MDMB-4en-PINACA on Cannabis (type: industrial hemp) |
| C4 |  | 7,329 | MDMB-4en-PINACA on Cannabis (type: industrial hemp) |
| C5 |  | 7,127 | MDMB-4en-PINACA on Cannabis (type: industrial hemp) |
| C6 |  | 4,863 | ADB-BUTINACA on Cannabis (type: industrial hemp) |
| C7 |  | 12,388 | MDMB-4en-PINACA on Cannabis (type: industrial hemp) |
| C8 |  | 7,531 | ADB-BUTINACA on Cannabis (type: industrial hemp) |
| C9 |  | 5,073 | MDMB-4en-PINACA on Cannabis (type: industrial hemp) |
| C10 |  | 10,433 | ADB-BUTINACA on Cannabis (type: industrial hemp) |
| C11 |  | 11,111 | JWH-210 on Cannabis (type: industrial hemp) |
| C12 |  | 3,740 | JWH-210 on cannabis resin (hashish, type: drug hemp) |
| C13 |  | 7,448 | ADB-BUTINACA on Cannabis (type: industrial hemp) |
| C14 | Brown resin in three white plastic buckets, ca. 1L, Label: "#16, 402" | 3,055 | ∆^9^-THC |
| C15 | Brown resin in blocks in plastic containers | 3,046 | JWH-210 on cannabis resin (hashish, type: drug hemp) |
| C16 | Brown viscous liquid in jam jar, ca. 1L, Label: «delta9» | 963 | Cannabis extract (type: drug hemp) |
| C17 | Brown viscous liquid in jam jar, ca. 1L, Label: «delta» | 545 | Cannabis extract (type: drug hemp) |
| C18 | Yellow powder in clear plastic bucket | 179 | N-butyl norhexedrone |
| C19 | Yellowish, partly crystalline powder in plastic container,  Label: «2» | 147 | ADB-BUTINACA |
| C20 | Green residues from drying tray | - | ADB-BUTINACA and MDMB-4en-PINACA |
| C21 | Residue in a cup with a blue lid, Label: «3M PPS» | - | ADB-BUTINACA |
| C22 | Yellowish powder in plastic bag | 653 | ADB-BUTINACA |
| C23 | Plant residues in vacuum or zip seal bags | - | ADB-BUTINACA on Cannabis (type: industrial hemp) |
| C24 | Yellowish powder residue | - | ADB-BUTINACA |

**SECTION 5**

Analytical data for the products of the synthesis from the precursors replicated at two independent laboratories (one in Sweden and one in Norway) employing different synthesis durations (5 and 10 h) and temperatures (room temperature (RT) and 70˚C).

**Table S5.1.** Complete analytical data (HPLC and GC-FID) on the purity of the precursors seized from Switzerland and the products of the synthesis from the precursors replicated at two independent laboratories (one in Sweden and one in Norway) employing different reaction conditions (room temperature (RT) 5h, 70˚C 5h, and 70˚C 10 h). There is no GC-FID purity data for the ADB-BUTINACA products as ADB-INACA and ADB-BUTINACA co-eluted.

| **Country** | **Compound** | **Conditions** | **HPLC purity** | | | | **GC-FID purity** | | | | **Average** |
| --- | --- | --- | --- | --- | --- | --- | --- | --- | --- | --- | --- |
|  |  |  | **1** | **2** | **3** | **Average** | **1** | **2** | **3** | **Average** |  |
| Switzerland | ADB-INACA | Seizure | 95.05% | 95.20% | 95.19% | 95.15% | 98.24% | 98.14% | 98.10% | 98.16% | 96.65% |
|  | MDMB-INACA | Seizure | 97.53% | 97.51% | 97.59% | 97.54% | 98.93% | 98.85% | 98.65% | 98.81% | 98.18% |
| Norway | ADB-BUTINACA | RT 5 h | 56.91% | 56.77% | 56.86% | 56.85% | N/A | | | | 56.85% |
|  |  | 70˚C 5 h | 39.19% | 39.20% | 39.51% | 39.30% |  |  |  |  | 39.30% |
|  |  | 70˚C 10 h | 64.96% | 65.22% | 65.35% | 65.18% |  |  |  |  | 65.18% |
|  | MDMB-4en-PINACA | RT 5 h | 45.63% | 45.92% | 45.87% | 45.81% | 57.41% | 57.35% | 57.25% | 57.34% | 51.57% |
|  |  | 70˚C 5 h | 53.01% | 52.89% | 52.91% | 52.94% | 63.24% | 62.94% | 62.72% | 62.97% | 57.95% |
|  |  | 70˚C 10 h | 55.62% | 55.18% | 55.33% | 55.38% | 65.41% | 65.34% | 65.34% | 65.36% | 60.37% |
| Sweden | ADB-BUTINACA | RT 5 h | 86.11% | 86.45% | 86.19% | 86.25% | N/A | | | | 86.25% |
|  |  | 70˚C 5 h | 75.72% | 75.43% | 75.24% | 75.46% |  |  |  |  | 75.46% |
|  |  | 70˚C 10 h | 92.87% | 92.01% | 92.93% | 92.60% |  |  |  |  | 92.60% |
|  | MDMB-4en-PINACA | RT 5 h | 87.47% | 87.49% | 87.45% | 87.47% | 92.08% | 92.07% | 91.98% | 92.04% | 89.76% |
|  |  | 70˚C 5 h | 92.48% | 92.50% | 92.33% | 92.44% | 95.40% | 95.26% | 95.29% | 95.32% | 93.88% |
|  |  | 70˚C 10 h | 87.94% | 87.69% | 88.32% | 87.98% | 95.57% | 95.59% | 95.49% | 95.55% | 91.77% |


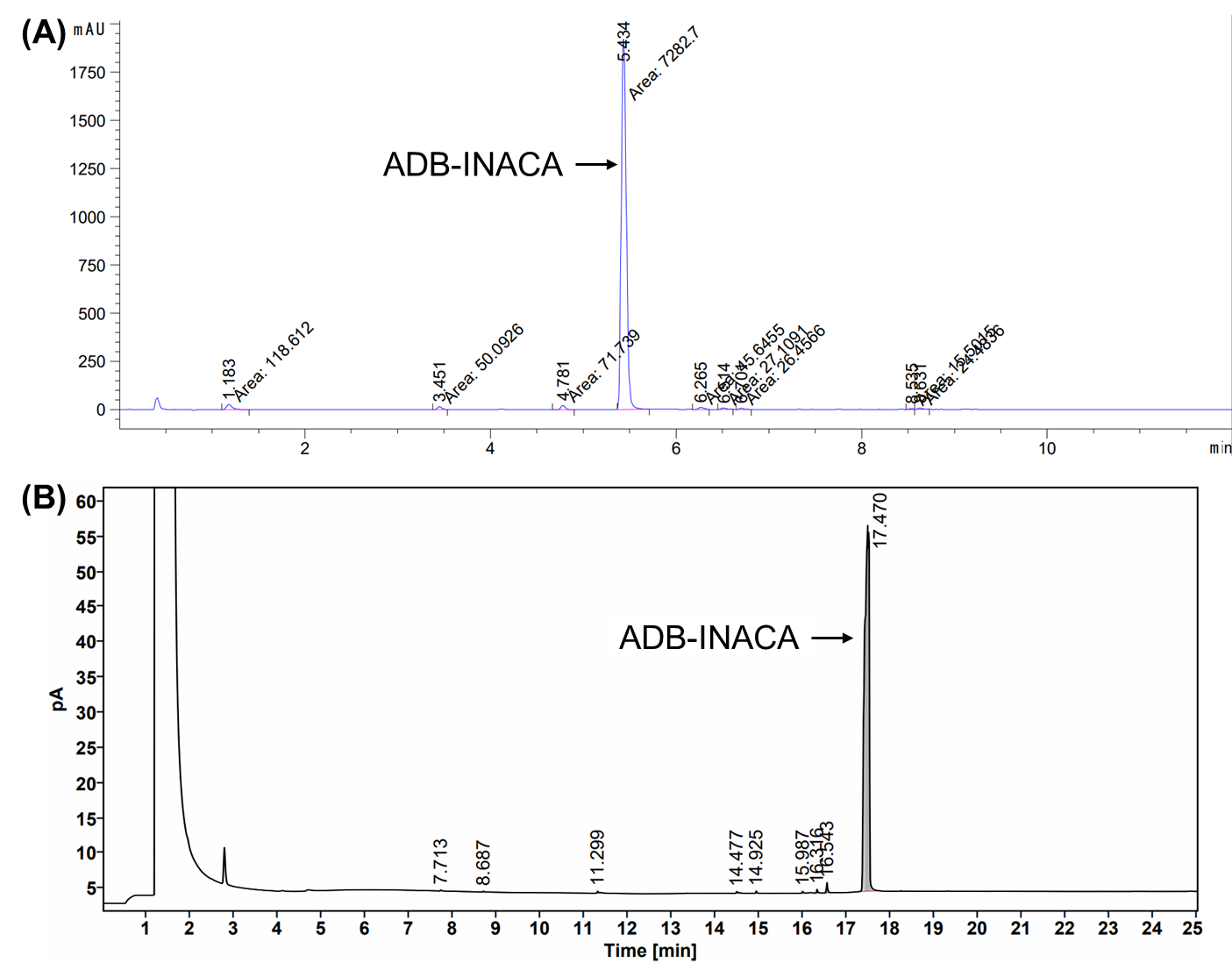


**Figure S5.1.** (A) HPLC chromatogram and (B) GC-FID chromatogram of the ADB-INACA seized from the clandestine laboratory in Switzerland and used for the replicated synthesis.


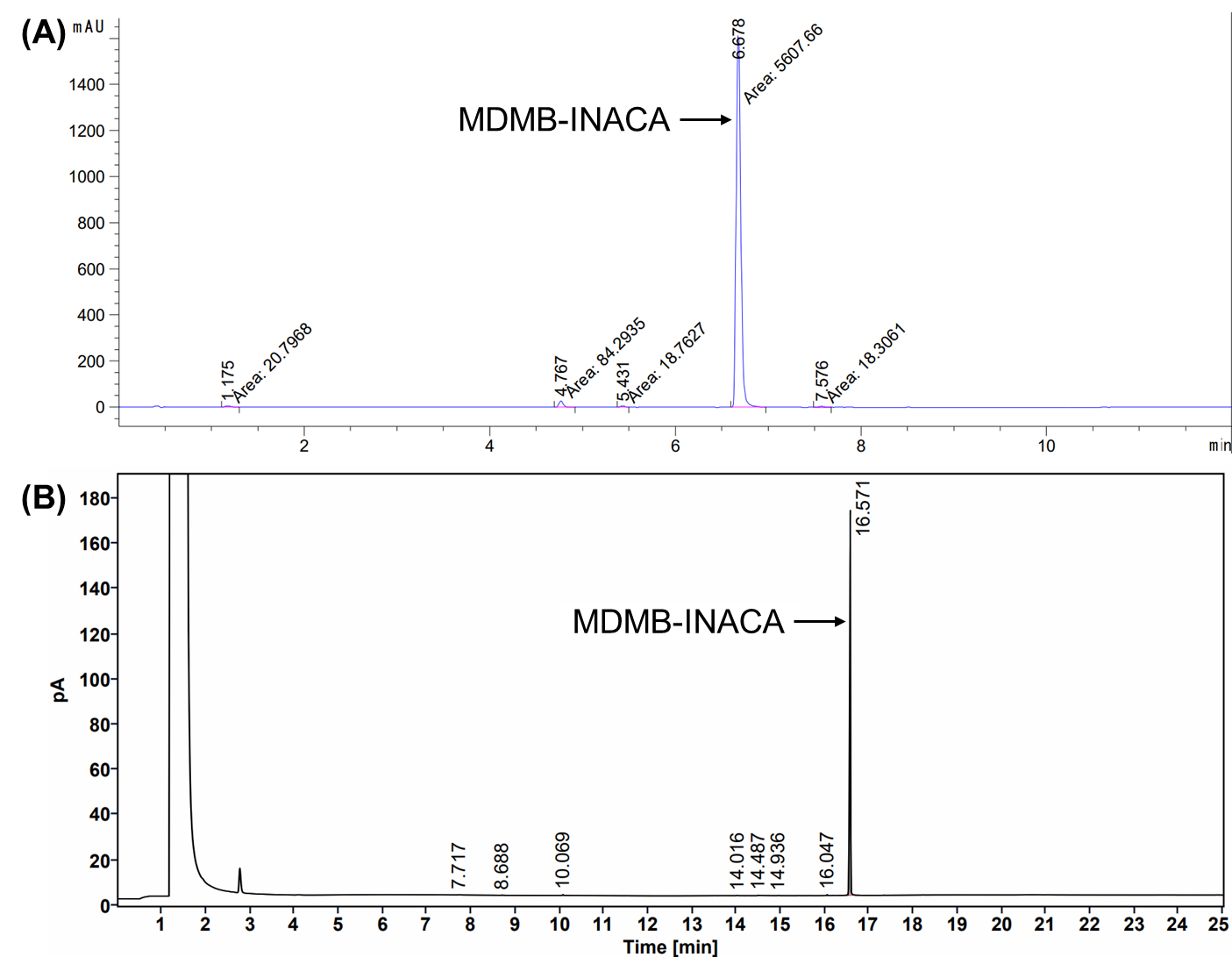


**Figure S5.2.** (A) HPLC chromatogram and (B) GC-FID chromatogram of the MDMB-INACA seized from the clandestine laboratory in Switzerland and used for the replicated synthesis.


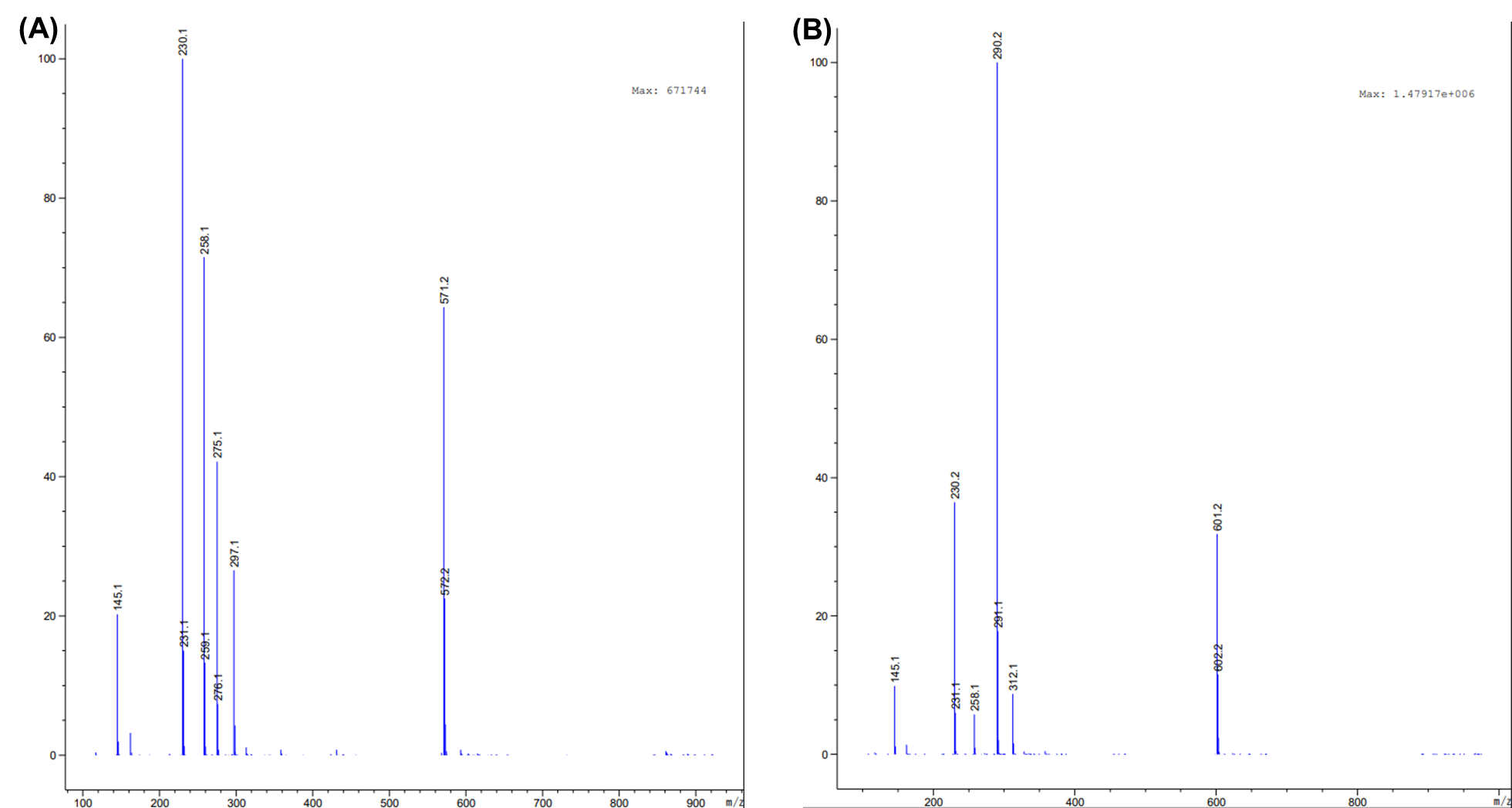


**Figure S5.3.** MS spectrum from HPLC-MS analysis for (A) ADB-INACA) and (B) MDMB-INACA seized from the clandestine laboratory in Switzerland and used for the replicated synthesis.


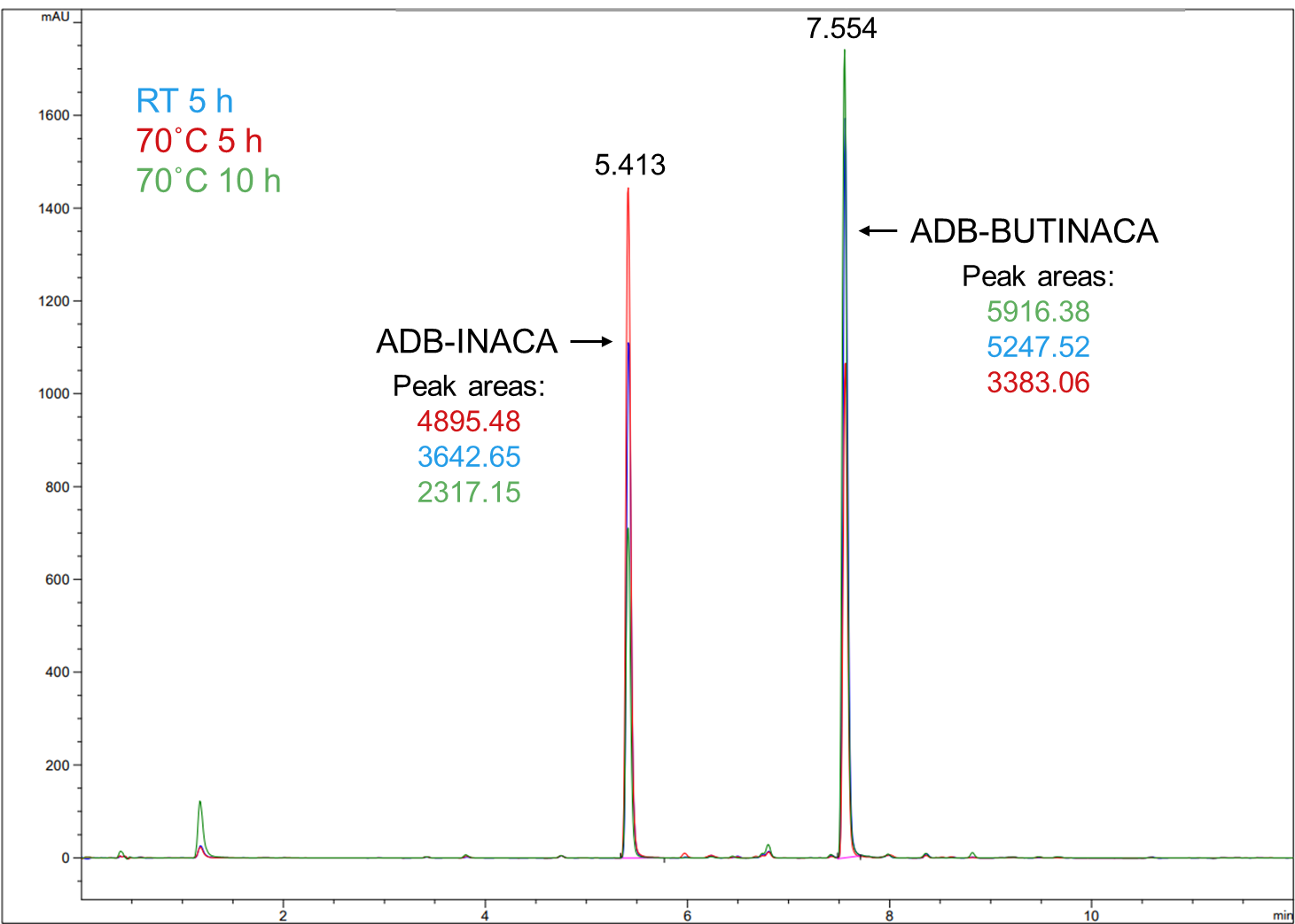


**Figure S5.4.** Overlaid HPLC chromatogram of the ADB-BUTINACA products from the replicated synthesis in Norway using different reaction conditions (RT 5 h, 70˚C 5 h, and 70˚C 10 h).


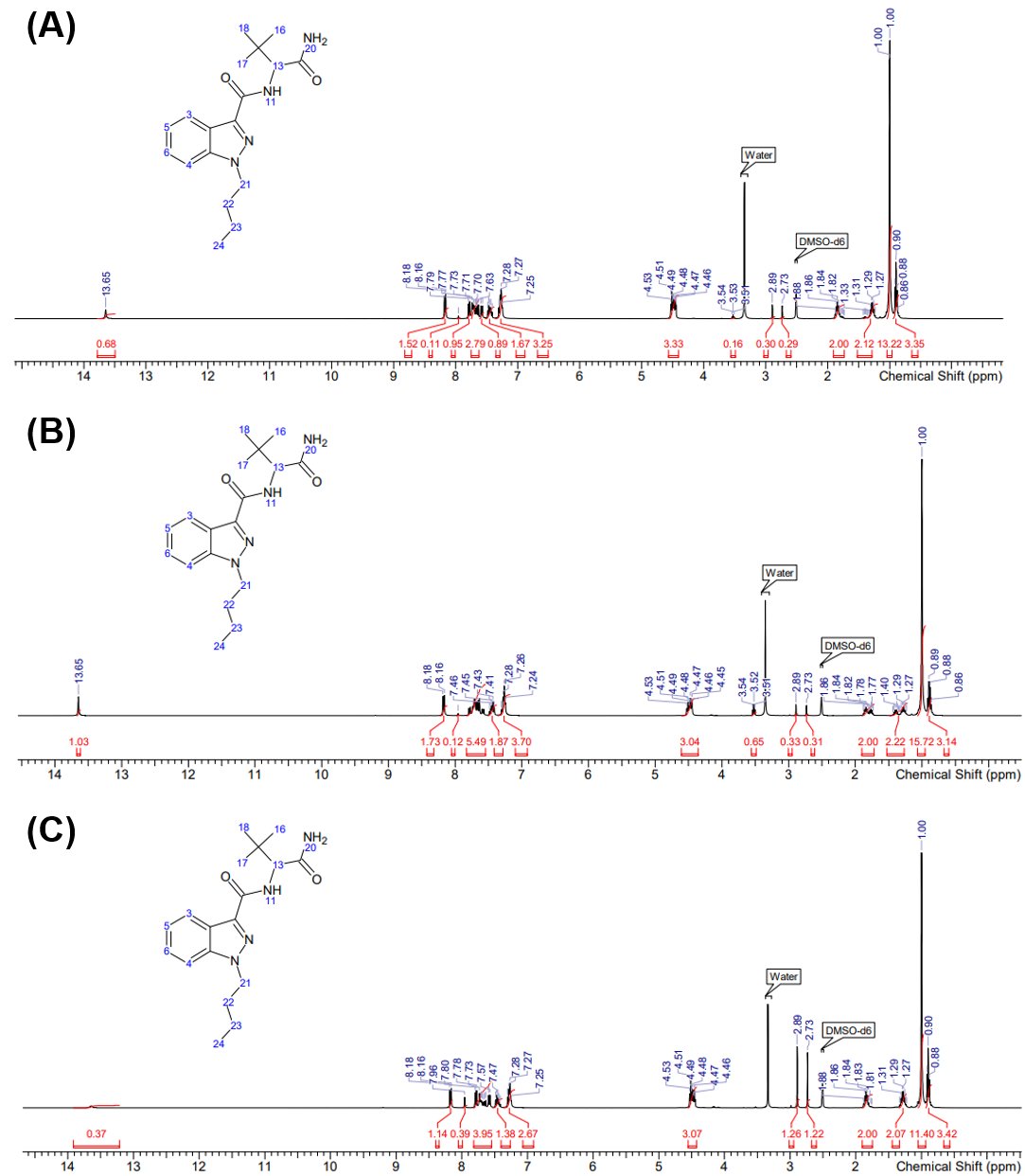


**Figure S5.5.** NMR spectra of the ADB-BUTINACA products from the replicated synthesis in Norway using different reaction conditions (A) RT 5 h, (B) 70˚C 5 h, and (C) 70˚C 10 h. Samples were prepared in DMSO-d6.


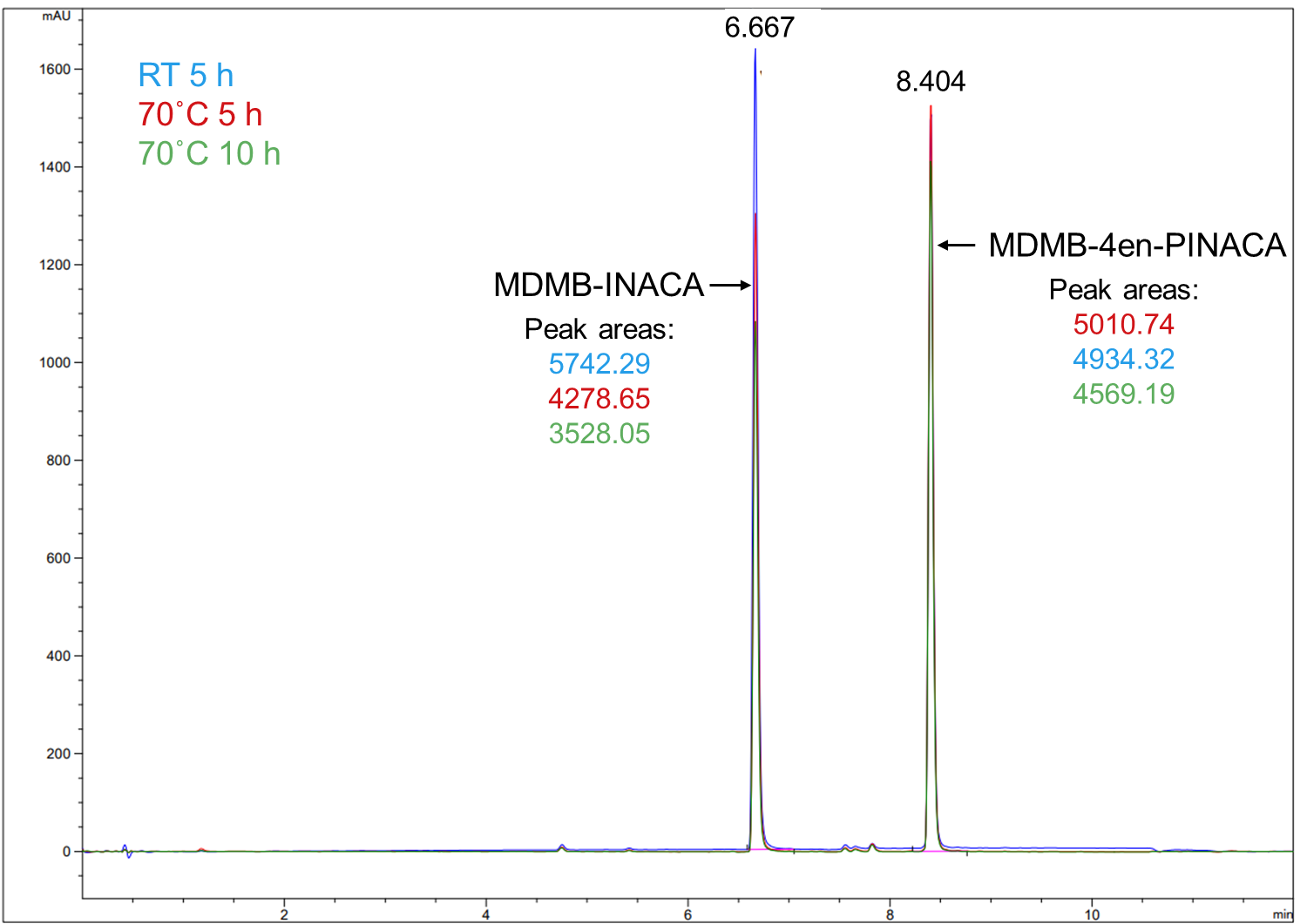


**Figure S5.6.** Overlaid HPLC chromatogram of the MDMB-4en-PINACA products from the replicated synthesis in Norway using different reaction conditions (RT 5 h, 70˚C 5 h, and 70˚C 10 h).


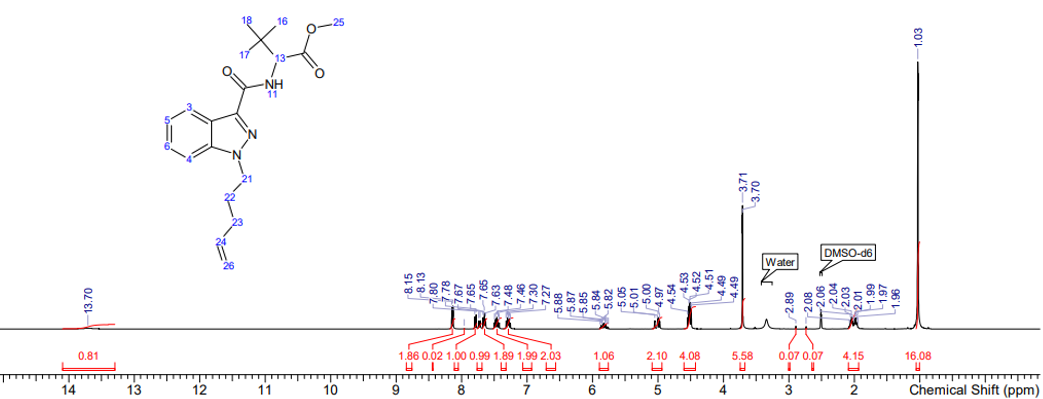
**Figure S5.7.** Example NMR spectrum of the MDMB-4en-PINACA products from the replicated synthesis in Norway (70˚C 5 h). Samples were prepared in DMSO-d6.


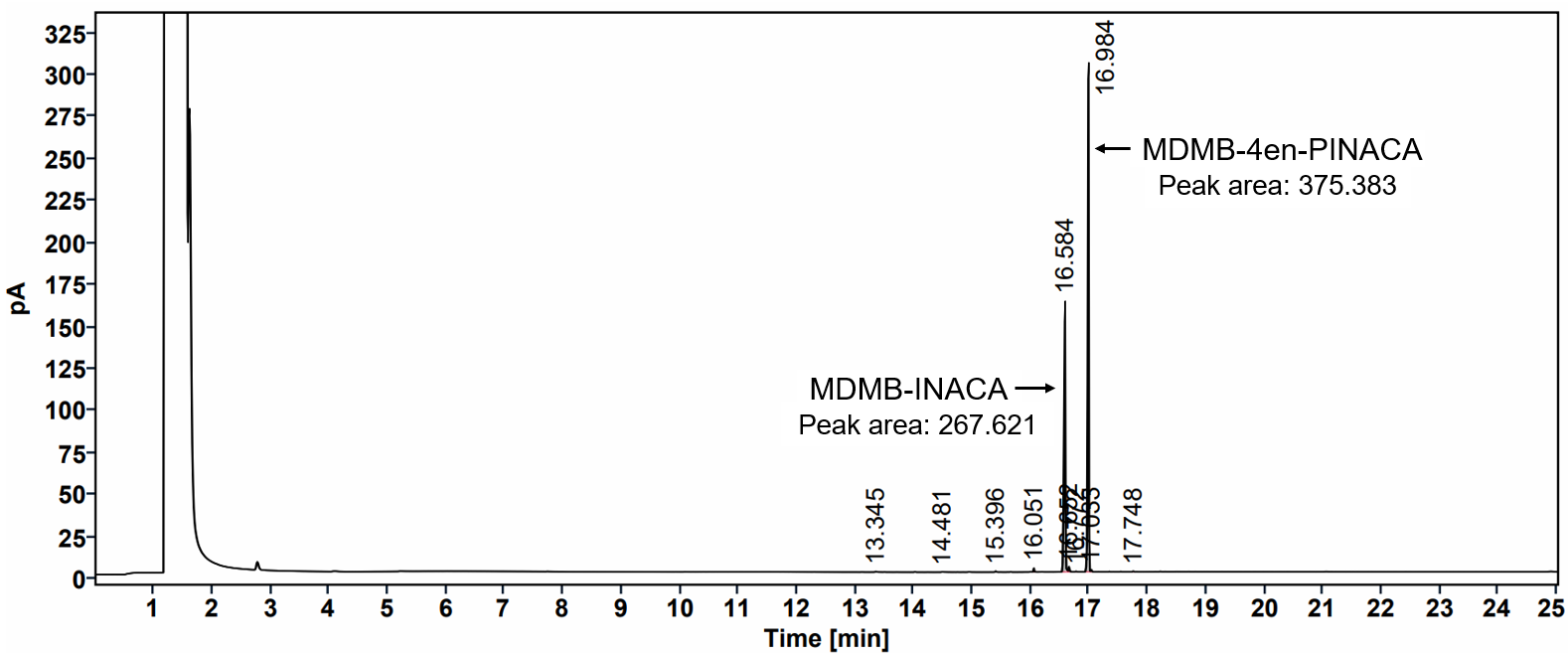


**Figure S5.8.** Example GC-FID chromatogram of a MDMB-4en-PINACA product from the replicated synthesis (RT 5 h from Norway).


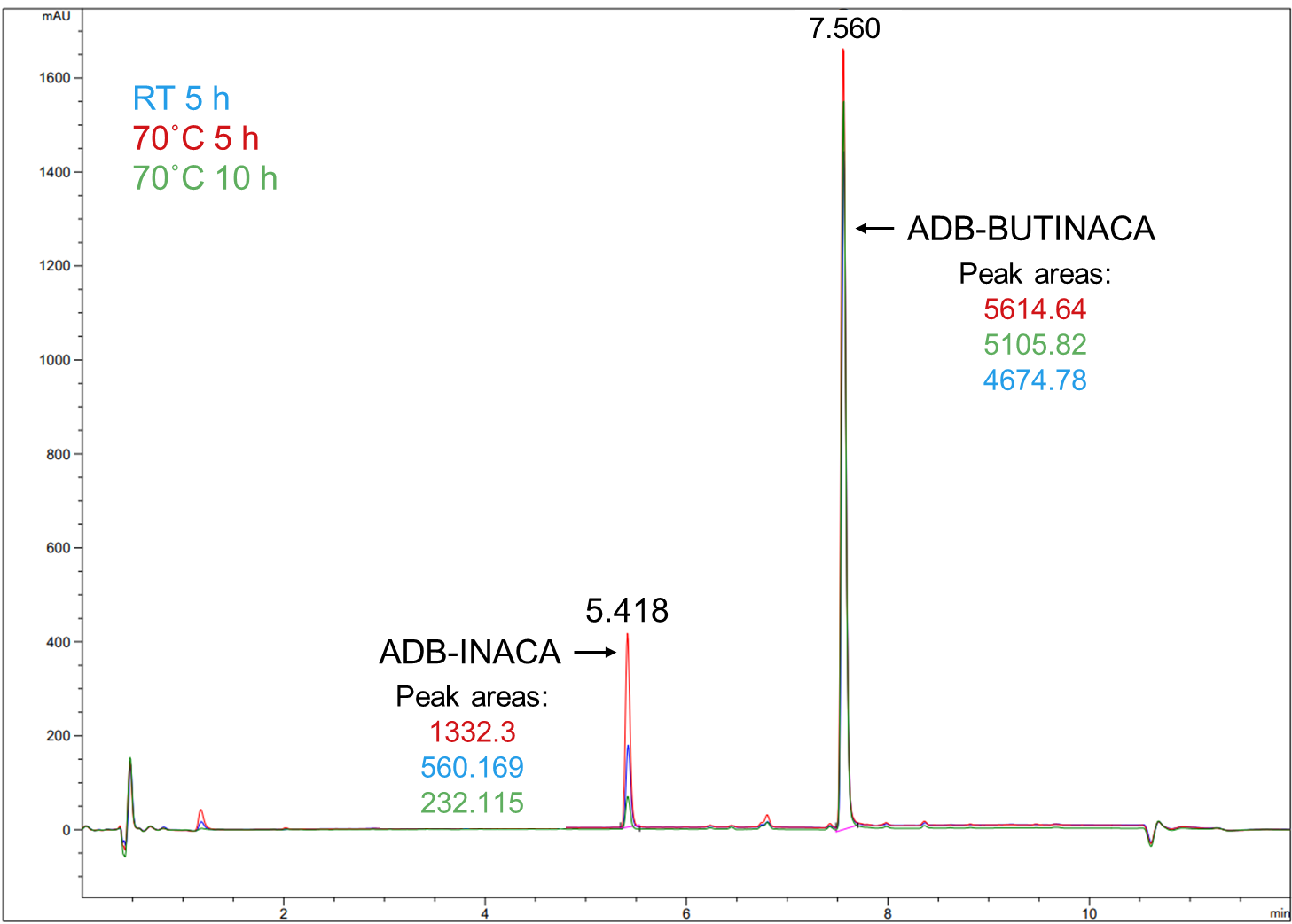


**Figure S5.9.** Overlaid HPLC chromatogram of the ADB-BUTINACA products from the replicated synthesis in Sweden using different reaction conditions (RT 5 h, 70˚C 5 h, and 70˚C 10 h).


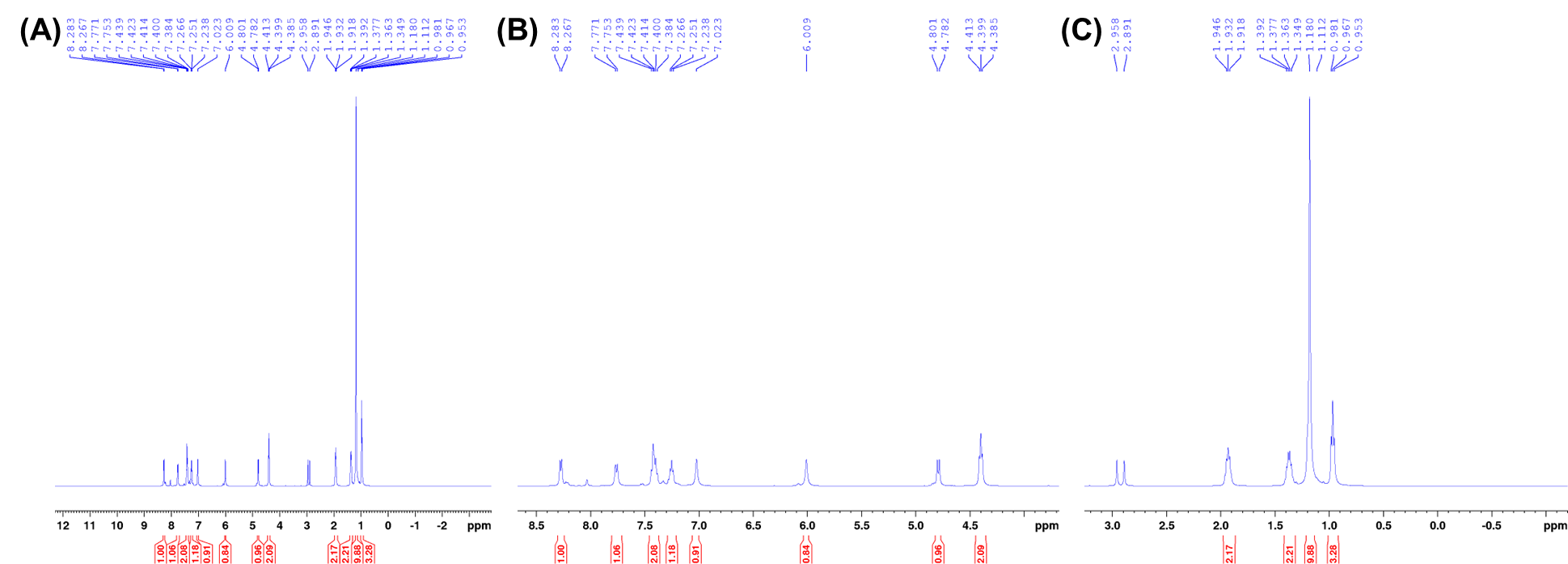


**Figure S5.10.** NMR spectra of the RT 5 h ADB-BUTINACA product from the replicated synthesis in Sweden: (A) full spectrum, (B) zoom-in from 4.5-8.5 ppm, and (C) zoom-in from 0.0-3.0 ppm.


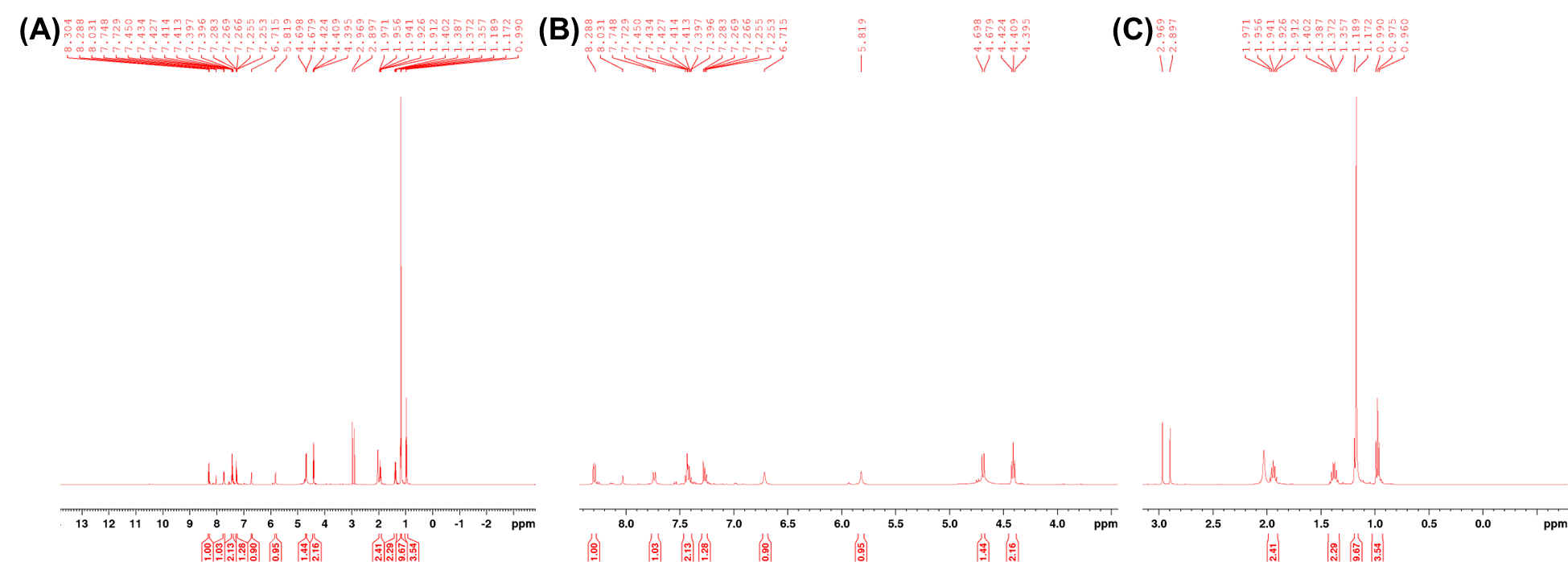


**Figure S5.11.** NMR spectra of the 70˚C 5 h ADB-BUTINACA product from the replicated synthesis in Sweden: (A) full spectrum, (B) zoom-in from 4.5-8.5 ppm, and (C) zoom-in from 0.0-3.0 ppm.


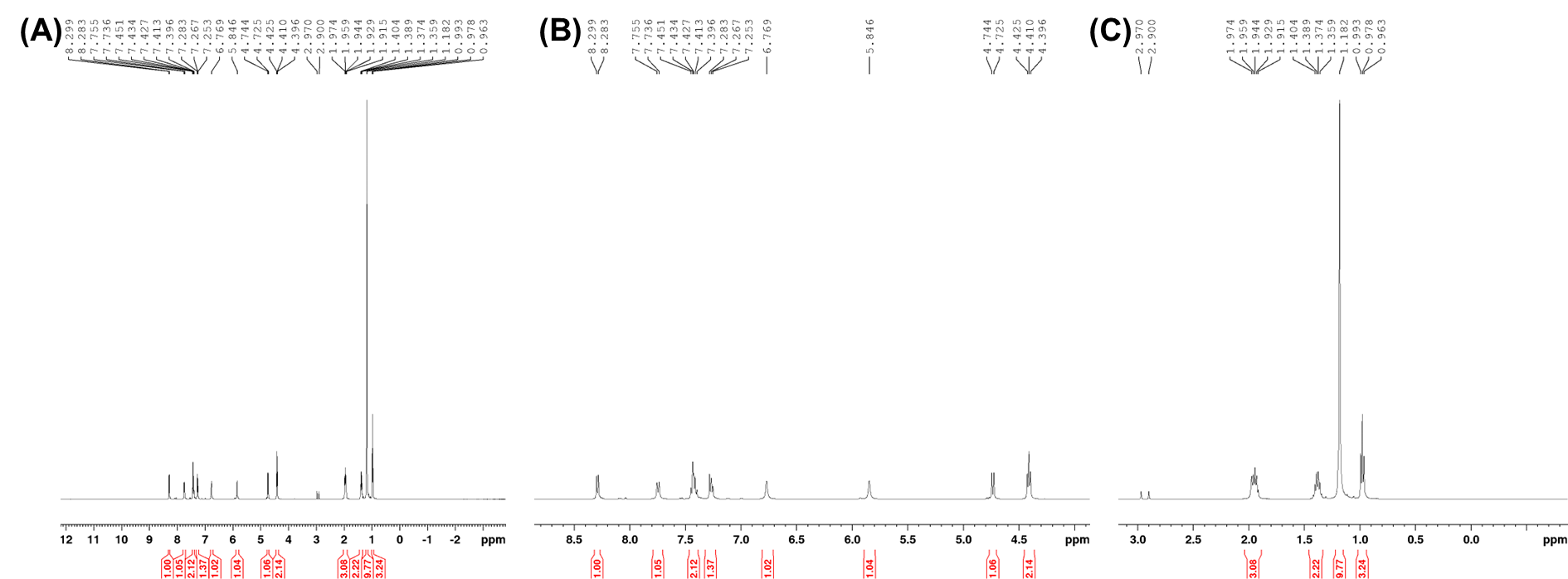


**Figure S5.12.** NMR spectra of the 70˚C 10 h ADB-BUTINACA product from the replicated synthesis in Sweden: (A) full spectrum, (B) zoom-in from 4.5-8.5 ppm, and (C) zoom-in from 0.0-3.0 ppm.


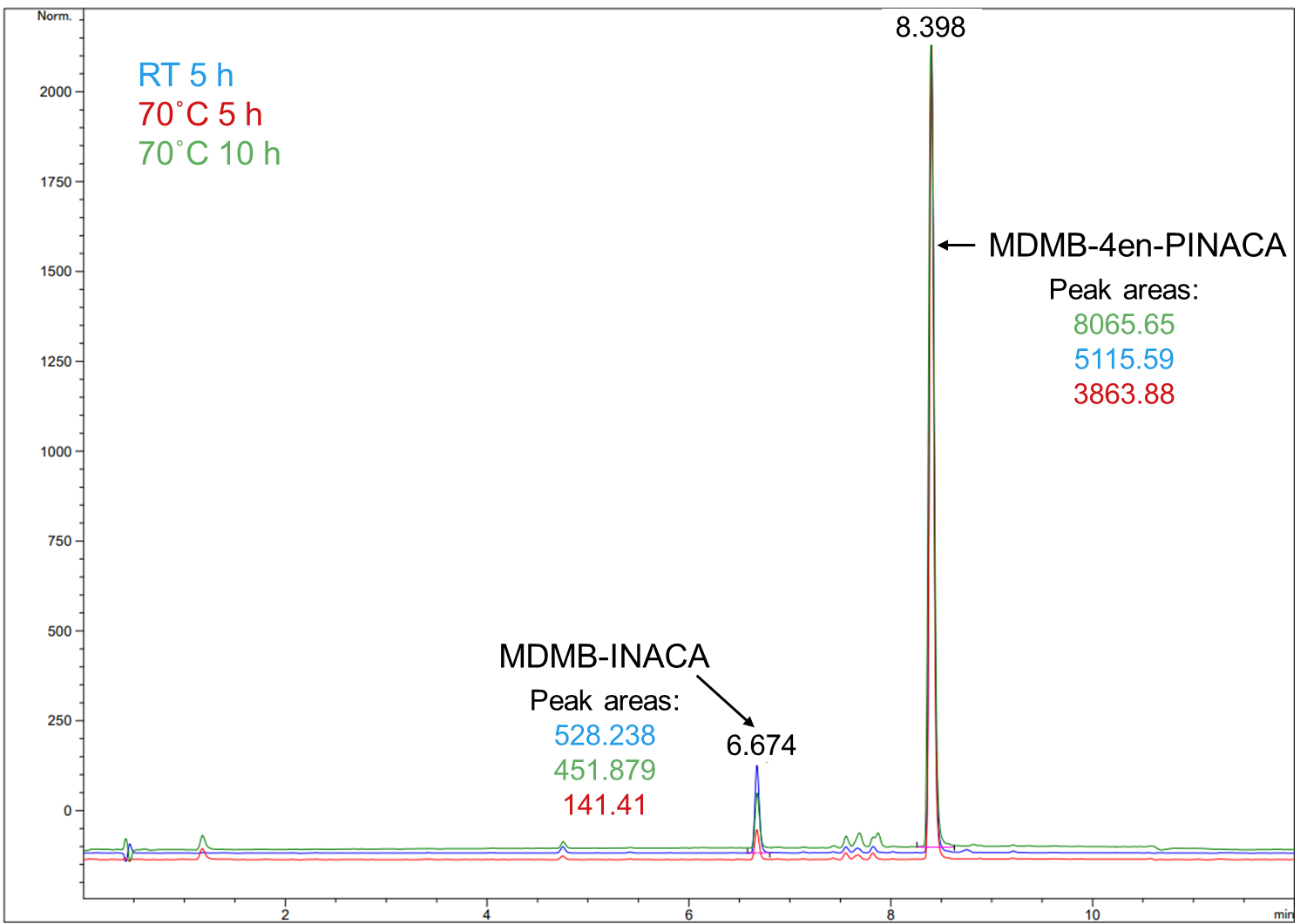


**Figure S5.13.** Overlaid HPLC chromatogram of the MDMB-4en-PINACA products from the replicated synthesis in Sweden using different reaction conditions (RT 5 h, 70˚C 5 h, and 70˚C 10 h).


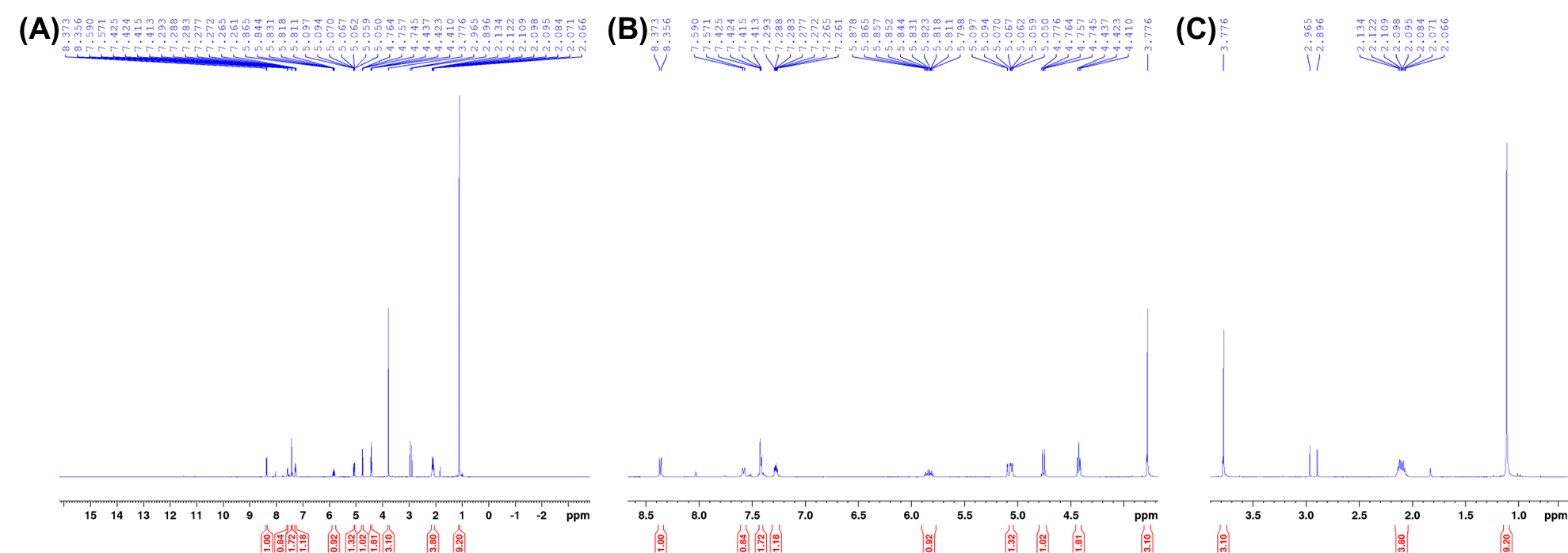


**Figure S5.14.** NMR spectra of the RT 5 h MDMB-4en-PINACA product from the replicated synthesis in Sweden: (A) full spectrum, (B) zoom-in from 4.5-8.5 ppm, and (C) zoom-in from 0.0-4.0 ppm.


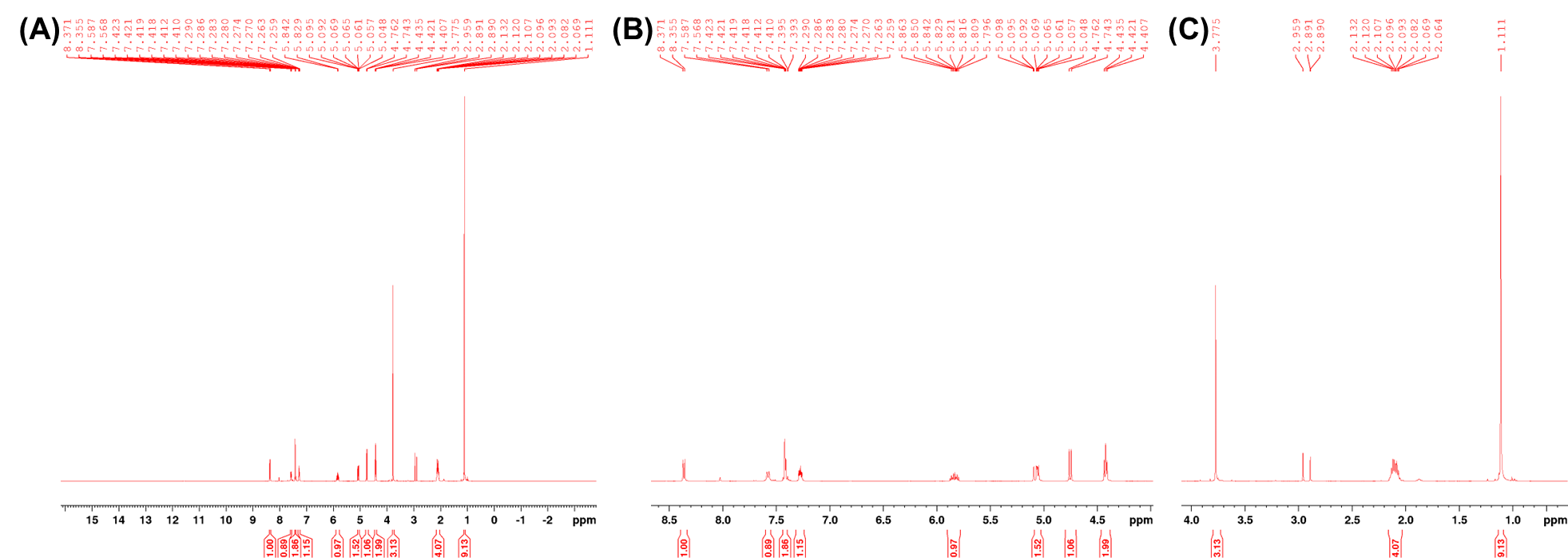


**Figure S5.15.** NMR spectra of the 70˚C 5 h MDMB-4en-PINACA product from the replicated synthesis in Sweden: (A) full spectrum, (B) zoom-in from 4.5-8.5 ppm, and (C) zoom-in from 0.0-4.0 ppm.


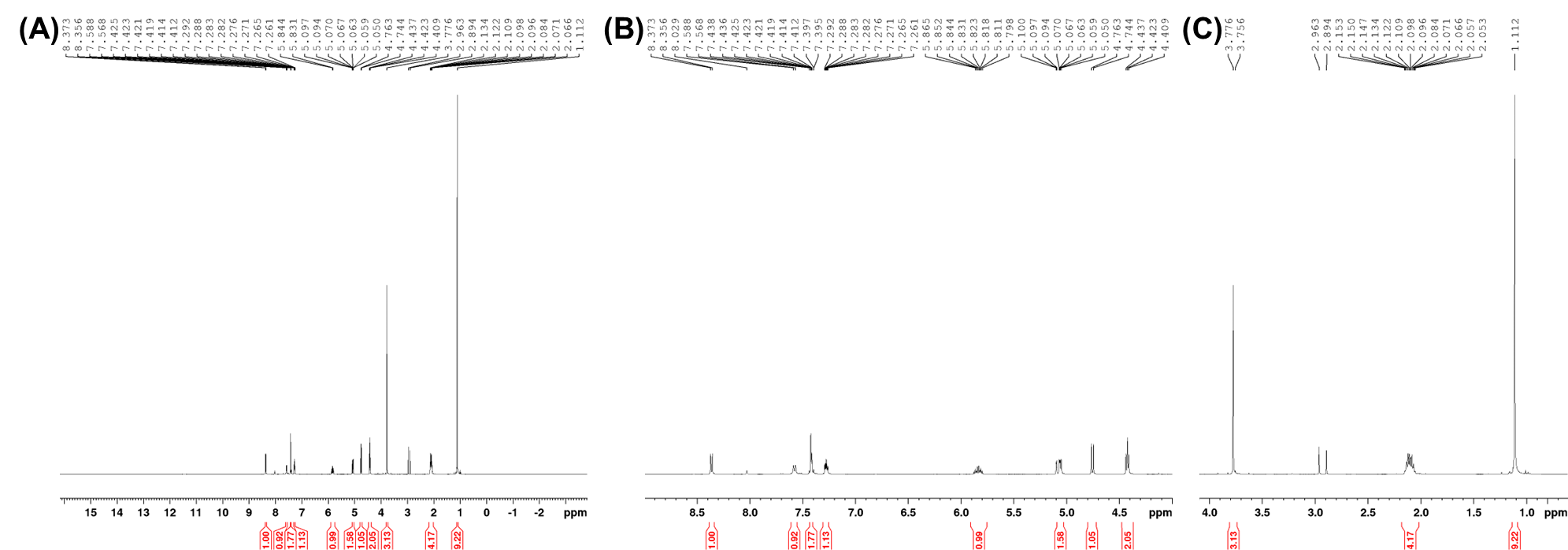


**Figure S5.16.** NMR spectra of the 70˚C 10 h MDMB-4en-PINACA product from the replicated synthesis in Sweden: (A) full spectrum, (B) zoom-in from 4.5-8.5 ppm, and (C) zoom-in from 0.0-4.0 ppm.

**SECTION 6**

Complete data for the US seized samples found positive for a tail-less SCRA/precursor

**Table S6.1.** Complete data for the US seized samples found positive for a tail-less SCRA/precursor organized by seizure date then processed date. The percentage of the peak area of the SCRAs are provided before and after correcting for EI-MS detector response. The corrections can be found in Table S2.1. The percentage of the final SCRA versus the corresponding precursor is provided based on the corrected percentage of peak area except where the reference standard for the precursor was unavailable at the time of testing (indicated with *).

| **No** | **Place** | **Sample Type** | **Seizure Date** | **Processed Date** | **Results** | **Peak Area** | **% Peak Area** | **Corrected % Peak Area** | **% Final of Precursor** |
| --- | --- | --- | --- | --- | --- | --- | --- | --- | --- |
| 1 | IL | Paper | 2022-02-15 | N/A | 4F-MDMB-BUTICA | 80193 | 99.89 | - | 99.89* |
|  |  |  |  |  | MDMB-ICA | 92 | 0.11 | - |  |
| 2 | IL | Paper | 2022-02-15 | N/A | 4F-MDMB-BUTICA | 82476 | 99.85 | - | 99.85* |
|  |  |  |  |  | MDMB-ICA | 120 | 0.15 | - |  |
| 3 | IL | Paper | 2022-02-15 | N/A | 4F-MDMB-BUTICA | 68531 | 99.90 | - | 99.90* |
|  |  |  |  |  | MDMB-ICA | 70 | 0.10 | - |  |
| 4 | IL | Paper | 2023-01-07 | N/A | MDMB-4en-PINACA | 76903 | 92.68 | 71.00 | 99.09 |
|  |  |  |  |  | MDMB-INACA | 394 | 0.47 | 0.65 |  |
|  |  |  |  |  | ADB-BUTINACA | 5679 | 6.84 | 28.34 | - |
|  |  |  |  |  | NMDMSB | - | - | - | - |
|  |  |  |  |  | Ibuprofen | - | - | - | - |
|  |  |  |  |  | Caffeine | - | - | - | - |
|  |  |  |  |  | Lidocaine | - | - | - | - |
| 5 | IL | Paper | 2023-01-09 | N/A | MDMB-4en-PINACA | 1042537 | 99.94 | 99.90 | 99.90 |
|  |  |  |  |  | MDMB-INACA | 576 | 0.06 | 0.10 |  |
|  |  |  |  |  | CH-PIATA | - | - | - | - |
|  |  |  |  |  | Bromazolam | - | - | - | - |
| 6 | PA | Herbal material | 2023-02-01 | 2023-02-03 | ADB-BUTINACA | 26202 | 86.07 | 79.76 | 79.76 |
|  |  |  |  |  | ADB-INACA | 4241 | 13.93 | 20.24 |  |
|  |  |  |  |  | N,N-Dimethylpentylone | - | - | - | - |
| 7 | PA | Herbal material | 2023-02-01 | 2023-02-03 | ADB-BUTINACA | 13383 | 90.29 | 91.06 | 92.02 |
|  |  |  |  |  | ADB-INACA | 740 | 4.99 | 7.90 |  |
|  |  |  |  |  | MDMB-BUTINACA | 644 | 4.34 | 0.92 | 88.06 |
|  |  |  |  |  | MDMB-INACA | 55 | 0.37 | 0.12 |  |
| 8 | PA | Herbal material | 2023-02-08 | 2023-04-25 | ADB-BUTINACA | 12474 | 70.21 | 60.05 | 60.05 |
|  |  |  |  |  | ADB-INACA | 5293 | 29.79 | 39.95 |  |
| 9 | PA | Herbal material | 2023-02-08 | 2023-04-25 | MDMB-4en-PINACA | 15199 | 94.23 | 70.81 | 93.09 |
|  |  |  |  |  | MDMB-INACA | 629 | 3.90 | 5.26 |  |
|  |  |  |  |  | ADB-4en-PINACA | 301 | 1.87 | 23.93 | - |
| 10 | PA | Herbal material | 2023-02-22 | 2023-03-16 | MDMB-4en-PINACA | 13862 | 98.70 | 97.69 | 97.69 |
|  |  |  |  |  | MDMB-INACA | 183 | 1.30 | 2.31 |  |
| 11 | PA | Herbal material | 2023-03-22 | 2023-04-19 | ADB-BUTINACA | 3135 | 52.19 | 41.30 | 41.34 |
|  |  |  |  |  | ADB-INACA | 2837 | 47.23 | 58.61 |  |
|  |  |  |  |  | MDMB-BUTINACA | 35 | 0.58 | 0.10 | - |
| 12 | PA | Herbal material | 2023-03-22 | 2023-04-19 | MDMB-4en-PINACA | 700 | 80.00 | 69.03 | 69.03 |
|  |  |  |  |  | MDMB-INACA | 175 | 20.00 | 30.97 |  |
| 13 | PA | Herbal material | 2023-07-08 | 2023-08-15 | ADB-4en-PINACA | 6981 | 79.37 | 97.73 | 99.10 |
|  |  |  |  |  | ADB-INACA | 128 | 1.46 | 0.89 |  |
|  |  |  |  |  | MDMB-4en-PINACA | 1686 | 19.17 | 1.38 | - |
| 14 | IL | Paper | 2023-10-05 | N/A | MDMB-4en-PINACA | 76898 | 50.71 | 36.44 | 36.44 |
|  |  |  |  |  | MDMB-INACA | 74735 | 49.29 | 63.56 |  |
| 15 | IL | Paper | 2023-10-19 | N/A | MDMB-4en-PINACA | 45957 | 98.86 | 97.97 | 97.97 |
|  |  |  |  |  | MDMB-INACA | 530 | 1.14 | 2.03 |  |
| 16 | IL | Paper | 2023-11-10 | N/A | ADB-FUBIATA | 100494 | 99.96 | - | 99.96* |
|  |  |  |  |  | ADB-IATA | 43 | 0.04 | - |  |
|  |  |  |  |  | Fentanyl | - | - | - |  |
| 17 | IL | Paper | 2023-11-11 | N/A | MDMB-4en-PINACA | 188812 | 99.09 | 98.37 | 98.37 |
|  |  |  |  |  | MDMB-INACA | 1743 | 0.91 | 1.63 |  |
| 18 | IN | Herbal material | N/A | 2021-11-12 | ADB-5'Br-INACA | - | - | - | - |
| 19 | N/A | Herbal material | N/A | N/A | CH-PIATA | - | - | - | - |
|  |  |  |  |  | ADB-BUTINACA |  |  |  |  |
|  |  |  |  |  | ADB-5'Br-INACA |  |  |  |  |
|  |  |  |  |  | ADB-FUBIATA |  |  |  |  |
|  |  |  |  |  | BZO-POXIZID |  |  |  |  |
|  |  |  |  |  | BZO-CHMOXIZID |  |  |  |  |
|  |  |  |  |  | EG-018 |  |  |  |  |
|  |  |  |  |  | Xylazine |  |  |  |  |
|  |  |  |  |  | Fentanyl |  |  |  |  |
|  |  |  |  |  | 4-ANPP |  |  |  |  |
| 20 | IN | Herbal material | N/A | 2022-03-23 | MDMB-5'Br-INACA | - | - | - | - |
| 21 | N/A | N/A | N/A | 2022-12-05 | ADB-INACA | - | - | - | - |
| 22 | Abu Dhabi | Herbal material | N/A | 2023-02-01 | ADB-5'Br-PINACA | 25887 | 81.26 | - | - |
|  |  |  |  |  | ADB-INACA | 5971 | 18.74 | - | - |
| 23 | N/A | N/A | N/A | 2023-04-06 | MDMB-4en-PINACA | 955821 | 44.67 | 31.03 | 31.03 |
|  |  |  |  |  | MDMB-INACA | 1184088 | 55.33 | 68.97 |  |
| 24 | N/A | N/A | N/A | 2023-08-28 | MDMB-5Me-INACA | - | - | - | - |
| 25 | NJ | Liquid | N/A | 2023-09-21 | ADB-4en-PINACA | 309308 | 53.98 | 86.92 | 89.82 |
|  |  |  |  |  | ADB-INACA | 70600 | 12.32 | 9.85 |  |
|  |  |  |  |  | MDMB-4en-PINACA | 189344 | 33.04 | 3.12 | 96.57 |
|  |  |  |  |  | MDMB-INACA | 3749 | 0.65 | 0.11 |  |
|  |  |  |  |  | CBD | - | - | - | - |
|  |  |  |  |  | THC | - | - | - | - |
|  |  |  |  |  | Tianeptine | - | - | - | - |
| 26 | NJ | Liquid | N/A | 2023-09-21 | ADB-4en-PINACA | 219813 | 56.49 | 86.08 | 88.38 |
|  |  |  |  |  | ADB-INACA | 58177 | 14.95 | 11.32 |  |
|  |  |  |  |  | MDMB-4en-PINACA | 108210 | 27.81 | 2.48 | 95.42 |
|  |  |  |  |  | MDMB-INACA | 2892 | 0.74 | 0.12 |  |
|  |  |  |  |  | CBD | - | - | - | - |
|  |  |  |  |  | THC | - | - | - | - |
|  |  |  |  |  | Tianeptine | - | - | - | - |
| 27 | GA | Paper | N/A | 2023-11-10 | MDMB-4en-PINACA | 6488186 | 93.94 | 68.95 | 98.86 |
|  |  |  |  |  | MDMB-INACA | 41518 | 0.60 | 0.79 |  |
|  |  |  |  |  | ADB-BUTINACA | 246795 | 3.57 | 14.18 | 68.03 |
|  |  |  |  |  | ADB-4en-PINACA | 23147 | 0.51 | 6.41 |  |
|  |  |  |  |  | ADB-INACA | 107359 | 1.55 | 9.67 |  |
| 28 | GA | Paper | N/A | 2023-11-10 | MDMB-4en-PINACA | 4106205 | 92.29 | 41.44 | 98.82 |
|  |  |  |  |  | MDMB-INACA | 27343 | 0.61 | 0.50 |  |
|  |  |  |  |  | ADB-BUTINACA | 224308 | 5.04 | 12.24 | 90.07 |
|  |  |  |  |  | ADB-4en-PINACA | 23940 | 5.23 | 40.06 |  |
|  |  |  |  |  | ADB-INACA | 67364 | 1.51 | 5.76 |  |
| 29 | GA | Paper | N/A | 2023-11-10 | MDMB-4en-PINACA | 6030064 | 87.57 | 32.75 | 99.82 |
|  |  |  |  |  | 4F-MDMB-BUTINACA | 56609 | 1.03 | 0.38 |  |
|  |  |  |  |  | MDMB-INACA | 6142 | 0.09 | 0.06 |  |
|  |  |  |  |  | ADB-BUTINACA | 282030 | 4.10 | 8.28 | 99.14 |
|  |  |  |  |  | ADB-4en-PINACA | 498604 | 9.08 | 57.95 |  |
|  |  |  |  |  | ADB-INACA | 12465 | 0.18 | 0.57 |  |
| 30 | GA | Paper | N/A | 2023-11-10 | MDMB-4en-PINACA | 4635682 | 71.82 | 6.83 | 99.36 |
|  |  |  |  |  | 4F-MDMB-BUTINACA | 128332 | 6.22 | 0.58 |  |
|  |  |  |  |  | MDMB-INACA | 18091 | 0.28 | 0.05 |  |
|  |  |  |  |  | ADB-BUTINACA | 502705 | 7.79 | 4.00 | 99.29 |
|  |  |  |  |  | ADB-4en-PINACA | 1116722 | 54.15 | 87.88 |  |
|  |  |  |  |  | ADB-INACA | 52853 | 0.82 | 0.66 |  |
| 31 | GA | Paper | N/A | 2023-11-10 | MDMB-4en-PINACA | 243739 | 98.56 | 97.44 | 97.44 |
|  |  |  |  |  | MDMB-INACA | 3572 | 1.44 | 2.56 |  |
| 32 | GA | Paper | N/A | 2023-11-10 | MDMB-4en-PINACA | 2966686 | 63.05 | 23.76 | 99.26 |
|  |  |  |  |  | MDMB-INACA | 12346 | 0.26 | 0.18 |  |
|  |  |  |  |  | ADB-BUTINACA | 1717907 | 36.51 | 74.39 | 99.81 |
|  |  |  |  |  | ADB-4en-PINACA | 6217 | 0.24 | 1.52 |  |
|  |  |  |  |  | ADB-INACA | 2148 | 0.05 | 0.15 |  |
| 33 | GA | Paper | N/A | 2023-11-10 | MDMB-4en-PINACA | 891785 | 92.26 | 86.92 | 86.92 |
|  |  |  |  |  | MDMB-INACA | 74779 | 7.74 | 13.08 |  |
| 34 | GA | Paper | N/A | 2023-11-10 | MDMB-4en-PINACA | 843678 | 90.91 | 84.79 | 84.79 |
|  |  |  |  |  | MDMB-INACA | 84344 | 9.09 | 15.21 |  |

**SECTION 7**

Example examination photographs of samples seized from the Scottish prisons

**(A)**


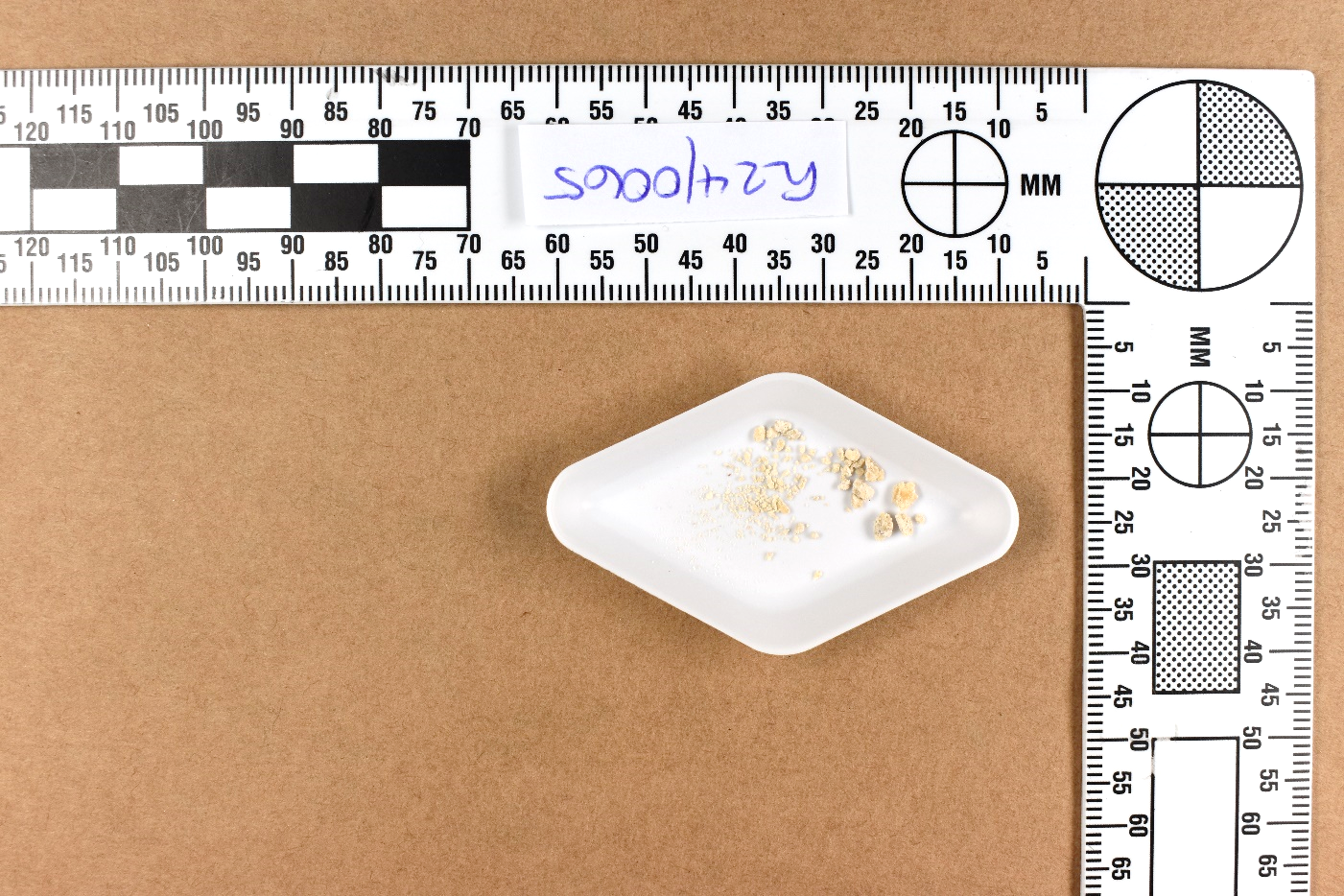

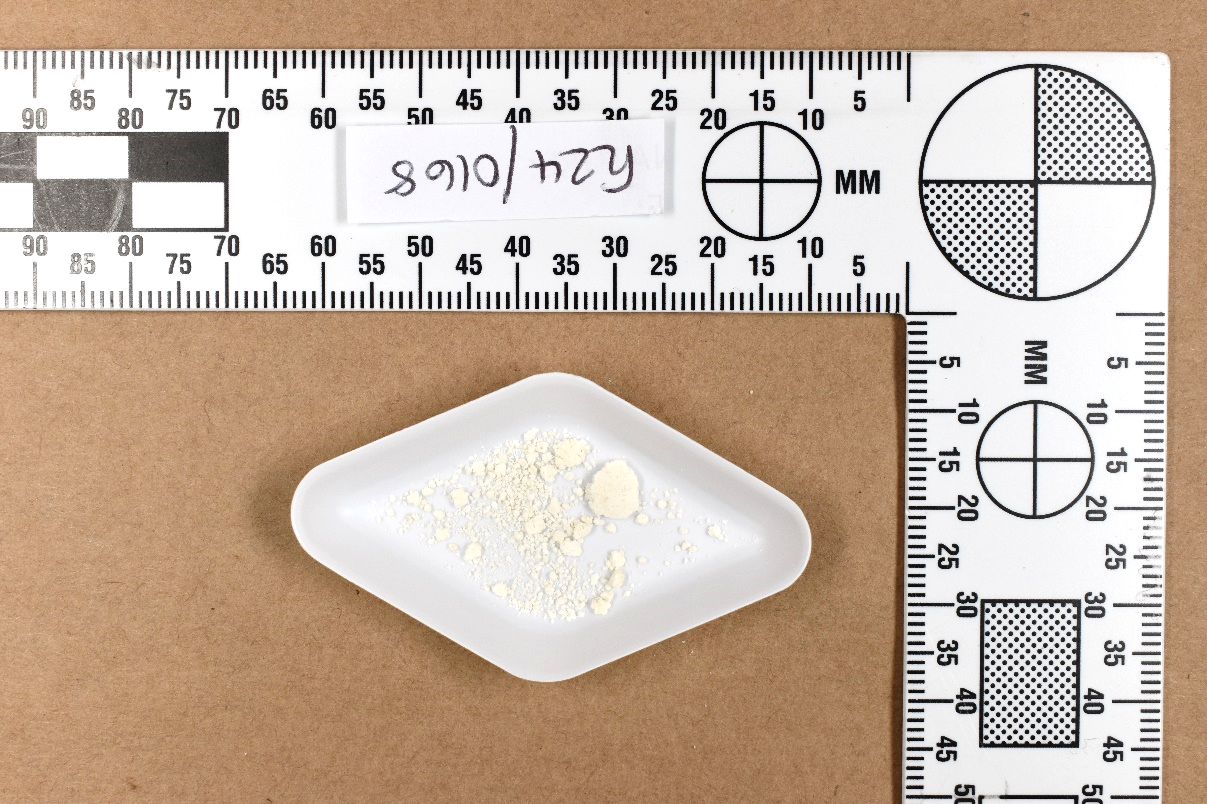


**(B)**

**Figure S7.1.** Example examination photographs of powders seized from the Scottish prisons. (A) FL24/0065 seized 1^st^ April 2024 and found positive for MDMB-INACA and MDMB-4en-PINACA. Note that this powder seems to have a waxy consistency. (B) FL24/0168 seized 22^nd^ July 2024 and found positive for MDMB-INACA and MDMB-FUBINACA.

**(A)**

**(B)**


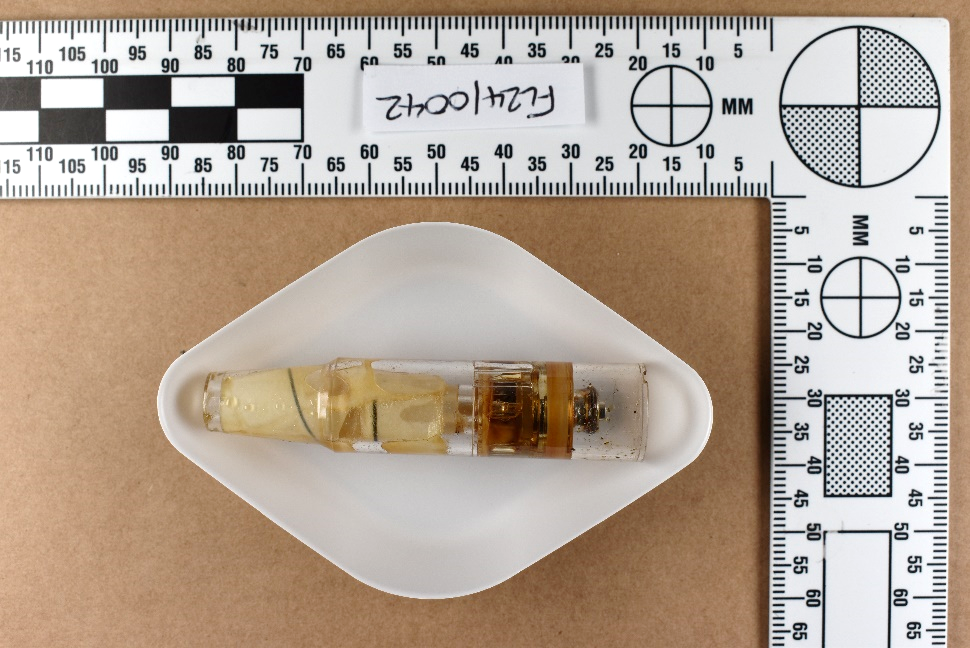

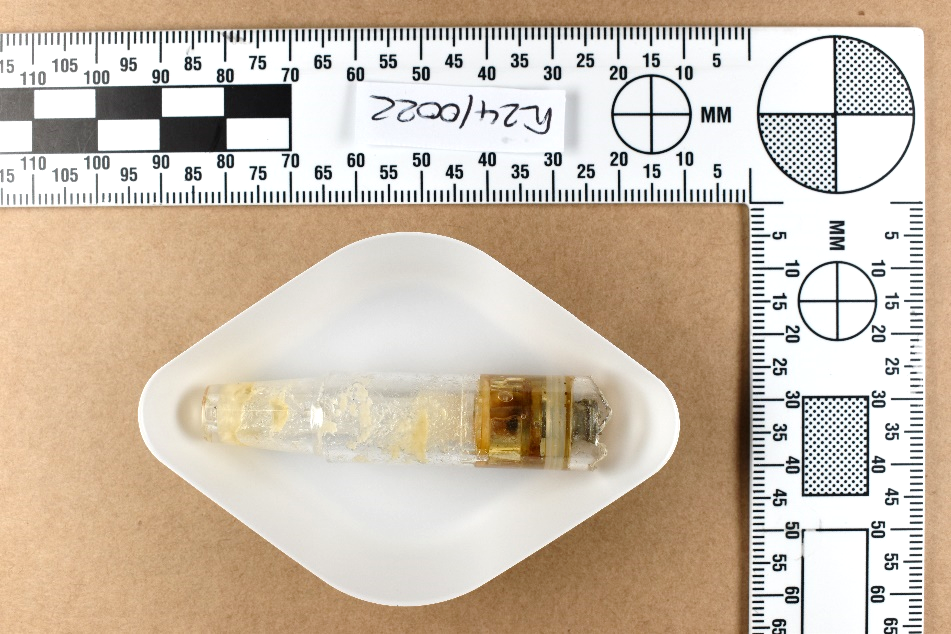


**Figure S7.2.** Example examination photographs of e-cigarette cartridges seized from the Scottish prisons. (A) FL24/0042 seized 21^st^ November 2023 and found positive for MDMB-INACA, MDMB-4en-PINACA, ADB-BUTINACA, and nicotine. Note the folded paper inside the cartridge. (B) FL24/0022 seized 1^st^ April 2024 and found positive for MDMB-INACA, MDMB-4en-PINACA, and nicotine. Note powder or waxy material inside the cartridge.


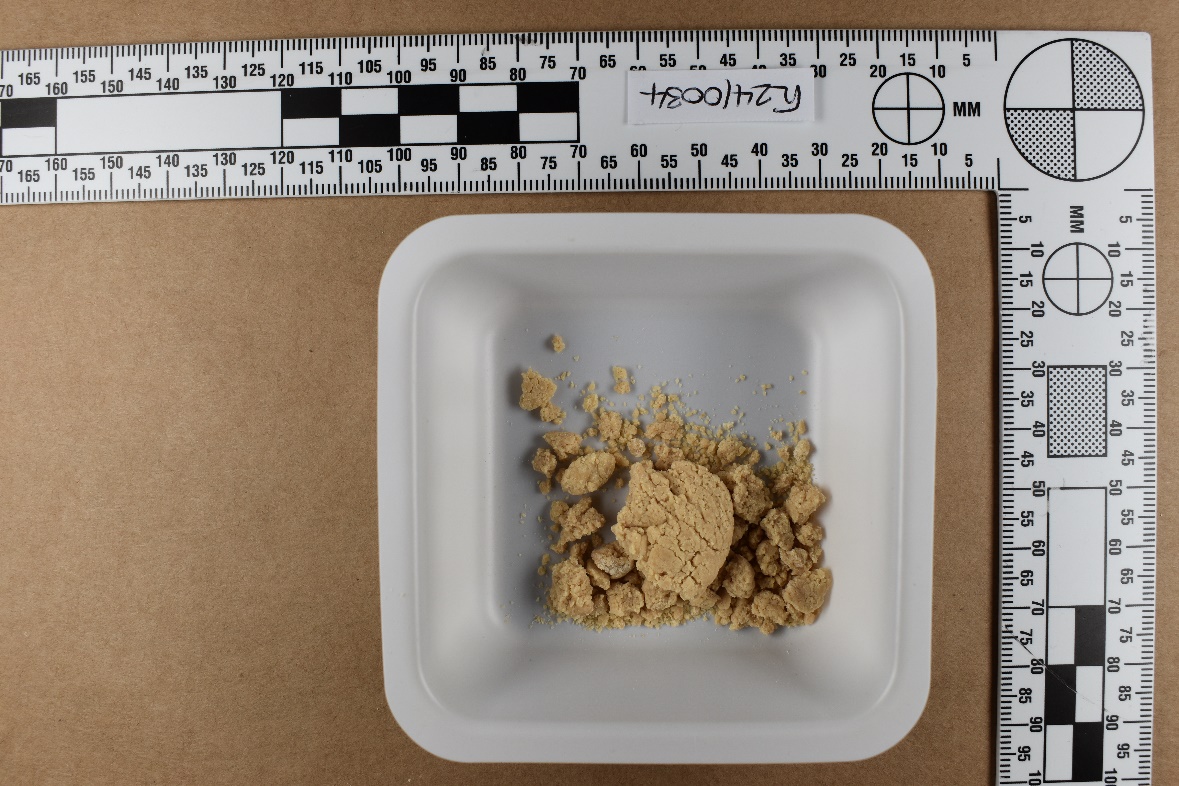

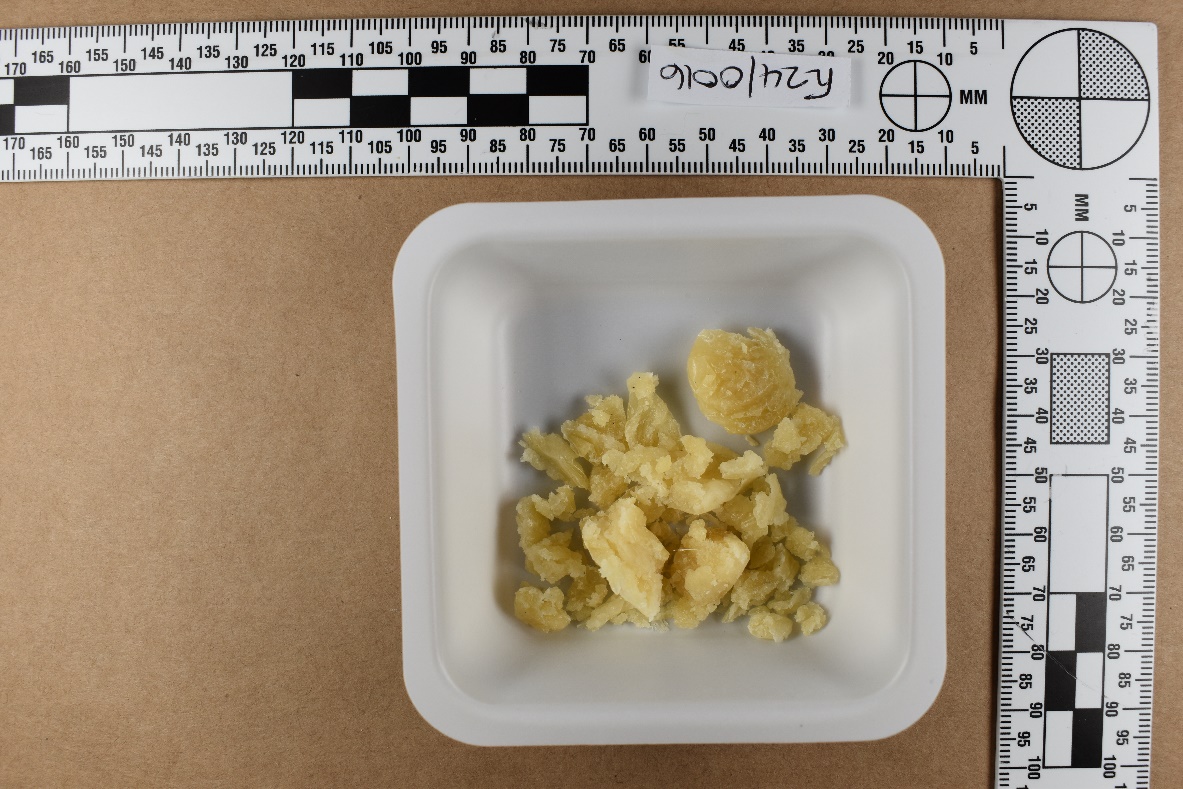


**(B)**

**(A)**

**Figure S7.3.** Example examination photographs of waxy- or putty-like materials seized from the Scottish prisons. More examples can be found in Timmermann et al 2024 (<https://doi.org/10.1002/dta.3817>). (A) FL24/0034 seized 9^th^ November 2023 and found positive for MDMB-INACA, MDMB-4en-PINACA, and MDMB-5’Me-INACA. (B) FL24/0016 seized 24^th^ January 2024 and found positive for AB-INACA and AB-CHMINACA.


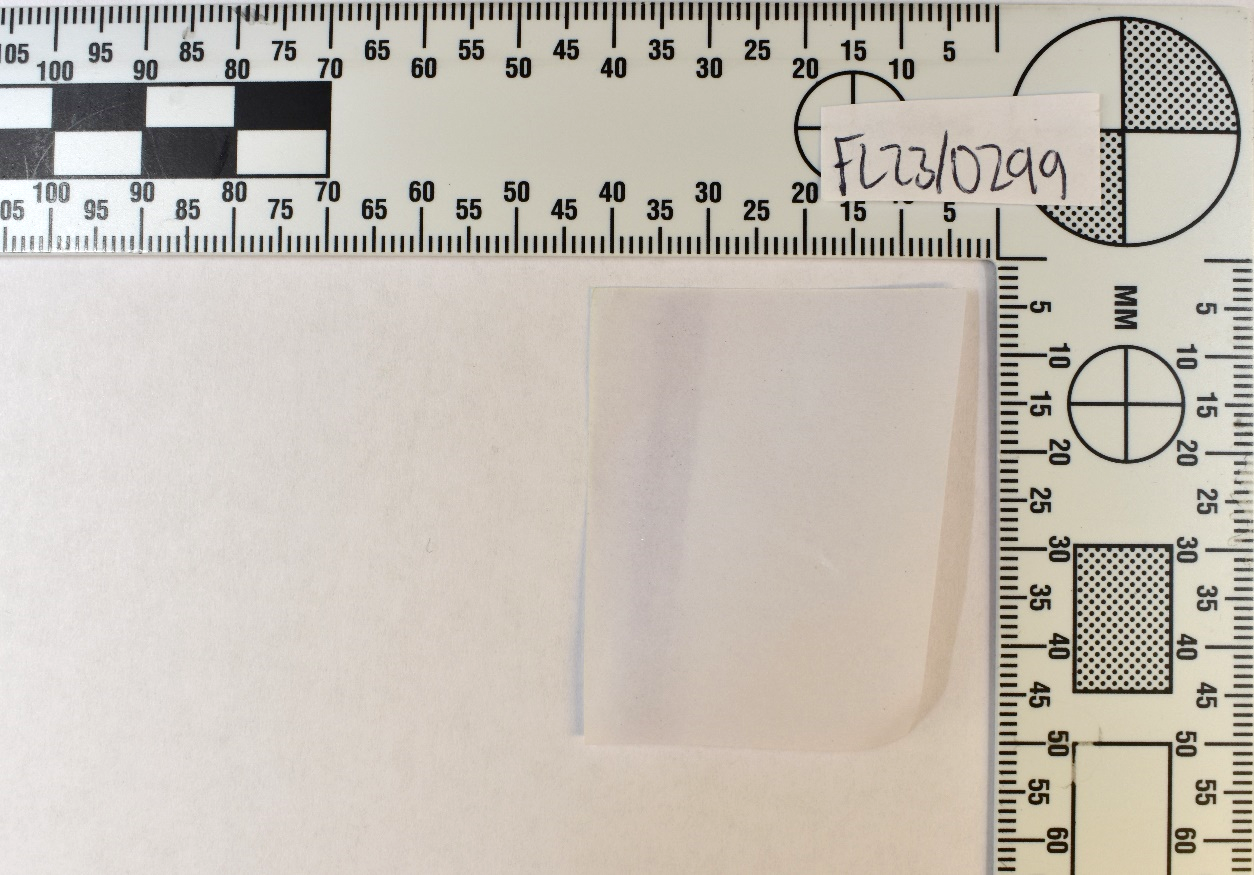

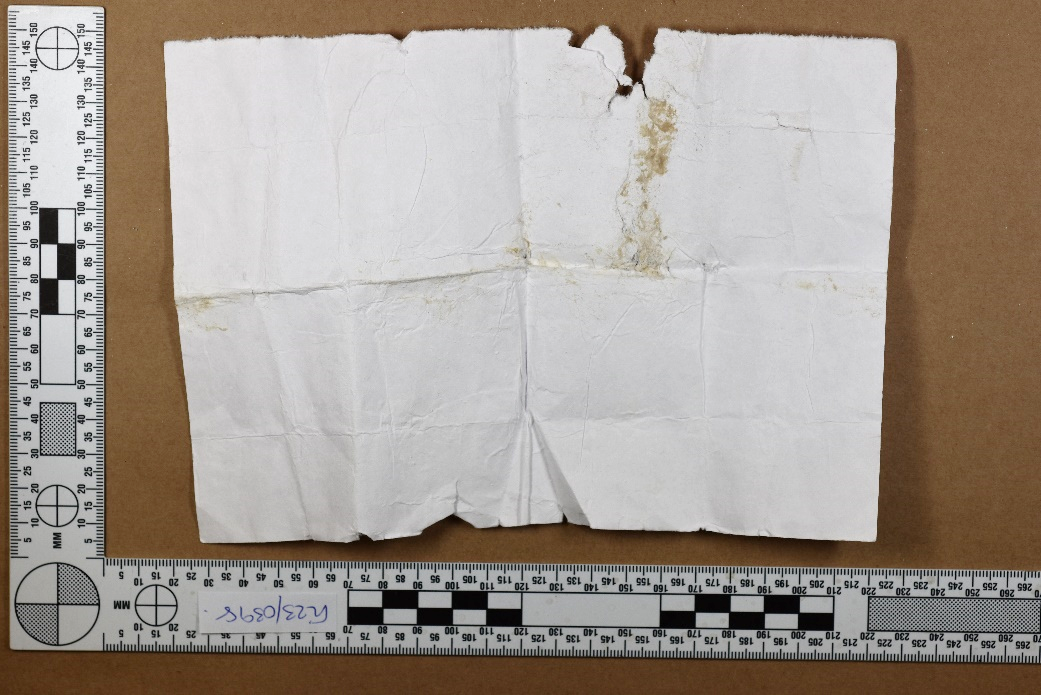


**(B)**

**(A)**

**Figure S7.4.** Example examination photographs of papers seized from the Scottish prisons. (A) FL23/0299 seized 25^th^ July 2023 and found positive for MDMB-INACA, MDMB-4en-PINACA, and ADB-BUTINACA. (B) FL23/0398 seized 1^st^ November 2023 and found positive for MDMB-INACA, MDMB-5’Br-INACA, and ADB-BUTINACA.


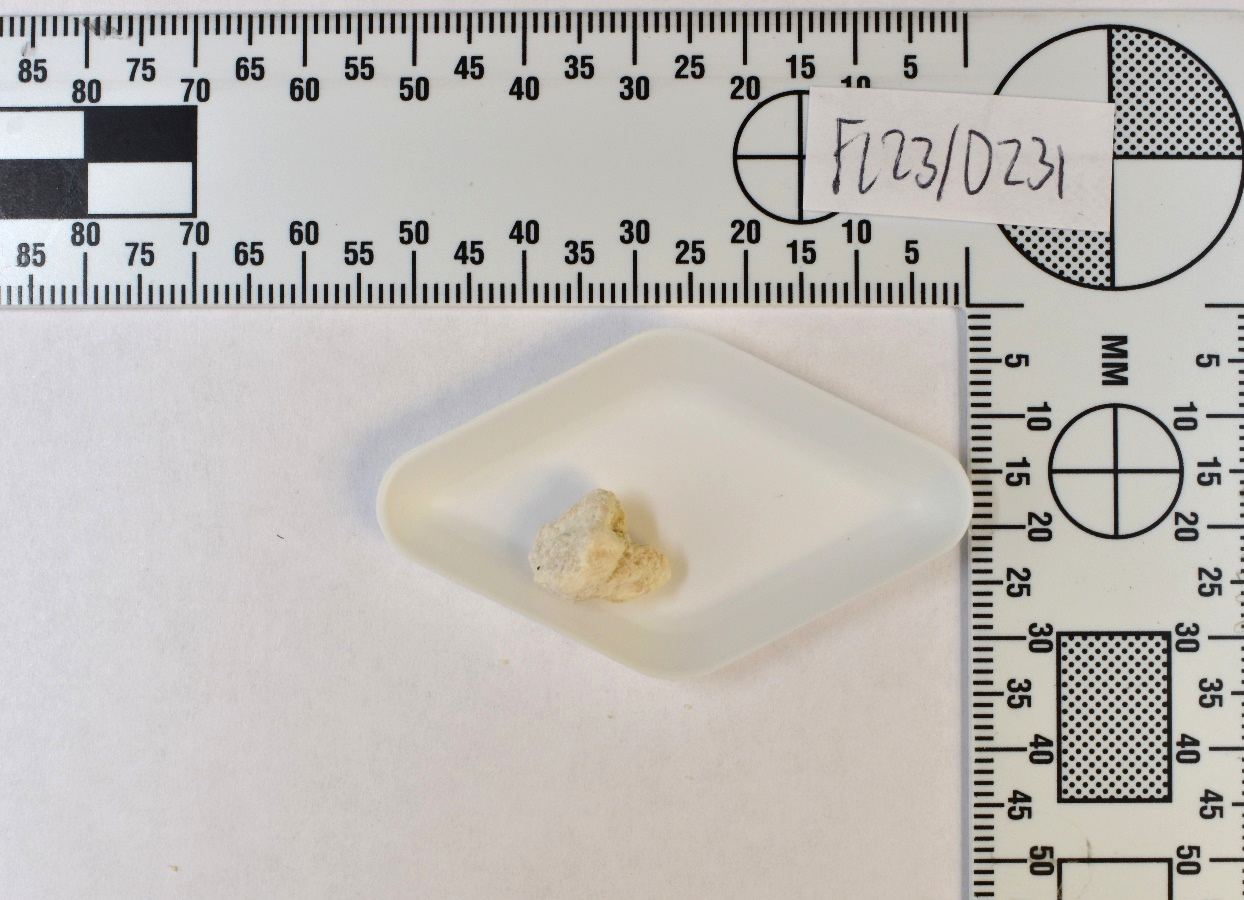

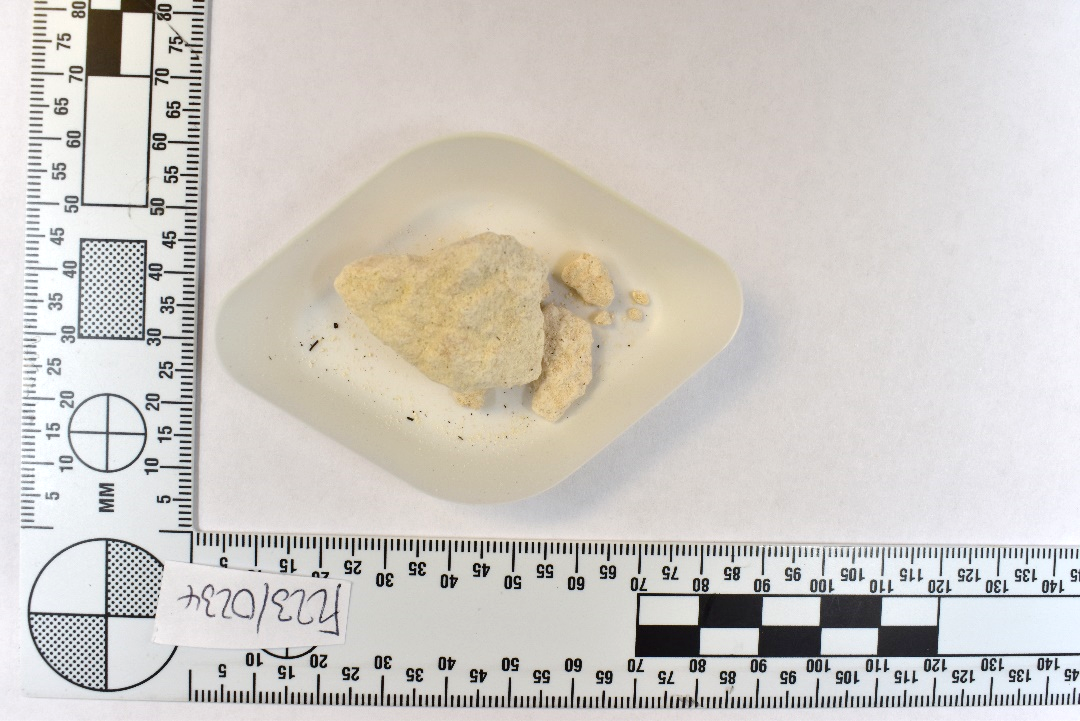


**(B)**

**(A)**

**Figure S7.5.** Examination photographs of the solid powder rock-like samples seized on 22^nd^ March 2023 from the Scottish prisons and found to contain MDMB-INACA and MDMB-4en-PINACA: (A) FL23/0231 and (B) FL23/0234.

**SECTION 8**

Complete data for the samples seized from the Scottish prisons found positive for a tail-less SCRA/precursor with one or two corresponding SCRA(s)

**Table S8.1.** Complete data for samples seized from Scottish prisons found positive for a mixture of a tail-less SCRA/precursor with one or two corresponding SCRA(s). Samples are organized by seizure date. For security reasons, the prison where the sample was seized is represented by a number rather than the name of the establishment. Details of the GC–MS analysis are provided, including retention time (RT) of the compound, RT of the reference standard (ref std), and the mass (m/z) of the compound. The percentage of the peak areas of the SCRAs are provided and have been corrected for EI-MS detector response using the corrections found in Table S2.2.

| **Sample ID** | **Prison** | **Seizure Date** | **Sample Type** | **Analysis Result** | **RT (mins)** | **R-Match (x/1000)** | **RT Ref Std (mins)** | **m/z (amu)** | **% Peak Area** | **% Final of precursor** |
| --- | --- | --- | --- | --- | --- | --- | --- | --- | --- | --- |
| FL23/0174 | 3 | 13-03-2023 | Waxy material | ADB-BUTINACA | 9.318 | 881 | 9.204 | 330 | 2.55% | - |
|  |  |  |  | MDMB-4en-PINACA | 8.969 | 941 | 8.900 | 357 | 18.84% | 19.33% |
|  |  |  |  | MDMB-INACA | 8.746 | 891 | 8.666 | 289 | 78.61% |  |
| FL23/0315 | 5 | 15-03-2023 | E-cigarette | MDMB-4en-PINACA | 8.835 | 940 | 8.836 | 357 | 44.24% | 44.24% |
|  |  |  |  | MDMB-INACA | 8.628 | 861 | 8.592 | 289 | 55.76% |  |
|  |  |  |  | Nicotine | 5.528 | 928 | - | 162 | - | - |
| FL23/0212 | 6 | 18-03-2023 | Powder | MDMB-4en-PINACA | 7.488 | 938 | 7.499 | 357 | 18.62% | 18.62% |
|  |  |  |  | MDMB-INACA | 8.682 | 878 | 8.666 | 289 | 81.38% |  |
| FL23/0211 | 6 | 20-03-2023 | Powder | MDMB-4en-PINACA | 7.485 | 943 | 7.499 | 357 | 17.66% | 17.66% |
|  |  |  |  | MDMB-INACA | 8.683 | 878 | 8.666 | 289 | 82.34% |  |
| FL23/0231 | 3 | 22-03-2023 | Solid | MDMB-4en-PINACA | 8.933 | 940 | 8.898 | 357 | 17.68% | 17.68% |
|  |  |  |  | MDMB-INACA | 8.754 | 864 | 8.666 | 289 | 82.32% |  |
| FL23/0234 | 3 | 22-03-2023 | Solid | MDMB-4en-PINACA | 8.954 | 936 | 8.900 | 357 | 15.72% | 15.72% |
|  |  |  |  | MDMB-INACA | 8.740 | 864 | 8.666 | 289 | 84.28% |  |
| FL23/0217 | 6 | 28-03-2023 | Powder | MDMB-4en-PINACA | 7.489 | 934 | 7.497 | 357 | 24.43% | 24.43% |
|  |  |  |  | MDMB-INACA | 8.667 | 883 | 8.666 | 289 | 75.57% |  |
|  |  |  |  | Amitriptyline | 6.352 | 949 | - | 277 | - | - |
|  |  |  |  | Clonazolam | 12.904 | 889 | 12.879 | 353 | - | - |
| FL23/0228 | 3 | 28-03-2023 | E-cigarette | MDMB-4en-PINACA | 8.895 | 910 | 8.900 | 357 | 60.14% | 60.14% |
|  |  |  |  | MDMB-INACA | 8.654 | 891 | 8.666 | 289 | 39.86% |  |
| FL23/0220-1 | 6 | 31-03-2023 | Powder | MDMB-4en-PINACA | 7.536 | 938 | 7.497 | 357 | 17.34% | 17.34% |
|  |  |  |  | MDMB-INACA | 8.690 | 864 | 8.666 | 289 | 82.66% |  |
| FL23/0220-2 | 6 | 31-03-2023 | Powder | MDMB-4en-PINACA | 7.546 | 936 | 7.497 | 357 | 16.37% | 16.37% |
|  |  |  |  | MDMB-INACA | 8.678 | 880 | 8.666 | 289 | 83.63% |  |
| FL23/0135 | 11 | 03-04-2023 | Waxy material | ADB-BUTINACA | 9.215 | 875 | 9.209 | 330 | 92.80% | - |
|  |  |  |  | MDMB-4en-PINACA | 8.900 | 926 | 8.906 | 357 | 3.91% | 54.22% |
|  |  |  |  | MDMB-INACA | 8.717 | 882 | 8.666 | 289 | 3.30% |  |
| FL23/0199 | 3 | 11-04-2023 | Waxy material | ADB-BUTINACA | 9.247 | 890 | 9.193 | 330 | 5.62% | - |
|  |  |  |  | MDMB-4en-PINACA | 8.921 | 937 | 8.900 | 357 | 12.87% | 13.64% |
|  |  |  |  | MDMB-INACA | 8.717 | 876 | 8.666 | 289 | 81.51% |  |
| FL23/0104 | 1 | 13-04-2023 | Powder | MDMB-4en-PINACA | 8.964 | 942 | 8.903 | 357 | 17.11% | 17.11% |
|  |  |  |  | MDMB-INACA | 8.734 | 886 | 8.666 | 289 | 82.89% |  |
|  |  |  |  | ADB-4en-PINACA | 9.785 | 892 | 9.459 | 342 | - | - |
|  |  |  |  | ADB-5'Br-BUTINACA | 10.452 | - | 10.423 | 409 | - | - |
| FL23/0191 | 1 | 20-04-2023 | Powder | MDMB-4en-PINACA | 8.975 | 940 | 8.900 | 357 | 13.16% | 13.16% |
|  |  |  |  | MDMB-INACA | 8.675 | 869 | 8.666 | 289 | 86.84% |  |
| FL23/0192 | 1 | 21-04-2023 | E-cigarette | MDMB-4en-PINACA | 8.975 | 938 | 8.900 | 357 | 14.09% | 14.09% |
|  |  |  |  | MDMB-INACA | 8.668 | 870 | 8.666 | 289 | 85.91% |  |
| FL23/0325 | 6 | 23-04-2023 | Powder | MDMB-4en-PINACA | 8.872 | 940 | 8.836 | 357 | 19.01% | 19.01% |
|  |  |  |  | MDMB-INACA | 8.685 | 854 | 8.602 | 289 | 80.99% |  |
| FL23/0197 | 3 | 24-04-2023 | E-cigarette | MDMB-4en-PINACA | 8.887 | 893 | 8.898 | 357 | 9.77% | 9.77% |
|  |  |  |  | MDMB-INACA | 8.654 | 894 | 8.666 | 289 | 90.23% |  |
| FL23/0437 | 11 | 25-04-2023 | Paper | MDMB-4en-PINACA | 8.760 | 943 | 8.752 | 357 | 20.59% | 61.83% |
|  |  |  |  | MDMB-INACA | 8.551 | 891 | 8.530 | 289 | 33.09% |  |
|  |  |  |  | MDMB-BUTINACA | 8.584 | 931 | - | 345 | 33.01% |  |
|  |  |  |  | ADB-BUTINACA | 9.036 | 876 | 9.026 | 330 | 13.32% | - |
| FL23/0224 | 6 | 28-04-2023 | Powder | MDMB-4en-PINACA | 8.954 | 939 | 8.900 | 357 | 16.18% | 16.18% |
|  |  |  |  | MDMB-INACA | 8.740 | 890 | 8.666 | 289 | 83.82% |  |
| FL23/0193-3 | 3 | 03-05-2023 | E-cigarette | MDMB-4en-PINACA | 8.888 | 916 | 8.900 | 357 | 63.42% | 63.42% |
|  |  |  |  | MDMB-INACA | 8.649 | 851 | 8.666 | 289 | 36.58% |  |
| FL23/0193-4 | 3 | 03-05-2023 | E-cigarette | MDMB-4en-PINACA | 8.888 | 910 | 8.900 | 357 | 42.29% | 42.29% |
|  |  |  |  | MDMB-INACA | 8.653 | 872 | 8.666 | 289 | 57.71% |  |
| FL23/0349 | 7 | 14-05-2023 | E-cigarette | MDMB-4en-PINACA | 8.832 | 904 | 8.836 | 357 | 60.27% | 60.27% |
|  |  |  |  | MDMB-INACA | 8.601 | 861 | 8.602 | 289 | 39.73% |  |
|  |  |  |  | Nicotine | 5.462 | 917 | - | 162 | - | - |
| FL23/0188 | 1 | 15-05-2023 | Waxy material | ADB-BUTINACA | 9.267 | 885 | 9.204 | 330 | 1.10% | - |
|  |  |  |  | MDMB-4en-PINACA | 8.969 | 937 | 8.900 | 357 | 46.66% | 47.18% |
|  |  |  |  | MDMB-INACA | 8.746 | 879 | 8.666 | 289 | 52.24% |  |
| FL23/0273 | 4 | 15-05-2023 | Paper | MDMB-4en-PINACA | 8.902 | 937 | 8.886 | 357 | 46.37% | 46.37% |
|  |  |  |  | MDMB-INACA | 8.689 | 880 | 8.637 | 289 | 53.63% |  |
| FL23/0348-1 | 7 | 23-05-2023 | E-cigarette | MDMB-4en-PINACA | 8.845 | 934 | 8.836 | 357 | 2.93% | 2.93% |
|  |  |  |  | MDMB-INACA | 8.664 | 906 | 8.602 | 289 | 97.07% |  |
|  |  |  |  | Nicotine | 5.509 | 922 | - | 162 | - | - |
| FL23/0348-2 | 7 | 23-05-2023 | E-cigarette | MDMB-4en-PINACA | 8.831 | 907 | 8.836 | 357 | 3.45% | 12.11% |
|  |  |  |  | MDMB-INACA | 8.616 | 904 | 8.602 | 289 | 87.89% |  |
|  |  |  |  | MDMB-BUTINACA | 8.831 | 907 | - | 345 | 8.66% |  |
|  |  |  |  | Nicotine | 5.474 | 883 | - | 162 | - | - |
| FL23/0439 | 11 | 27-05-2023 | E-cigarette | MDMB-4en-PINACA | 8.750 | 919 | 8.757 | 357 | 26.77% | 39.93% |
|  |  |  |  | MDMB-INACA | 8.530 | 899 | 8.537 | 289 | 40.27% |  |
|  |  |  |  | ADB-BUTINACA | 9.021 | 889 | 9.032 | 330 | 32.96% | - |
|  |  |  |  | Nicotine | 5.367 | 955 | - | 162 | - | - |
| FL23/0386 | 9 | 13-06-2023 | Powder | MDMB-4en-PINACA | 8.764 | 940 | 8.750 | 357 | 29.05% | 29.05% |
|  |  |  |  | MDMB-INACA | 8.555 | 889 | 8.531 | 289 | 70.95% |  |
| FL23/0347-2 | 7 | 21-06-2023 | E-cigarette | MDMB-4en-PINACA | 8.840 | 928 | 8.836 | 357 | 67.82% | 69.28% |
|  |  |  |  | MDMB-INACA | 8.613 | 874 | 8.602 | 289 | 30.08% |  |
|  |  |  |  | ADB-BUTINACA | 9.121 | 896 | 9.128 | 330 | 2.10% | - |
| FL23/0347-3 | 7 | 21-06-2023 | E-cigarette | MDMB-4en-PINACA | 8.880 | 946 | 8.836 | 357 | 13.52% | 13.52% |
|  |  |  |  | MDMB-INACA | 8.715 | 870 | 8.602 | 289 | 86.48% |  |
| FL23/0312 | 5 | 01-07-2023 | E-cigarette | MDMB-4en-PINACA | 8.185 | 916 | 8.826 | 357 | 23.22% | 23.22% |
|  |  |  |  | MDMB-INACA | 8.590 | 875 | 8.592 | 289 | 76.78% |  |
|  |  |  |  | Nicotine | 5.453 | 909 | 5.460 | 162 | - | - |
| FL23/0448 | 11 | 06-07-2023 | E-cigarette | MDMB-4en-PINACA | 8.750 | 904 | 8.757 | 357 | 17.26% | 20.83% |
|  |  |  |  | MDMB-INACA | 8.532 | 894 | 8.537 | 289 | 65.59% |  |
|  |  |  |  | ADB-BUTINACA | 9.024 | 860 | 9.032 | 330 | 17.16% | - |
| FL23/0326-1 | 6 | 10-07-2023 | E-cigarette | MDMB-4en-PINACA | 8.854 | 939 | 8.829 | 357 | 32.13% | 32.13% |
|  |  |  |  | MDMB-INACA | 8.653 | 851 | 8.601 | 289 | 67.87% |  |
|  |  |  |  | Nicotine | 5.458 | 951 | - | 162 | - | - |
|  |  |  |  | Quetiapine | 11.556 | 916 | - | 383 | - | - |
| FL23/0326-5 | 6 | 10-07-2023 | Powder | MDMB-4en-PINACA | 8.844 | 939 | 8.839 | 357 | 32.13% | 32.13% |
|  |  |  |  | MDMB-INACA | 8.641 | 859 | 8.601 | 289 | 67.87% |  |
| FL23/0311 | 5 | 11-07-2023 | E-cigarette | ADB-BUTINACA | 9.101 | 902 | 9.132 | 330 | 1.68% | - |
|  |  |  |  | MDMB-4en-PINACA | 8.818 | 925 | 8.836 | 357 | 2.98% | 54.43% |
|  |  |  |  | MDMB-INACA | 8.589 | 876 | 8.592 | 289 | 2.49% |  |
|  |  |  |  | Bromazolam | 10.646 | 973 | 10.645 | 352 | 92.85% | - |
|  |  |  |  | Nicotine | 5.442 | 954 | 5.460 | 162 | - | - |
| FL23/0296 | 1 | 12-07-2023 | Paper | MDMB-4en-PINACA | 8.852 | 934 | 8.849 | 357 | 37.68% | 71.14% |
|  |  |  |  | MDMB-INACA | 8.621 | 899 | 8.618 | 289 | 15.29% |  |
|  |  |  |  | ADB-BUTINACA | 9.153 | 885 | 9.142 | 330 | 47.03% | - |
| FL23/0314 | 5 | 13-07-2023 | Powder | MDMB-4en-PINACA | 8.830 | 943 | 8.823 | 357 | 16.95% | 16.95% |
|  |  |  |  | MDMB-INACA | 8.637 | 943 | 8.584 | 289 | 83.05% |  |
| FL23/0327-1 | 6 | 14-07-2023 | Powder | MDMB-4en-PINACA | 7.442 | 938 | 7.400 | 357 | 5.92% | 5.92% |
|  |  |  |  | MDMB-INACA | 7.156 | 886 | 7.040 | 289 | 94.08% |  |
|  |  |  |  | Dimethylpentylone | 5.202 | 934 | - | 249 | - | - |
| FL23/0327-4 | 6 | 14-07-2023 | Powder | MDMB-4en-PINACA | 7.442 | 940 | 7.400 | 357 | 3.95% | 3.95% |
|  |  |  |  | MDMB-INACA | 7.163 | 874 | 7.040 | 289 | 96.05% |  |
|  |  |  |  | Dimethylpentylone | 5.199 | 940 | - | 249 | - | - |
| FL23/0343-1 | 7 | 24-07-2023 | Paper | MDMB-4en-PINACA | 8.828 | 884 | 8.837 | 357 | 15.12% | 80.10% |
|  |  |  |  | MDMB-INACA | 8.599 | 857 | 8.601 | 289 | 19.90% |  |
|  |  |  |  | MDMB-BUTINACA | 8.645 | 923 | - | 345 | 64.99% |  |
|  |  |  |  | Nicotine | 5.449 | 964 | - | 162 | - | - |
| FL23/0343-2 | 7 | 24-07-2023 | E-cigarette | MDMB-INACA | 8.599 | 855 | 8.601 | 289 | 82.76% | 17.24% |
|  |  |  |  | MDMB-BUTINACA | 8.644 | 851 | - | 345 | 17.24% |  |
|  |  |  |  | Nicotine | 5.457 | 960 | - | 162 | - | - |
| FL23/0343-3 | 7 | 24-07-2023 | E-cigarette | MDMB-4en-PINACA | 8.827 | 927 | 8.837 | 357 | 17.67% | 58.69% |
|  |  |  |  | MDMB-INACA | 8.603 | 886 | 8.601 | 289 | 41.31% |  |
|  |  |  |  | MDMB-BUTINACA | 8.645 | 926 | - | 345 | 41.01% |  |
|  |  |  |  | Nicotine | 5.478 | 885 | - | 162 | - | - |
| FL23/0444 | 11 | 24-07-2023 | E-cigarette | MDMB-4en-PINACA | 8.750 | 930 | 8.757 | 289 | 13.21% | 20.69% |
|  |  |  |  | MDMB-INACA | 8.537 | 880 | 8.537 | 358 | 50.64% |  |
|  |  |  |  | ADB-BUTINACA | 9.032 | 898 | 9.032 | 330 | 36.15% | - |
|  |  |  |  | Nicotine | 5.405 | 921 | - | 162 | - | - |
| FL23/0445 | 11 | 24-07-2023 | Waxy material | MDMB-4en-PINACA | 8.765 | 943 | 8.752 | 357 | 10.69% | 12.40% |
|  |  |  |  | MDMB-INACA | 8.575 | 884 | 8.532 | 289 | 75.56% |  |
|  |  |  |  | ADB-BUTINACA | 9.056 | 827 | 9.026 | 330 | 13.74% | - |
|  |  |  |  | Nicotine | 5.369 | 926 | - | 162 | - | - |
| FL23/0299 | 1 | 25-07-2023 | Paper | MDMB-4en-PINACA | 8.875 | 942 | 8.849 | 357 | 42.82% | 42.82% |
|  |  |  |  | MDMB-INACA | 8.664 | 875 | 8.618 | 289 | 57.18% |  |
|  |  |  |  | ADB-BUTINACA | 9.159 | 903 | 9.142 | 330 | - | - |
| FL23/0303-1 | 4 | 28-07-2023 | E-cigarette | MDMB-4en-PINACA | 8.842 | 926 | 8.849 | 357 | 89.77% | 89.77% |
|  |  |  |  | MDMB-INACA | 8.611 | 868 | 8.618 | 289 | 10.23% |  |
|  |  |  |  | Nicotine | 5.460 | 963 | - | 162 | - | - |
| FL23/0295 | 1 | 31-07-2023 | Waxy material | MDMB-4en-PINACA | 8.843 | 936 | 8.826 | 357 | 27.78% | 27.78% |
|  |  |  |  | MDMB-INACA | 8.692 | 862 | 8.592 | 289 | 72.22% |  |
|  |  |  |  | ADB-BUTINACA | 9.138 | 898 | 9.119 | 330 | - | - |
| FL23/0422 | 7 | 08-08-2023 | E-cigarette | MDMB-4en-PINACA | 8.772 | 935 | 8.757 | 357 | 83.13% | 83.13% |
|  |  |  |  | MDMB-INACA | 8.605 | 874 | 8.537 | 289 | 16.87% |  |
| FL23/0423-1 | 7 | 08-08-2023 | E-cigarette | MDMB-4en-PINACA | 8.740 | 876 | 8.743 | 357 | 1.92% | 1.92% |
|  |  |  |  | MDMB-INACA | 8.527 | 867 | 8.531 | 289 | 98.08% |  |
|  |  |  |  | Nicotine | 5.368 | 945 | - | 162 | - | - |
| FL23/0423-2 | 7 | 08-08-2023 | E-cigarette | MDMB-4en-PINACA | 8.750 | 904 | 8.743 | 357 | 23.94% | 23.94% |
|  |  |  |  | MDMB-INACA | 8.534 | 861 | 8.531 | 289 | 76.06% |  |
|  |  |  |  | Nicotine | 5.366 | 950 | - | 162 | - | - |
| FL23/0393-1 | 11 | 14-08-2023 | E-cigarette | MDMB-4en-PINACA | 8.740 | 922 | 8.743 | 357 | 22.85% | 77.48% |
|  |  |  |  | MDMB-INACA | 8.529 | 869 | 8.531 | 289 | 6.64% |  |
|  |  |  |  | ADB-BUTINACA | 9.015 | 902 | 9.018 | 330 | 70.50% | - |
|  |  |  |  | Nicotine | 5.363 | 953 | - | 162 | - | - |
| FL23/0393-2 | 11 | 14-08-2023 | E-cigarette | MDMB-4en-PINACA | 8.739 | 927 | 8.743 | 357 | 76.49% | 85.23% |
|  |  |  |  | MDMB-INACA | 8.528 | 863 | 8.531 | 289 | 13.26% |  |
|  |  |  |  | ADB-BUTINACA | 9.015 | 906 | 9.018 | 330 | 10.25% | - |
|  |  |  |  | Nicotine | 5.362 | 949 | - | 162 | - | - |
| FL23/0301 | 1 | 15-08-2023 | Waxy material | ADB-BUTINACA | 9.229 | 886 | 9.142 | 330 | 54.79% | - |
|  |  |  |  | MDMB-4en-PINACA | 8.872 | 939 | 8.849 | 357 | 13.38% | 29.59% |
|  |  |  |  | MDMB-INACA | 8.675 | 863 | 8.618 | 289 | 31.83% |  |
| FL23/0302 | 1 | 15-08-2023 | Waxy material | ADB-BUTINACA | 9.191 | 860 | 9.142 | 330 | 15.64% | - |
|  |  |  |  | MDMB-4en-PINACA | 8.868 | 938 | 8.849 | 357 | 14.06% | 16.67% |
|  |  |  |  | MDMB-INACA | 8.686 | 872 | 8.618 | 289 | 70.30% |  |
| FL23/0431-2 | 7 | 18-08-2023 | E-cigarette | MDMB-4en-PINACA | 8.755 | 931 | 8.752 | 357 | 95.92% | 95.92% |
|  |  |  |  | MDMB-INACA | 8.534 | 866 | 8.532 | 289 | 4.08% |  |
|  |  |  |  | Nicotine | 5.375 | 961 | - | 162 | - | - |
| FL24/0001 | 10 | 18-08-2023 | E-cigarette | MDMB-4en-PINACA | 9.348 | 929 | 9.347 | 357 | 94.15% | 94.15% |
|  |  |  |  | MDMB-INACA | 9.055 | 863 | 9.061 | 289 | 5.85% |  |
| FL23/0392-1 | 11 | 24-08-2023 | E-cigarette | MDMB-4en-PINACA | 8.737 | 929 | 8.743 | 357 | 71.13% | 71.13% |
|  |  |  |  | MDMB-INACA | 8.529 | 846 | 8.531 | 289 | 28.87% |  |
|  |  |  |  | Nicotine | 5.370 | 947 | - | 162 | - | - |
| FL23/0392-2 | 11 | 24-08-2023 | E-cigarette | MDMB-4en-PINACA | 8.738 | 934 | 8.743 | 357 | 49.02% | 49.02% |
|  |  |  |  | MDMB-INACA | 8.525 | 872 | 8.531 | 289 | 50.98% |  |
|  |  |  |  | Nicotine | 5.372 | 952 | - | 162 | - | - |
| FL24/0037 | 3 | 18-09-2023 | Waxy material | AB-CHMINACA | 11.533 | 900 | 11.537 | 260 | 49.95% | 49.95% |
|  |  |  |  | AB-INACA | 9.868 | 943 | - | 356 | 50.05% |  |
| FL23/0427 | 13 | 26-09-2023 | E-cigarette | MDMB-4en-PINACA | 8.751 | 904 | 8.757 | 357 | 18.67% | 18.67% |
|  |  |  |  | MDMB-INACA | 8.531 | 882 | 8.537 | 289 | 81.33% |  |
| FL23/0414 | 8 | 27-09-2023 | Powder | MDMB-4en-PINACA | 8.770 | 937 | 8.752 | 357 | 18.87% | 18.87% |
|  |  |  |  | MDMB-INACA | 8.579 | 887 | 8.532 | 289 | 81.13% |  |
| FL23/0456 | 1 | 28-09-2023 | Powder | MDMB-4en-PINACA | 8.790 | 935 | 8.570 | 357 | 29.06% | 29.06% |
|  |  |  |  | MDMB-INACA | 8.635 | 884 | 8.531 | 289 | 70.94% |  |
| FL24/0040 | 3 | 29-09-2023 | Waxy material | MDMB-4en-PINACA | 9.364 | 935 | 9.347 | 357 | 76.06% | 76.06% |
|  |  |  |  | MDMB-INACA | 9.112 | 738 | 9.061 | 289 | 23.94% |  |
|  |  |  |  | MDMB-5'Me-INACA | 8.746 | 885 | - | 303 | - | - |
| FL23/0460 | 1 | 12-10-2023 | Powder | MDMB-4en-PINACA | 8.779 | 939 | 8.757 | 330 | 38.49% | 38.49% |
|  |  |  |  | MDMB-INACA | 8.605 | 870 | 8.537 | 357 | 61.51% |  |
| FL23/0461 | 1 | 12-10-2023 | Powder | MDMB-4en-PINACA | 8.803 | 867 | 8.750 | 357 | 56.74% | 56.74% |
|  |  |  |  | MDMB-INACA | 8.599 | 867 | 8.531 | 289 | 43.26% |  |
| FL23/0397 | 11 | 29-10-2023 | Powder | MDMB-4en-PINACA | 8.770 | 937 | 8.757 | 357 | 80.84% | 80.84% |
|  |  |  |  | MDMB-INACA | 8.566 | 880 | 8.537 | 289 | 19.16% |  |
| FL24/0033 | 3 | 09-11-2023 | Powder | AB-CHMINACA | 11.530 | 887 | 11.510 | 260 | 66.32% | 66.32% |
|  |  |  |  | AB-INACA | 10.441 | 946 | - | 356 | 33.68% |  |
| FL24/0034 | 3 | 09-11-2023 | Waxy material | MDMB-4en-PINACA | 9.364 | 937 | 9.371 | 357 | 27.86% | 27.86% |
|  |  |  |  | MDMB-INACA | 9.116 | 854 | 9.082 | 289 | 72.14% |  |
|  |  |  |  | MDMB-5'Me-INACA | 8.758 | 877 | - | 303 | - | - |
| FL24/0042 | 5 | 21-11-2023 | E-cigarette | MDMB-4en-PINACA | 9.338 | 865 | 9.349 | 357 | 8.57% | 48.41% |
|  |  |  |  | MDMB-INACA | 9.043 | 851 | 9.062 | 289 | 9.13% |  |
|  |  |  |  | ADB-BUTINACA | 9.702 | 894 | 9.717 | 330 | 82.31% | - |
|  |  |  |  | Nicotine | 5.852 | 955 | - | 162 | - | - |
| FL24/0010 | 3 | 31-12-2023 | Powder | MDMB-4en-PINACA | 9.381 | 941 | 9.347 | 357 | 22.61% | 22.61% |
|  |  |  |  | MDMB-INACA | 9.152 | 871 | 9.061 | 289 | 77.39% |  |
|  |  |  |  | MDMB-5'Me-INACA | 8.757 | 858 | - | 303 | - | - |
| FL24/0013 | 3 | 17-01-2024 | Powder | MDMB-4en-PINACA | 8.773 | 942 | 8.754 | 357 | 36.24% | 36.24% |
|  |  |  |  | MDMB-INACA | 8.592 | 878 | 8.602 | 289 | 63.76% |  |
| FL24/0015 | 3 | 23-01-2024 | Waxy material | MDMB-4en-PINACA | 9.351 | 932 | 9.342 | 357 | 99.85% | 99.85% |
|  |  |  |  | MDMB-INACA | 9.043 | 857 | 9.058 | 289 | 0.15% |  |
| FL24/0016 | 3 | 24-01-2024 | Waxy material | AB-CHMINACA | 11.544 | 894 | 11.488 | 356 | 81.82% | 81.82% |
|  |  |  |  | AB-INACA | 9.999 | 927 | - | 260 | 18.18% |  |
| FL24/0171 | 3 | 13-02-2024 | Powder | MDMB-FUBINACA | 10.455 | 882 | 10.371 | 397 | 69.68% | 69.68% |
|  |  |  |  | MDMB-INACA | 9.110 | 876 | 9.060 | 289 | 30.32% |  |
|  |  |  |  | MDMB-5'Me-INACA | 8.750 | 867 | - | 303 | - | - |
| FL24/0088 | 1 | 26-02-2024 | Powder | MDMB-4en-PINACA | 8.77 | 936 | 8.751 | 357 | 27.85% | 27.85% |
|  |  |  |  | MDMB-INACA | 8.605 | 876 | 8.588 | 289 | 72.15% |  |
| FL24/0175 | 3 | 18-03-2024 | Powder | MDMB-4en-PINACA | 9.380 | 941 | 9.347 | 357 | 68.76% | 68.76% |
|  |  |  |  | MDMB-INACA | 9.131 | 863 | 9.060 | 289 | 31.24% |  |
|  |  |  |  | MDMB-5'Me-INACA | 8.748 | 882 | - | 303 | - | - |
| FL24/0022 | 4 | 01-04-2024 | E-cigarette | MDMB-4en-PINACA | 9.346 | 932 | 9.371 | 357 | 93.47% | 93.47% |
|  |  |  |  | MDMB-INACA | 9.051 | 881 | 9.082 | 289 | 6.53% |  |
|  |  |  |  | Nicotine | 5.848 | 959 | - | 162 | - | - |
| FL24/0065 | 6 | 01-04-2024 | Powder | MDMB-4en-PINACA | 8.765 | 935 | 8.751 | 357 | 35.94% | 35.94% |
|  |  |  |  | MDMB-INACA | 8.594 | 854 | 8.588 | 289 | 64.06% |  |
| FL24/0066 | 6 | 19-04-2024 | Powder | MDMB-4en-PINACA | 8.771 | 926 | 8.751 | 357 | 41.64% | 41.64% |
|  |  |  |  | MDMB-INACA | 8.610 | 872 | 8.588 | 289 | 58.36% |  |
|  |  |  |  | ADB-4en-PINACA | 9.324 | 857 | 9.284 | 342 | - | - |
| FL24/0075 | 1 | 16-04-2024 | Powder | MDMB-4en-PINACA | 8.777 | 936 | 8.751 | 357 | 18.67% | 18.67% |
|  |  |  |  | MDMB-INACA | 8.627 | 872 | 8.588 | 289 | 81.33% |  |
| FL24/0076 | 1 | 16-04-2024 | Powder | MDMB-4en-PINACA | 8.792 | 941 | 8.751 | 357 | 16.97% | 16.97% |
|  |  |  |  | MDMB-INACA | 8.628 | 876 | 8.588 | 289 | 83.03% |  |
| FL24/0174 | 3 | 24-05-2024 | Powder | MDMB-4en-PINACA | 9.375 | 941 | 9.347 | 357 | 68.39% | 68.39% |
|  |  |  |  | MDMB-INACA | 9.107 | 856 | 9.110 | 289 | 31.61% |  |
|  |  |  |  | MDMB-5'Me-INACA | 8.745 | 871 | - | 303 | - | - |
| FL24/0151 | 1 | 17-06-2024 | Powder | MDMB-4en-PINACA | 9.389 | 935 | 9.347 | 357 | 80.64% | 80.64% |
|  |  |  |  | MDMB-INACA | 9.154 | 879 | 9.060 | 289 | 19.36% |  |
|  |  |  |  | MDMB-5'Me-INACA | 8.747 | 855 | - | 303 | - | - |
| FL24/0140 | 1 | 18-06-2024 | Powder | MDMB-4en-PINACA | 8.792 | 941 | 8.751 | 357 | 30.34% | 30.34% |
|  |  |  |  | MDMB-INACA | 9.151 | 876 | 9.060 | 289 | 69.66% |  |
| FL24/0165 | 3 | 22-07-2024 | Powder | MDMB-FUBINACA | 10.443 | 877 | 10.371 | 397 | 70.99% | 72.20% |
|  |  |  |  | MDMB-4en-PINACA | 9.354 | 930 | 9.347 | 357 | 1.21% |  |
|  |  |  |  | MDMB-INACA | 9.109 | 877 | 9.060 | 289 | 27.80% |  |
|  |  |  |  | MDMB-5'Me-INACA | 8.757 | 859 | - | 303 | - | - |
| FL24/0167 | 3 | 22-07-2024 | Powder | MDMB-FUBINACA | 10.445 | 880 | 10.371 | 397 | 63.64% | 66.49% |
|  |  |  |  | MDMB-4en-PINACA | 9.349 | 939 | 9.347 | 357 | 3.60% |  |
|  |  |  |  | MDMB-INACA | 9.111 | 880 | 9.060 | 289 | 32.76% |  |
|  |  |  |  | MDMB-5'Me-INACA | 8.746 | 870 | - | 303 | - | - |
| FL24/0168 | 3 | 22-07-2024 | Powder | MDMB-FUBINACA | 10.441 | 884 | 10.371 | 357 | 70.08% | 67.84% |
|  |  |  |  | MDMB-INACA | 9.102 | 881 | 9.060 | 289 | 29.92% |  |

**Table S8.2.** Complete data for samples seized from Scottish prisons found positive for a tail-less SCRA/precursor without a corresponding SCRA(s). Samples are organized by seizure date. For security reasons, the prison where the sample was seized is represented by a number rather than the name of the establishment. Details of the GC–MS analysis are provided, including retention time (RT) of the compound, RT of the reference standard (ref std), and the mass (m/z) of the compound.

| **Sample ID** | **Prison ID** | **Seizure Date** | **Sample Type** | **Analysis Result** | **RT (mins)** | **R-Match (x/1000)** | **RT Ref Std (mins)** | **m/z (amu)** |
| --- | --- | --- | --- | --- | --- | --- | --- | --- |
| FL23/0123 | 11 | 07-02-2023 | Liquid | MDMB-INACA | 8.66 | 887 | 8.666 | 289 |
|  |  |  |  | Nicotine | 5.525 | 960 | - | 162 |
| FL23/0355-2 | 10 | 15-02-2023 | E-cigarette | MDMB-INACA | 8.593 | 871 | 8.592 | 289 |
|  |  |  |  | Bromazolam | 10.597 | 921 | 10.645 | 352 |
|  |  |  |  | Nicotine | 5.444 | 918 | - | 162 |
| FL23/0216 | 6 | 14-02-2023 | Tablet | MDMB-INACA | 8.663 | 864 | 8.666 | 289 |
|  |  |  |  | Amitriptyline | 6.374 | 947 | - | 277 |
| FL23/0316 | 5 | 27-02-2023 | Tablet | MDMB-INACA | 7.004 | 885 | 7.019 | 289 |
|  |  |  |  | Oxymetholone | 8.374 | 927 | 8.389 | 332 |
|  |  |  |  | Mestanolone | 7.499 | 911 | - | 304 |
| FL23/0128 | 11 | 16-03-2023 | Powder | MDMB-INACA | 8.657 | 855 | 8.666 | 289 |
| FL23/0362-2 | 10 | 31-03-2023 | Paper | MDMB-INACA | 8.594 | 851 | 8.601 | 289 |
| FL23/0201 | 3 | 07-04-2023 | Paper | MDMB-INACA | 8.658 | 888 | 8.666 | 289 |
| FL23/0110 | 1 | 01-05-2023 | Powder | MDMB-INACA | 8.666 | 854 | 8.666 | 289 |
|  |  |  |  | Benzocaine | 4.121 | 957 | - | 165 |
| FL23/0193-2 | 3 | 03-05-2023 | E-cigarette | MDMB-INACA | 8.664 | 890 | 8.666 | 289 |
|  |  |  |  | Δ^9^-THC | 8.683 | 914 | - | 314 |
| FL23/0293 | 3 | 11-05-2023 | E-cigarette | MDMB-INACA | 8.62 | 874 | 8.637 | 289 |
|  |  |  |  | Nicotine | 5.484 | 932 | - | 162 |
| FL23/0374-2 | 8 | 22-05-2023 | E-cigarette | MDMB-INACA | 8.593 | 866 | 8.592 | 289 |
|  |  |  |  | Nicotine | 5.45 | 904 | - | 162 |
|  |  |  |  | Δ^9^-THC | 8.626 | 906 | - | 314 |
| FL23/0367 | 10 | 03-06-2023 | Waxy material | MDMB-INACA | 8.601 | 853 | 8.601 | 289 |
|  |  |  |  | ADB-BUTINACA | 9.108 | 870 | 9.139 | 289 |
| FL23/0451-1 | 11 | 29-06-2023 | E-cigarette | MDMB-INACA | 8.533 | 876 | 8.531 | 289 |
|  |  |  |  | Nicotine | 5.363 | 947 | - | 162 |
| FL23/0451-2 | 11 | 29-06-2023 | E-cigarette | MDMB-INACA | 8.534 | 867 | 8.531 | 289 |
|  |  |  |  | Nicotine | 5.367 | 954 | - | 162 |
| FL23/0327-2 | 6 | 14-07-2023 | Powder | MDMB-INACA | 7.039 | 886 | 7.040 | 289 |
|  |  |  |  | Dimethylpentylone | 5.179 | 919 | - | 249 |
| FL23/0398 | 11 | 01-11-2023 | Paper | MDMB-INACA | 8.53 | 883 | 8.532 | 289 |
|  |  |  |  | ADB-BUTINACA | 9.018 | 873 | 9.026 | 330 |
|  |  |  |  | MDMB-5'Br-INACA | 9.505 | - | 9.449 | 368 |

**SECTION 9**

Complete datasets used for the creation of the heat map.

**Table S9.1.** US toxicological and seized sample analysis data used for the creation of the heat map compiled from NPS Discovery trend reports.

| **SCRA** | **Q4 2020** | **Q1 2021** | **Q2 2021** | **Q3 2021** | **Q4 2021** | **Q1 2022** | **Q2 2022** | **Q3 2022** | **Q4 2022** | **Q1 2023** | **Q2 2023** | **Q3 2023** | **Q4 2023** | **Q1 2024** | **Q2 2024** | **Q3 2024** |
| --- | --- | --- | --- | --- | --- | --- | --- | --- | --- | --- | --- | --- | --- | --- | --- | --- |
| MDMB-4en-PINACA | 54 | 45 | 31 | 15 | 5 | 15 | 8 | 4 | 11 | 37 | 18 | 4 | 16 | 11 | 49 | 42 |
| 5F-MDMB-PICA | 52 | 24 | 21 | 4 | 1 | 2 | 1 |  |  |  |  | 1 |  | 1 | 1 |  |
| 4F-MDMB-BUTINACA | 19 | 2 | 5 | 1 |  | 1 |  | 1 |  | 1 |  |  | 2 |  |  |  |
| 4F-MDMB-BUTICA | 19 | 11 | 7 | 4 |  | 1 |  |  |  |  |  |  | 3 |  |  |  |
| 5F-EDMB-PICA | 6 |  | 2 |  |  |  |  |  |  |  |  |  |  |  |  |  |
| ACHMINACA | 4 | 1 | 1 |  |  |  |  |  |  |  |  |  |  |  |  |  |
| Cumyl-4CN-BUTINACA | 3 |  |  |  |  |  | 2 |  |  |  |  |  |  | 1 |  |  |
| ADB-BUTINACA | 2 | 5 | 30 | 13 | 2 | 1 | 1 | 1 | 15 | 6 | 2 | 1 | 5 |  | 8 | 1 |
| ADB-4en-PINACA |  | 1 | 4 | 1 |  |  |  |  | 1 |  |  |  |  | 2 | 6 | 6 |
| 5F-MDMB-PINACA |  | 4 | 4 |  | 3 |  | 2 |  |  |  | 1 |  |  | 5 | 9 | 14 |
| 4F-ABUTINACA |  | 3 | 6 | 1 |  |  |  |  |  |  |  |  |  |  |  |  |
| 5F-EMB-PICA |  |  |  |  |  |  |  |  |  |  |  |  |  |  |  |  |
| ADB-HEXINACA |  |  | 1 | 1 | 1 |  |  |  |  |  |  |  |  | 1 |  |  |
| 5F-3,5-AB-PFUPPYCA |  |  |  |  |  |  |  |  |  |  |  |  |  |  |  |  |
| 3,5-ADB-4en-PFUPPYCA |  |  |  |  |  |  |  |  |  |  |  |  |  |  |  |  |
| ADB-FUBIATA |  |  |  |  | 2 |  |  |  |  |  |  |  | 1 |  |  |  |
| ADB-5'Br-INACA |  |  |  |  |  |  |  |  |  |  |  |  |  |  |  |  |
| MDMB-5'Br-INACA |  |  |  |  |  |  |  |  |  |  |  |  |  | 1 |  |  |
| BZO-HEXOXIZID |  |  |  |  |  |  |  |  |  |  |  |  |  |  |  |  |
| MMB-FUBINACA |  |  |  |  | 1 |  | 2 | 1 |  |  |  |  |  |  |  |  |
| BZO-CHMOXIZID |  |  |  |  | 1 |  |  |  |  |  |  |  |  |  |  |  |
| CH-PIATA |  |  |  |  |  |  |  |  | 1 |  |  |  |  |  |  |  |
| BZO-4en-POXIZID |  |  |  |  |  |  |  |  |  |  |  |  |  |  |  |  |
| BZO-POXIZID |  |  |  |  |  |  |  | 2 |  |  |  |  |  |  |  |  |
| 5F-BZO-POXIZID |  |  |  |  |  |  |  |  |  |  |  |  |  |  |  |  |
| ADB-5'Br-BUTINACA |  |  |  |  |  |  |  | 1 |  | 1 |  |  |  |  |  |  |
| ADB-INACA |  |  |  |  |  |  |  |  | 2 | 1 | 1 |  | 5 |  | 2 | 2 |
| Cumyl-TsINACA |  |  |  |  |  |  |  |  | 1 |  |  |  |  |  |  |  |
| MDMB-INACA |  |  |  |  |  |  |  |  |  | 7 | 1 |  | 7 | 4 | 8 | 14 |
| ADB-5'Br-PINACA |  |  |  |  |  |  |  |  |  | 1 |  |  |  |  |  |  |
| MDMB-BUTINACA |  |  |  |  |  |  |  |  |  |  | 1 |  | 1 | 1 | 2 | 11 |
| CHO-4'Me-5'Br-FUBOXPYRA |  |  |  |  |  |  |  |  |  |  | 1 |  |  |  |  | 1 |
| AB-CHMINACA |  |  |  |  |  |  |  |  |  |  |  |  |  |  | 11 |  |
| AB-INACA |  |  |  |  |  |  |  |  |  |  |  |  |  |  |  |  |
| MDMB-5'Me-INACA |  |  |  |  |  |  |  |  |  |  |  |  |  |  |  | 2 |
| MDMB-FUBINACA |  |  |  |  |  |  |  |  |  |  |  |  |  |  |  |  |
| MMB-4en-PINACA |  |  |  |  |  |  |  |  |  |  |  |  |  | 1 |  |  |
| CH-FUBIATA |  |  |  |  |  |  |  |  |  |  |  |  |  | 1 |  |  |
| MDMB-ICA |  |  |  |  |  |  |  |  |  |  |  |  |  |  | 1 |  |
| Total (Positive) | 114 | 70 | 88 | 40 | 16 | 20 | 16 | 10 | 31 | 54 | 25 | 6 | 40 | 29 | 97 | 93 |

**Table S9.2.** Scottish prisons seized sample data used for the creation of the heat map.

| **SCRA** | **Q4 2020** | **Q1 2021** | **Q2 2021** | **Q3 2021** | **Q4 2021** | **Q1 2022** | **Q2 2022** | **Q3 2022** | **Q4 2022** | **Q1 2023** | **Q2 2023** | **Q3 2023** | **Q4 2023** | **Q1 2024** | **Q2 2024** | **Q3 2024** |
| --- | --- | --- | --- | --- | --- | --- | --- | --- | --- | --- | --- | --- | --- | --- | --- | --- |
| MDMB-4en-PINACA | 9 | 34 | 26 | 26 | 8 | 3 | 1 | 2 | 3 | 24 | 36 | 35 | 9 | 6 | 8 | 2 |
| 5F-MDMB-PICA |  | 3 | 1 | 1 | 10 | 1 |  | 1 |  |  |  |  |  |  |  |  |
| 4F-MDMB-BUTINACA |  | 2 |  | 5 | 1 | 1 | 2 | 1 | 1 |  |  | 1 |  |  | 2 |  |
| 4F-MDMB-BUTICA |  | 1 | 1 | 3 | 11 | 1 |  | 1 | 5 |  |  | 1 |  |  |  |  |
| 5F-EDMB-PICA |  |  |  |  |  |  |  |  |  |  |  |  |  |  | 1 | 1 |
| ACHMINACA |  |  |  |  |  |  |  |  |  |  |  |  |  |  |  |  |
| Cumyl-4CN-BUTINACA |  | 2 |  |  |  | 1 |  |  |  |  |  |  |  |  |  |  |
| ADB-BUTINACA |  | 67 | 21 | 102 | 94 | 15 | 22 | 36 | 14 | 4 | 19 | 15 | 7 |  |  |  |
| ADB-4en-PINACA | 2 | 10 | 1 | 3 | 4 | 1 | 8 | 6 | 2 | 1 | 1 |  | 2 |  |  |  |
| 5F-MDMB-PINACA | 1 |  |  |  |  |  |  | 1 |  | 1 |  |  |  |  |  |  |
| 4F-ABUTINACA |  |  |  | 2 |  |  |  | 1 |  |  |  | 1 |  |  |  |  |
| 5F-EMB-PICA |  | 5 |  | 2 |  |  |  |  |  |  |  |  |  |  |  |  |
| ADB-HEXINACA |  |  | 3 | 3 | 10 | 8 | 12 | 3 | 3 |  |  | 1 |  |  |  |  |
| 5F-3,5-AB-PFUPPYCA |  |  |  | 6 | 2 | 1 |  |  |  |  |  |  |  |  |  |  |
| 3,5-ADB-4en-PFUPPYCA |  |  |  | 6 | 2 | 1 |  |  |  |  |  |  |  |  |  |  |
| ADB-FUBIATA |  |  |  |  | 3 |  | 1 | 2 | 1 | 1 |  |  |  |  |  |  |
| ADB-5'Br-INACA |  |  |  |  | 10 |  |  |  |  |  |  |  |  |  |  |  |
| MDMB-5'Br-INACA |  |  |  |  | 9 |  |  |  |  |  |  |  | 1 |  |  |  |
| BZO-HEXOXIZID |  |  |  |  | 2 |  | 1 |  |  |  |  |  | 1 |  |  |  |
| MMB-FUBINACA |  |  |  |  |  |  |  |  |  |  |  |  |  |  |  |  |
| BZO-CHMOXIZID |  |  |  |  |  |  |  |  |  |  |  |  |  |  |  |  |
| CH-PIATA |  |  |  |  |  | 3 | 2 | 5 | 1 |  |  |  |  |  |  |  |
| BZO-4en-POXIZID |  |  |  |  |  |  | 1 |  |  |  |  |  |  |  |  |  |
| BZO-POXIZID |  |  |  |  |  |  |  |  |  |  |  |  |  |  |  |  |
| 5F-BZO-POXIZID |  |  |  |  |  |  |  | 1 |  |  |  |  |  |  | 8 | 3 |
| ADB-5'Br-BUTINACA |  |  |  |  |  |  |  |  | 10 |  | 1 |  |  |  | 1 | 1 |
| ADB-INACA |  |  |  |  |  |  |  |  |  |  |  |  |  |  |  |  |
| Cumyl-TsINACA |  |  |  |  |  |  |  |  |  |  |  |  |  |  |  |  |
| MDMB-INACA |  |  |  |  |  |  |  |  |  | 16 | 28 | 33 | 7 | 5 |  |  |
| ADB-5'Br-PINACA |  |  |  |  |  |  |  |  |  |  |  |  |  |  |  |  |
| MDMB-BUTINACA |  |  |  |  |  |  |  |  |  |  | 2 | 4 | 4 |  |  |  |
| CHO-4'Me-5'Br-FUBOXPYRA |  |  |  |  |  |  |  |  |  |  |  |  |  |  |  |  |
| AB-CHMINACA |  |  |  |  |  |  |  |  |  |  |  | 1 | 1 | 1 | 2 | 2 |
| AB-INACA |  |  |  |  |  |  |  |  |  |  |  | 1 | 1 | 1 |  | 3 |
| MDMB-5'Me-INACA |  |  |  |  |  |  |  |  |  |  |  | 1 | 2 | 2 |  |  |
| MDMB-FUBINACA |  |  |  |  |  |  |  |  |  |  |  |  |  | 1 |  |  |
| MMB-4en-PINACA |  |  |  |  |  |  |  |  |  |  |  |  |  |  |  |  |
| CH-FUBIATA |  |  |  |  |  |  |  |  |  |  |  |  |  |  |  |  |
| MDMB-ICA |  |  |  |  |  |  |  |  |  |  |  |  |  |  |  |  |
| Total (Positive) | 12 | 110 | 48 | 131 | 137 | 30 | 45 | 47 | 36 | 35 | 55 | 45 | 17 | 8 | 10 | 4 |

**SECTION 10**

Complete statistical analysis results for the *in vitro* CB_1_ receptor activity

**Table S10.1.** Comparison of efficacy and potency from the β-arrestin 2 assay between the 5, 25, 50, and 75% mixtures of the final SCRA (ADB-BUTINACA or MDMB-4en-PINACA) and precursor (ADB-INACA or MDMB-INACA) and 100% of the final SCRA and 100% of the precursor. This is based on the data analyzed using the total concentration of the SCRAs in the mixture (combined concentrations of the precursor and the final product). The 100% ADB-INACA could not be used for comparison as it did not reach a plateau. P values are from Brown-Forsythe and Welch ANOVA tests with CP55,940 as a reference.

| **Comparisons** | | **Efficacy (E_max_)** | | | **Potency (EC_50_)** | | |
| --- | --- | --- | --- | --- | --- | --- | --- |
| **Mixture 1** | **Mixture 2** | **Mean Diff** | **P value** | **Significant?** | **Mean Diff** | **P value** | **Significant?** |
| 100% ADB-BUTINACA | 5% ADB-BUTINACA | 98.60 | 0.3607 | No | -1.293 | 0.0236 | Yes |
|  | 25% ADB-BUTINACA | 66.35 | 0.8146 | No | -0.7564 | 0.0241 | Yes |
|  | 50% ADB-BUTINACA | 155.6 | 0.1747 | No | -0.3368 | 0.2479 | No |
|  | 75% ADB-BUTINACA | 70.25 | 0.7372 | No | -0.1874 | 0.7634 | No |
| 75% ADB-BUTINACA | 5% ADB-BUTINACA | 28.35 | 0.9614 | No | -1.106 | 0.0044 | Yes |
|  | 25% ADB-BUTINACA | -3.908 | >0.9999 | No | -0.5690 | 0.0413 | Yes |
|  | 50% ADB-BUTINACA | 85.35 | 0.3900 | No | -0.1494 | 0.7848 | No |
| 50% ADB-BUTINACA | 5% ADB-BUTINACA | -57.00 | 0.4969 | No | -0.9561 | 0.0050 | Yes |
|  | 25% ADB-BUTINACA | -89.26 | 0.4232 | No | -0.4196 | 0.0957 | No |
| 25% ADB-BUTINACA | 5% ADB-BUTINACA | 32.25 | 0.9588 | No | -0.5365 | 0.0952 | No |
| 100% MDMB-INACA | 5% MDMB-4en-PINACA | 539.7 | 0.0515 | No | -2.754 | 0.0007 | Yes |
|  | 25% MDMB-4en-PINACA | 560.7 | 0.0189 | Yes | -3.568 | <0.0001 | Yes |
|  | 50% MDMB-4en-PINACA | 545.8 | 0.0179 | Yes | -3.610 | <0.0001 | Yes |
|  | 75% MDMB-4en-PINACA | 510.6 | 0.0257 | Yes | -3.744 | <0.0001 | Yes |
|  | 100% MDMB-4en-PINACA | 536.7 | 0.0225 | Yes | -3.732 | <0.0001 | Yes |
| 100% MDMB-4en-PINACA | 5% MDMB-4en-PINACA | -3.010 | >0.9999 | No | -0.9777 | 0.0154 | Yes |
|  | 25% MDMB-4en-PINACA | -24.05 | 0.9998 | No | -0.1642 | 0.9052 | No |
|  | 50% MDMB-4en-PINACA | -9.133 | >0.9999 | No | -0.1222 | 0.9553 | No |
|  | 75% MDMB-4en-PINACA | 26.09 | 0.9997 | No | 0.01148 | >0.9999 | No |
| 75% MDMB-4en-PINACA | 5% MDMB-4en-PINACA | -29.10 | 0.9917 | No | -0.9891 | 0.0156 | Yes |
|  | 25% MDMB-4en-PINACA | -50.14 | 0.9365 | No | -0.1757 | 0.8777 | No |
|  | 50% MDMB-4en-PINACA | -35.22 | 0.9855 | No | -0.1337 | 0.9328 | No |
| 50% MDMB-4en-PINACA | 5% MDMB-4en-PINACA | 6.123 | >0.9999 | No | -0.8554 | 0.0035 | Yes |
|  | 25% MDMB-4en-PINACA | -14.92 | >0.9999 | No | -0.04198 | >0.9999 | No |
| 25% MDMB-4en-PINACA | 5% MDMB-4en-PINACA | 21.04 | 0.9991 | No | -0.8135 | 0.0262 | Yes |

**Table S10.2.** Comparison of efficacy and potency from the β-arrestin 2 assay between the 5, 25, 50, and 75% mixtures of the final SCRA (ADB-BUTINACA or MDMB-4en-PINACA) and precursor (ADB-INACA or MDMB-INACA) and 100% of the final SCRA and 100% of the precursor. This is based on the data analyzed using only the concentration of the final product in the mixture. The 100% ADB-INACA could not be used for comparison as the EC_50_ and E_max_ could not be calculated since it did not reach a plateau. P values are from Brown-Forsythe and Welch ANOVA tests with CP55,940 as a reference.

| **Comparisons** | | **Efficacy (E_max_)** | | | **Potency (EC_50_)** | | |
| --- | --- | --- | --- | --- | --- | --- | --- |
| **Mixture 1** | **Mixture 2** | **Mean Diff** | **P value** | **Significant?** | **Mean Diff** | **P value** | **Significant?** |
| 100% ADB-BUTINACA | 5% ADB-BUTINACA | 76.63 | 0.6778 | No | -0.1952 | 0.6542 | No |
|  | 25% ADB-BUTINACA | 52.66 | 0.9836 | No | -0.3902 | 0.2307 | No |
|  | 50% ADB-BUTINACA | 144.9 | 0.2485 | No | -0.3140 | 0.3480 | No |
|  | 75% ADB-BUTINACA | 60.28 | 0.9451 | No | -0.3504 | 0.2963 | No |
| 75% ADB-BUTINACA | 5% ADB-BUTINACA | 16.36 | >0.9999 | No | 0.1552 | 0.6696 | No |
|  | 25% ADB-BUTINACA | -7.612 | >0.9999 | No | -0.03975 | >0.9999 | No |
|  | 50% ADB-BUTINACA | 84.62 | 0.6022 | No | 0.03638 | >0.9999 | No |
| 50% ADB-BUTINACA | 5% ADB-BUTINACA | -68.26 | 0.5354 | No | 0.1188 | 0.7783 | No |
|  | 25% ADB-BUTINACA | -92.23 | 0.5900 | No | -0.07614 | 0.9988 | No |
| 25% ADB-BUTINACA | 5% ADB-BUTINACA | 23.97 | 0.9996 | No | -6.006 | 0.5306 | No |
| 100% MDMB-INACA | 5% MDMB-4en-PINACA | 550.8 | 0.0242 | Yes | -3.782 | 0.0003 | Yes |
|  | 25% MDMB-4en-PINACA | 569.4 | 0.0218 | Yes | -3.829 | <0.0001 | Yes |
|  | 50% MDMB-4en-PINACA | 558.2 | 0.0203 | Yes | -3.557 | <0.0001 | Yes |
|  | 75% MDMB-4en-PINACA | 521.9 | 0.0287 | Yes | -3.502 | <0.0001 | Yes |
|  | 100% MDMB-4en-PINACA | 536.7 | 0.0264 | Yes | -3.732 | <0.0001 | Yes |
| 100% MDMB-4en-PINACA | 5% MDMB-4en-PINACA | -14.08 | >0.9999 | No | 0.05008 | >0.9999 | No |
|  | 25% MDMB-4en-PINACA | -32.68 | 0.9992 | No | 0.09668 | 0.9989 | No |
|  | 50% MDMB-4en-PINACA | -21.46 | >0.9999 | No | -0.1753 | 0.8343 | No |
|  | 75% MDMB-4en-PINACA | 14.76 | >0.9999 | No | -0.2300 | 0.7366 | No |
| 75% MDMB-4en-PINACA | 5% MDMB-4en-PINACA | -28.84 | 0.9980 | No | 0.2801 | 0.4108 | No |
|  | 25% MDMB-4en-PINACA | -47.44 | 0.9799 | No | 0.3267 | 0.4112 | No |
|  | 50% MDMB-4en-PINACA | -36.22 | 0.9946 | No | 0.05468 | >0.9999 | No |
| 50% MDMB-4en-PINACA | 5% MDMB-4en-PINACA | 7.383 | >0.9999 | No | 0.2254 | 0.3587 | No |
|  | 25% MDMB-4en-PINACA | -11.21 | >0.9999 | No | 0.2720 | 0.4459 | No |
| 25% MDMB-4en-PINACA | 5% MDMB-4en-PINACA | 18.60 | >0.9999 | No | -0.04660 | >0.9999 | No |

**Table S10.3.** Comparison of efficacy and potency from the AequoScreen^®^ assay between the 5, 25, 50, and 75% mixtures of the final SCRA (ADB-BUTINACA or MDMB-4en-PINACA) and precursor (ADB-INACA or MDMB-INACA) and 100% of the final SCRA and 100% of the precursor. This is based on the data analyzed using the total concentration of the SCRAs in the mixture (combined concentrations of the precursor and the final product). The 100% ADB-INACA could not be used for comparison as it did not reach a plateau. P values are from Brown-Forsythe and Welch ANOVA tests with JWH-018 as a reference.

| **Comparisons** | | **Efficacy (E_max_)** | | | **Potency (EC_50_)** | | |
| --- | --- | --- | --- | --- | --- | --- | --- |
| **Mixture 1** | **Mixture 2** | **Mean Diff** | **P value** | **Significant?** | **Mean Diff** | **P value** | **Significant?** |
| 5% ADB-BUTINACA | 25% ADB-BUTINACA | -1.438 | 0.9911 | No | 0.4358 | 0.0108 | Yes |
|  | 50% ADB-BUTINACA | -1.623 | 0.9942 | No | 0.9592 | 0.0056 | Yes |
|  | 75% ADB-BUTINACA | 4.939 | 0.2564 | No | 1.023 | 0.0003 | Yes |
|  | 100% ADB-BUTINACA | 4.610 | 0.3335 | No | 1.125 | 0.0003 | Yes |
| 25% ADB-BUTINACA | 50% ADB-BUTINACA | -0.1846 | >0.9999 | No | 0.5234 | 0.0353 | Yes |
|  | 75% ADB-BUTINACA | 6.377 | 0.1352 | No | 0.5875 | 0.0037 | Yes |
|  | 100% ADB-BUTINACA | 6.049 | 0.1771 | No | 0.6896 | 0.0025 | Yes |
| 50% ADB-BUTINACA | 75% ADB-BUTINACA | 6.562 | 0.2724 | No | 0.06418 | 0.9845 | No |
|  | 100% ADB-BUTINACA | 6.233 | 0.3195 | No | 0.1662 | 0.4947 | No |
| 75% ADB-BUTINACA | 100% ADB-BUTINACA | -0.3282 | >0.9999 | No | 0.1021 | 0.6911 | No |
| 100% MDMB-INACA | 5% MDMB-4en-PINACA | 6.744 | 0.2476 | No | 1.999 | <0.0001 | Yes |
|  | 25% MDMB-4en-PINACA | 2.036 | 0.9963 | No | 2.687 | 0.0003 | Yes |
|  | 50% MDMB-4en-PINACA | -0.07656 | >0.9999 | No | 2.853 | <0.0001 | Yes |
|  | 75% MDMB-4en-PINACA | 9.029 | 0.2254 | No | 2.927 | 0.0004 | Yes |
|  | 100% MDMB-4en-PINACA | 8.229 | 0.1302 | No | 3.402 | <0.0001 | Yes |
| 25% MDMB-4en-PINACA | 5% MDMB-4en-PINACA | 4.708 | 0.5973 | No | -0.6878 | 0.0196 | Yes |
|  | 50% MDMB-4en-PINACA | -2.113 | 0.9892 | No | 0.1667 | 0.5674 | No |
|  | 75% MDMB-4en-PINACA | 6.993 | 0.4482 | No | 0.2398 | 0.4977 | No |
|  | 100% MDMB-4en-PINACA | 6.193 | 0.3786 | No | 0.7148 | 0.0078 | Yes |
| 50% MDMB-4en-PINACA | 5% MDMB-4en-PINACA | 6.820 | 0.1577 | No | -0.8545 | 0.0012 | Yes |
|  | 75% MDMB-4en-PINACA | 9.106 | 0.2216 | No | 0.07309 | 0.9950 | No |
|  | 100% MDMB-4en-PINACA | 8.306 | 0.0752 | No | 0.5481 | 0.0092 | Yes |
| 75% MDMB-4en-PINACA | 5% MDMB-4en-PINACA | -2.285 | 0.9861 | No | -0.9275 | 0.0139 | Yes |
|  | 100% MDMB-4en-PINACA | -0.8002 | >0.9999 | No | 0.4750 | 0.0975 | No |
| 5% MDMB-4en-PINACA | 100% MDMB-4en-PINACA | 1.485 | 0.9935 | No | 1.403 | 0.0002 | Yes |

**Table S10.4.** Comparison of efficacy and potency from the AequoScreen^®^ assay between the 5, 25, 50, and 75% mixtures of the final SCRA (ADB-BUTINACA or MDMB-4en-PINACA) and precursor (ADB-INACA or MDMB-INACA) and 100% of the final SCRA and 100% of the precursor. This is based on the data analyzed using only the concentration of the final product in the mixture. The 100% ADB-INACA could not be used for comparison as the EC_50_ and E_max_ could not be calculated since it did not reach a plateau. P values are from Brown-Forsythe and Welch ANOVA tests with JWH-018 as a reference.

| **Comparisons** | | **Efficacy (E_max_)** | | | **Potency (EC_50_)** | | |
| --- | --- | --- | --- | --- | --- | --- | --- |
| **Mixture 1** | **Mixture 2** | **Mean Diff** | **P value** | **Significant?** | **Mean Diff** | **P value** | **Significant?** |
| 5% ADB-BUTINACA | 25% ADB-BUTINACA | -1.439 | 0.9911 | No | -0.2631 | 0.0649 | No |
|  | 50% ADB-BUTINACA | -1.623 | 0.9942 | No | -0.04075 | 0.9996 | No |
|  | 75% ADB-BUTINACA | 4.938 | 0.2563 | No | -0.1527 | 0.2820 | No |
|  | 100% ADB-BUTINACA | 4.609 | 0.3336 | No | -0.1759 | 0.2342 | No |
| 25% ADB-BUTINACA | 50% ADB-BUTINACA | -0.1847 | >0.9999 | No | 0.2224 | 0.2999 | No |
|  | 75% ADB-BUTINACA | 6.377 | 0.1352 | No | 0.1105 | 0.5994 | No |
|  | 100% ADB-BUTINACA | 6.048 | 0.1771 | No | 0.08720 | 0.8341 | No |
| 50% ADB-BUTINACA | 75% ADB-BUTINACA | 6.562 | 0.2723 | No | -0.1119 | 0.7914 | No |
|  | 100% ADB-BUTINACA | 6.233 | 0.3195 | No | -0.1352 | 0.6784 | No |
| 75% ADB-BUTINACA | 100% ADB-BUTINACA | -0.3288 | >0.9999 | No | -0.02327 | >0.9999 | No |
| 100% MDMB-INACA | 5% MDMB-4en-PINACA | 2.037 | 0.9963 | No | 3.289 | 0.0002 | Yes |
|  | 25% MDMB-4en-PINACA | -0.07601 | >0.9999 | No | 3.155 | <0.0001 | Yes |
|  | 50% MDMB-4en-PINACA | 9.030 | 0.2254 | No | 3.052 | 0.0004 | Yes |
|  | 75% MDMB-4en-PINACA | 6.745 | 0.2475 | No | 3.300 | <0.0001 | Yes |
|  | 100% MDMB-4en-PINACA | 8.229 | 0.1302 | No | 3.402 | <0.0001 | Yes |
| 25% MDMB-4en-PINACA | 5% MDMB-4en-PINACA | -2.113 | 0.9892 | No | -0.1344 | 0.7513 | No |
|  | 50% MDMB-4en-PINACA | 6.993 | 0.4482 | No | -0.2373 | 0.5068 | No |
|  | 75% MDMB-4en-PINACA | 4.708 | 0.5972 | No | 0.01112 | >0.9999 | No |
|  | 100% MDMB-4en-PINACA | 6.192 | 0.3786 | No | 0.1123 | 0.8775 | No |
| 50% MDMB-4en-PINACA | 5% MDMB-4en-PINACA | 9.106 | 0.2216 | No | -0.1030 | 0.9555 | No |
|  | 75% MDMB-4en-PINACA | 6.821 | 0.1576 | No | 0.1455 | 0.4373 | No |
|  | 100% MDMB-4en-PINACA | 8.305 | 0.0752 | No | 0.2467 | 0.1427 | No |
| 75% MDMB-4en-PINACA | 5% MDMB-4en-PINACA | -2.285 | 0.9861 | No | 0.2485 | 0.3896 | No |
|  | 100% MDMB-4en-PINACA | -0.8008 | >0.9999 | No | 0.3497 | 0.2062 | No |
| 5% MDMB-4en-PINACA | 100% MDMB-4en-PINACA | 1.485 | 0.9935 | No | 0.1012 | 0.7723 | No |

**Table S10.5.** Comparison of efficacy and potency from the β-arrestin 2 assay between the seizures and references standards of MDMB-INACA and the products from the replicated synthesis using different reaction conditions (RT and 5 h, 70˚C and 5 h, and 70˚C and 10 h) and the reference standard of the final product (ADB-BUTINACA or MDMB-4en-PINACA). Statistical comparison was not possible for the ADB-INACA seizures and reference standards as they did not reach a plateau. P values are from Brown-Forsythe and Welch ANOVA tests with CP55,940 as a reference.

| **Comparisons** | | **Efficacy (E_max_)** | | | **Potency (EC_50_)** | | |
| --- | --- | --- | --- | --- | --- | --- | --- |
| **Compound 1** | **Compound 2** | **Mean Diff** | **P value** | **Significant?** | **Mean Diff** | **P value** | **Significant?** |
| ADB-BUTINACA RT, 5h | ADB-BUTINACA 70˚C, 5h | -144.5 | 0.4101 | No | -0.1532 | 0.9053 | No |
|  | ADB-BUTINACA 70˚C, 10h | 95.50 | 0.6128 | No | -0.08008 | 0.9937 | No |
|  | ADB-BUTINACA ref | -24.63 | 0.9993 | No | 0.004590 | >0.9999 | No |
| ADB-BUTINACA 70˚C, 5h | ADB-BUTINACA 70˚C, 10h | 240.0 | 0.1396 | No | 0.07316 | 0.9977 | No |
|  | ADB-BUTINACA ref | 119.9 | 0.7199 | No | 0.1578 | 0.9596 | No |
| ADB-BUTINACA 70˚C, 10h | ADB-BUTINACA ref | -120.1 | 0.6508 | No | 0.08467 | 0.9978 | No |
| MDMB-INACA (ref) | MDMB-INACA (seizure) | -144.0 | 0.0749 | No | 0.04053 | >0.9999 | No |
| MDMB-4en-PINACA RT, 5h | MDMB-4en-PINACA 70˚C, 5h | 137.0 | 0.2995 | No | 0.3000 | 0.5238 | No |
|  | MDMB-4en-PINACA 70˚C, 10h | 44.02 | 0.9501 | No | 0.2756 | 0.5151 | No |
|  | MDMB-4en-PINACA ref | 77.44 | 0.7230 | No | 0.3158 | 0.4531 | No |
| MDMB-4en-PINACA 70˚C, 5h | MDMB-4en-PINACA 70˚C, 10h | -92.94 | 0.5162 | No | -0.02434 | >0.9999 | No |
|  | MDMB-4en-PINACA ref | -59.52 | 0.8466 | No | 0.01589 | >0.9999 | No |
| MDMB-4en-PINACA 70˚C, 10h | MDMB-4en-PINACA ref | 33.42 | 0.9802 | No | 0.04023 | 0.9998 | No |

**Table S10.6.** Comparison of efficacy and potency from the AequoScreen^®^ assay between the seizures and references standards of MDMB-INACA and the products from the replicated synthesis using different reaction conditions (RT and 5 h, 70˚C and 5 h, and 70˚C and 10 h) and the reference standard of the final product (ADB-BUTINACA or MDMB-4en-PINACA). Statistical comparison was not possible for the ADB-INACA seizures and reference standards as they did not reach a plateau. P values are from Brown-Forsythe and Welch ANOVA tests with CP55,940 as a reference.

| **Comparisons** | | **Efficacy (E_max_)** | | | **Potency (EC_50_)** | | |
| --- | --- | --- | --- | --- | --- | --- | --- |
| **Compound 1** | **Compound 2** | **Mean Diff** | **P value** | **Significant?** | **Mean Diff** | **P value** | **Significant?** |
| ADB-BUTINACA RT, 5h | ADB-BUTINACA 70˚C, 5h | -1.872 | 0.8408 | No | -0.05976 | 0.8583 | No |
|  | ADB-BUTINACA 70˚C, 10h | -0.3193 | >0.9999 | No | 0.03707 | 0.9818 | No |
|  | ADB-BUTINACA ref | -2.492 | 0.6073 | No | -0.03612 | 0.9728 | No |
| ADB-BUTINACA 70˚C, 5h | ADB-BUTINACA 70˚C, 10h | 1.553 | 0.9249 | No | 0.09684 | 0.5848 | No |
|  | ADB-BUTINACA ref | -0.6196 | 0.9986 | No | 0.02364 | 0.9967 | No |
| ADB-BUTINACA 70˚C, 10h | ADB-BUTINACA ref | -2.173 | 0.7348 | No | -0.07320 | 0.7433 | No |
| MDMB-INACA (ref) | MDMB-INACA (seizure) | 1.797 | 0.9770 | No | 0.1683 | 0.1620 | No |
| MDMB-4en-PINACA RT, 5h | MDMB-4en-PINACA 70˚C, 5h | 0.7081 | 0.9811 | No | 0.05800 | 0.7021 | No |
|  | MDMB-4en-PINACA 70˚C, 10h | -0.4688 | 0.9981 | No | -0.02169 | 0.9936 | No |
|  | MDMB-4en-PINACA ref | -1.547 | 0.7766 | No | -0.03389 | 0.9589 | No |
| MDMB-4en-PINACA 70˚C, 5h | MDMB-4en-PINACA 70˚C, 10h | -1.177 | 0.9150 | No | -0.07969 | 0.5652 | No |
|  | MDMB-4en-PINACA ref | -2.255 | 0.5705 | No | -0.09189 | 0.4955 | No |
| MDMB-4en-PINACA 70˚C, 10h | MDMB-4en-PINACA ref | -1.078 | 0.9568 | No | -0.01220 | 0.9999 | No |
